# Combating climate change with matching-commitment agreements

**DOI:** 10.1038/s41598-020-63446-1

**Published:** 2020-06-19

**Authors:** Chai Molina, Erol Akçay, Ulf Dieckmann, Simon A. Levin, Elena A. Rovenskaya

**Affiliations:** 1 International Institute for Applied Systems Analysis, Laxenburg, A-2361 Austria; 2 Department of Ecology and Evolutionary Biology, Princeton University, Princeton, 08544 NJ USA; 3 Department of Biology, University of Pennsylvania, Philadelphia, PA 19104 USA; 4 Department of Evolutionary Studies of Biosystems, The Graduate University for Advanced Studies (Sokendai), Hayama, Kanagawa 240-0193 Japan; 5 Beijer Institute of Ecological Economics, SE-104 05, Stockholm, Sweden; 6 Resources for the Future, Washington, DC 20036 USA; 7 Faculty of Computational Mathematics and Cybernetics, Lomonosov Moscow State University, Leninskiye Gory 1-52, Moscow, 119234 Russia

**Keywords:** Climate-change mitigation, Applied mathematics

## Abstract

Countries generally agree that global greenhouse gas emissions are too high, but prefer other countries reduce emissions rather than reducing their own. The Paris Agreement is intended to solve this collective action problem, but is likely insufficient. One proposed solution is a matching-commitment agreement, through which countries can change each other’s incentives by committing to conditional emissions reductions, before countries decide on their unconditional reductions. Here, we study matching-commitment agreements between two heterogeneous countries. We find that such agreements (1) incentivize both countries to make matching commitments that in turn incentivize efficient emissions reductions, (2) reduce emissions from those expected without an agreement, and (3) increase both countries’ welfare. Matching-commitment agreements are attractive because they do not require a central enforcing authority and only require countries to fulfil their promises; countries are left to choose their conditional and unconditional emissions reductions according to their own interests.

## Introduction

Anthropogenic emissions of greenhouse gases (GHGs) have been identified as comprising a key factor in climate change^1^. In the Paris Agreement, countries overwhelmingly agree to limiting “the increase in the global average temperature to well below 2 °C […] and pursuing efforts to limit the temperature increase to 1.5 °C above pre-industrial levels”^2^. To do so, they must curb GHG emissions; yet according to recent assessments of present and future global GHG emissions, the Earth is still expected to warm by more than 2 °C^3–6^.

Achieving the necessary reduction in GHG emissions is difficult, primarily because it is an instance of Hardin’s classic “tragedy of the commons”^7^: each country benefits from reduced *global* emissions, but curbing *its own* emissions carries an economic cost. Countries thus prefer to free-ride on the emissions abatement (i.e., reduction of emissions) of others over reducing their own emissions^8,9^. The implied tragedy is that if countries pursue their own interests in this way, everyone will be worse off than if they had cooperated and acted in their collective interest.

To manage shared resources (or to resolve other collective action problems) within a single country, rules are typically agreed upon and enforced, making cooperation the rational choice by shaping individual incentives. In contrast, the Paris Agreement details countries’ voluntarily pledged emission reductions in the hope of averting a “climate tragedy”, but this agreement will likely fall short of its goal. For example, even if countries reduced their emissions by the amounts they pledged, the Earth would still be an estimated 3.2 °C warmer by 2100 (despite the agreement’s goal of limiting global warming to no more than 1.5 °C)^10^. The cause for this discrepancy between the Paris Agreement’s goal and individual countries’ abatement targets is that, because countries are sovereign, their participation must be voluntary. Thus, countries chose their own abatement targets, and had little incentive to make very ambitious pledges. Moreover, the abatement targets that countries have pledged are not legally binding, so countries are not incentivized to fulfill them; consequently some countries’ current policies are insufficient to fulfill even their modest pledges^10^. So, while, in principle, “prompt and substantial reductions in greenhouse gas emissions on a global scale” could limit warming to meet the 1.5 °C target set in Paris, at 2015 rates of emissions the global carbon budget for this goal will be exhausted already in 2021^11^.

Seeking possible mechanisms for lowering global emissions, many researchers have turned to game theory (see ref. ^12^ for a review). Most of the resulting proposals involve establishing a central authority that can punish non-participation and noncompliance or otherwise redistribute money or resources (either directly or by approving sanctions imposed by countries)^8,13–16^. But enforcement is difficult to achieve in practice, because, as pointed out by Nordhaus^8^, it conflicts with the principles of countries’ sovereignty, equality and right to manage internal affairs without intervention, first established in the 1648 Treaty of Westphalia. Consequently, Nordhaus has suggested that international trade law be amended to allow sanctions against non-participants, while prohibiting retaliation by the latter^8^. This would require the comprehensive renegotiation of trade laws, which will be a difficult and lengthy process. Moreover, Barrett also stresses the importance of enforcement, but warns that even if countries agree to a treaty with strong enforcement mechanisms, they will likely do so at the expense of lowering their abatement targets “to ensure that punishments are not imposed”^14^. Hence, solutions that encourage countries to cooperate without encroaching on their sovereignty would be desirable, since the global carbon budget for 1.5 °C warming is quickly running out.

Barrett^17,18^ was perhaps the first to propose applying Guttman’s^19,20^ idea of matching contributions to lower GHG emissions and help mitigate climate change. More recently, Boadway *et al*.^21^ applied Guttman’s^19,20^ matching scheme to Finus’s^22^ Global Emission Game: every country’s emissions abatement is divided into an unconditional and a conditional abatement. Countries simultaneously declare **matching factors**, which define how much conditional abatement they will contribute for a unit of *un*conditional abatement contributed by each other country. (The literature typically refers to a country’s unconditional abatement as its flat—or direct—contribution, to its conditional abatement as its indirect contribution, and to the matching factors as matching rates. We have chosen our terminology in the hope that it is more transparent.) Assuming countries can commit to the matching factors they declare (see the section “Commitment” for a justification of this assumption), this program allows countries to alter the incentives of other countries without invoking a central authority.

Boadway *et al*’s^21^ inspirational analysis considers an arbitrary number of heterogeneous countries and arrives at an optimistic result. They show that a matching-commitment agreement can lead to new equilibrium emission levels that are locally Pareto efficient and unique and reduce emissions relative to the status quo. However, Boadway *et al*. consider the status quo emissions in the absence of an agreement to be exogenously determined, and their results require that at least one country has an incentive to unilaterally reduce emissions from this status quo even in the absence of an agreement. A more realistic scenario would be that countries follow their Nash equilibrium emissions strategy in the absence of an agreement, so that no country has an incentive to either increase or decrease their emissions^6,23^. In that case, Boadway *et al.*’s optimistic results disappear. In other words, in Boadway *et al*.’s analysis, without an agreement, at least one country’s emissions are irrationally high, even for its pure self-interest; matching-commitment agreements reduce emissions because they allow countries to respond to their incentives and thus lower their emissions. Thus, we do not know whether matching-commitment agreements can work to reduce emissions if countries are able to freely select emissions levels that align with their incentives in the absence of an agreement. Furthermore, Boadway *et al*. do not consider the question of global efficiency, i.e., whether the agreement improves global welfare, the payoff of all countries, or indeed any single country’s payoff, relative to the outcome in the absence of an agreement (i.e., either relative to a Nash equilibrium emissions profile, or to the non-Nash baseline Boadway *et al*. assume is selected in this case).

Against this background, this paper addresses two main questions: First, can a matching-commitment agreement incentivize countries to reduce their emissions relative to a baseline scenario without an agreement, and in which countries’ choices align with their incentives? Second, are countries better off with such an agreement than without? We build on Boadway *et al*.’s^21^ formulation, but require the baseline scenario—that is, countries’ emissions in the absence of an agreement for reducing them—to be a Nash equilibrium determined endogenously by the countries’ strategic decisions. For arbitrarily many countries that may differ in their economic and climate-related properties, and that choose their emissions independently, we find conditions guaranteeing that such a Nash equilibrium emissions profile exists and is unique—i.e., the baseline scenario is well-defined. Next, we analyze the effects of a matching-commitment agreement on the emissions of two such countries. We show that a matching-commitment agreement generates a new, unique equilibrium emissions profile. (More precisely, there are multiple subgame-perfect Nash equilibria—see “Model and methods” and ref. ^24^—which all yield identical emissions profiles.) At this equilibrium emissions profile, both countries’ emissions are lower and their payoffs are higher than at baseline, i.e., at the equilibrium without a matching-commitment agreement. Lastly, we show that the equilibrium emissions profile with matching-commitment agreements is locally Pareto efficient.

While Boadway *et al*.’s^21^ results broke new ground and motivated all that we have done, there are subtle problems with their arguments for both the existence and the uniqueness of the equilibrium, and hence the claims for the general case do not hold up (see Remark B.15 and Appendix C.11.6 for details). Indeed, even the two-country case is challenging for providing a rigorous proof of existence and uniqueness, which requires a different mathematical approach than that of Boadway *et al*. We introduce that method and proof in this paper for matching-commitment agreements between two countries, and show rigorously that the equilibrium (Eqs. (5) and (6)) was, in fact, correctly identified by Boadway *et al*. The *n*-country case remains an open problem.

## Model and methods

### Basic climate game (BCG)

We start by describing a model for the public goods problem of global GHG emissions between two countries (or unions thereof, e.g., the European Union), building on Boadway *et al*.’s^21^ model, which in turn is similar to one described by Finus (ref. ^22^, Ch. 9) and an example of Folmer and von Mouche’s^25^ transboundary pollution games. As is often the case for game-theoretical models of international cooperation, we focus on two countries, to retain mathematical tractability (see, e.g., refs. ^26–32^; we compare our analysis with the literature on *n*-player matching mechanisms in the section “Two vs. many countries”).

We consider two countries deciding on their emissions levels, ${e}_{i}\in {\mathbb{R}}\,(i=1,2)$. We denote the **emissions profile**, i.e., the vector of countries’ emissions, by ${\boldsymbol{e}}=({e}_{1},{e}_{2})\in {{\mathbb{R}}}^{2}$, and the **total emissions** by $e={e}_{1}+{e}_{2}$. Note that we allow countries to have negative net GHG emissions (e.g., by capturing carbon from the atmosphere), since this is assumed in many theoretical mitigation scenarios that meet the goals of the Paris Agreement^33^. We denote by ${B}_{i}({e}_{i})$ and ${D}_{i}(e)$ the benefit and damage, respectively, to country *i* (*i* = 1, 2) when its own emissions level is *e*_*i*_ and the total emissions are $e={e}_{1}+{e}_{2}$. The benefit and damage functions are assumed to be increasing and twice differentiable, as well as decelerating and accelerating, respectively (see “Increasing damages from climate change”). Country *i*’s total payoff as a function of the emissions profile ***e*** is

1 $${\prod }_{i}({\boldsymbol{e}})={B}_{i}({e}_{i})-{D}_{i}(e)\mathrm{}.$$

Countries seek to maximize their own payoffs, and are indifferent to the other country’s payoff.

In our baseline scenario, which we call the **basic climate game (BCG)**, representing the absence of any international agreement to curb emissions, countries independently and simultaneously choose their own emissions levels. When countries play the BCG and there is a unique Nash equilibrium, we view it as the baseline scenario, as it represents the predicted emissions profile for rational countries in the absence of any agreement to limit emissions. Unfortunately, and not surprisingly, the Nash equilibrium of the BCG does not maximize **global welfare**, i.e., the total payoff of both countries, ∏ = ∏_1_ + ∏_2_, and is hence not socially optimal. Indeed, we show in Appendix A.3 that the total emissions at a **social optimum (SO)**, i.e., at an emissions profile that maximizes global welfare, must be lower than the total baseline emissions (Appendix A.3 discusses when an SO exists, which is in general not guaranteed by our assumptions). Moreover, since the baseline emissions profile is not Pareto-efficient, there are emissions profiles at which both countries’ payoffs are higher than at baseline (Appendix A.4). Thus, an international environmental agreement that results in lower emissions and higher national payoffs than baseline is desirable (but is still likely to be second-best from a global perspective).

### Matching climate game (MCG)

One suggested approach to establishing an accord that incentivizes a reduction in GHG emissions is to use a matching-commitment scheme^17,21^. In this approach, country *i* may reduce its emissions relative to its baseline emissions level ${\bar{e}}_{i}$ by an abatement level *A*_*i*_ ≥  (*i* = 1, 2), so that its actual emissions level is

2 $${e}_{i}={\bar{e}}_{i}-{A}_{i}\mathrm{}.$$

Country *i*’s abatement *A*_*i*_ is the sum of its unconditional abatement *a*_*i*_, and the conditional abatement that it performs in response to the other country’s unconditional abatement,

3 $${A}_{i}={a}_{i}+{m}_{i}{a}_{j},$$

where *m*_*i*_ is the proportionality factor at which country *i* matches country *j*’s unconditional abatement and *i*, *j* = 1, 2, *i* ≠ *j* (that is, *j* is the *nonfocal* country). The countries then play the following game, henceforth referred to as the **matching climate game (MCG)** (see also Definition A.9):

- Stage I: Countries independently and simultaneously choose their (non-negative) matching factors, *m*_*i*_ ≥ 0, to which they are subsequently committed.
- Stage II: Countries independently and simultaneously choose their unconditional abatement levels, *a*_*i*_, with full knowledge of the matching factors chosen in stage I.

Denoting the total baseline emissions and the total abatement by $\bar{e}={\bar{e}}_{1}+{\bar{e}}_{2}$ and $A=\bar{e}-e$, respectively, country *i*’s payoff becomes the nonlinear function

4 $${\prod }_{i}({\boldsymbol{e}})={B}_{i}({\bar{e}}_{i}-{A}_{i})-{D}_{i}(\bar{e}-A)\,{\rm{for}}\,i=1,2.$$

Note that the baseline emissions levels ${\bar{e}}_{1}$ and ${\bar{e}}_{2}$ must be specified to determine the country payoffs in the MCG. Below, we will take these to be the Nash equilibrium emissions of the BCG (which, under the conditions described in the section “Assumptions on the costs and benefits of emissions”, exists and is unique). These baseline emissions are thus endogenously determined in the sense that they are the rational levels of emissions in the absence of any climate agreement.

**Figure 1:**
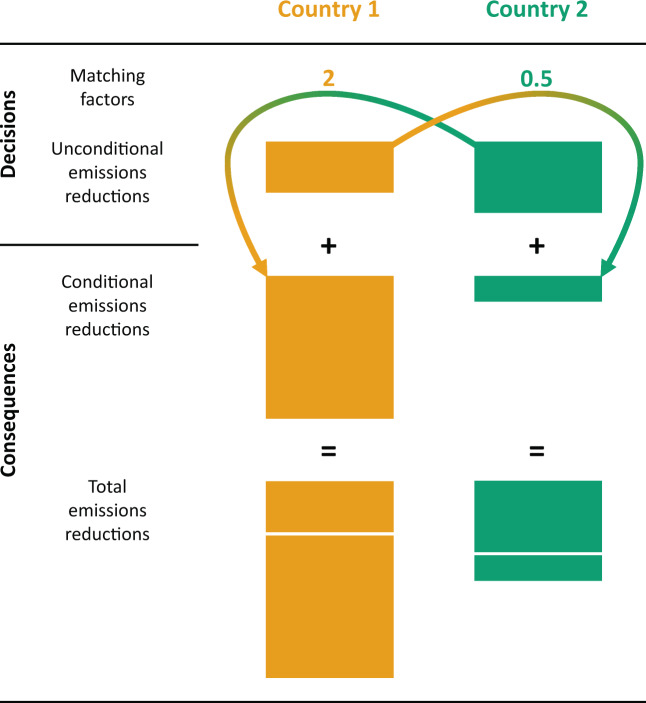
Graphical representation of the matching climate game (MCG). In stage I, countries decide on their matching factors. In stage II, countries choose their unconditional abatements, taking into consideration the matching factors chosen in stage I. These decisions then determine the conditional abatements countries perform over and above their unconditional abatements: each country’s conditional emissions reduction is the product of its own matching factor, and the *other* country’s unconditional abatement.

**Figure 2:**
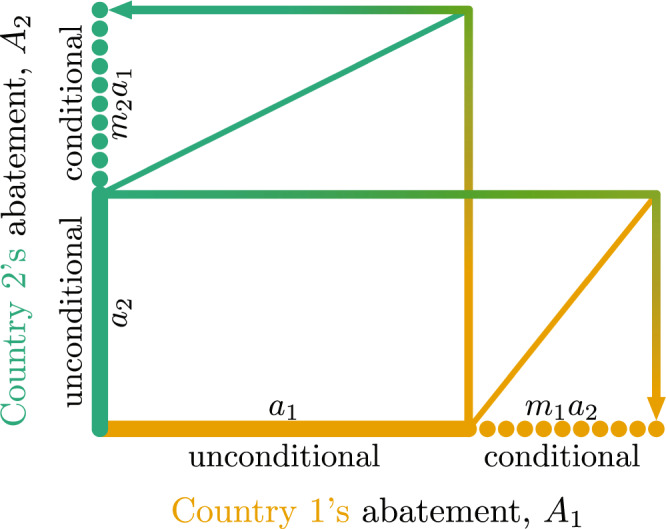
Graphical representation of how two countries’ decisions on their matching factors *m*_*i*_ and unconditional abatements *a*_*i*_ determine their total abatements *A*_*i*_ in the matching climate game (MCG). The two countries’ decisions are shown as thick continuous lines, while the consequences of these decisions are shown as thick dotted lines, with thin continuous lines serving as visual aids. Each country’s total abatement, indicated for countries 1 and 2 by the lengths of the thick horizontal and vertical lines, respectively, is partitioned into two components: an unconditional abatement and a conditional abatement. In stage I, each country chooses its matching factor, indicated by the slopes of the two diagonal lines: the ratio of country *i*’s conditional abatement and country *j*’s (*j* ≠ *i*) unconditional abatement is country *i*’s matching factor *m*_*i*_, so the slopes of the top-left and bottom-right diagonal lines are *m*_2_ and 1/*m*_1_, respectively. In stage II, each country chooses its unconditional abatement (*a*_*i*_), indicated by the lengths of the horizontal and vertical segments of the thick continuous lines. These decisions then determine each country’s conditional abatement, indicated by the lengths of the dotted lines. The lines with graduated colours represent how one country’s unconditional abatement is matched by the other country.

The process by which national abatements arise from the unconditional abatements and matching factors is visualized in Figs. 1 and 2.

### Assumptions on the costs and benefits of emissions

In this paper, we aim to compare the equilibrium outcomes of the basic climate game (BCG) and the matching climate game (MCG). Since such a comparison is predicated on the existence of well-defined equilibrium outcomes for these two games, additional assumptions are needed to guarantee this. For example, in the BCG, it may be advantageous for countries to emit or abate without bound, in which case no Nash equilibrium exists. In the MCG, matching may incentivize a country to abate without bound, in which case the best-response functions for stage II (given matching factors chosen in stage I) need not even be well-defined.

In Appendix A.1, we identify a sufficient condition on the benefit and damage functions that guarantees the existence of a unique Nash equilibrium for the BCG; we call this condition **bounded global emissions (BGE)** (Definition A.2), because, roughly, it ensures that all rational emissions profiles lie inside a bounded set. In Appendix A.6, we show that under the assumptions of the BCG, the MCG’s stage-II best-response functions are well-defined if and only if (iff) for any country *i*, in the limit of negative infinite emissions, either the marginal benefits become infinite or the marginal damages vanish; we refer to this condition as **bounded abatement with matching (BAM)**, because it guarantees that no matching factors can incentivize infinite abatement. Henceforth we assume the benefit and damage functions are such that BAM holds and that global emissions are bounded. It turns out that (at least for the case of two countries) the BAM condition is sufficient to ensure a unique equilibrium outcome for the MCG, in the sense described in our “Results” section. (Note that Boadway *et al*.^21^ do not assume the BAM condition, and their analysis does not address the possibility of the MCG’s stage-II best-response functions being undefined. Consequently, the MCG’s stage-II Nash equilibria in their analysis may not exist.)

While the model described above and the results that follow involve two countries only, note that for any number of countries, *n* ≥ 2, the BGE and BAM assumptions (resp.) ensure the existence and uniqueness of a Nash equilibrium for the BCG, and that the MCG’s stage-II best-response functions are well-defined. Regardless of the number of countries, these properties are necessary to sensibly ask whether and when the MCG yields equilibria that improve on the baseline emissions profile.

### Solution concepts

The relevant solution concept for the basic climate game (BCG) is the Nash equilibrium, i.e., an emissions profile from which no country has an incentive to deviate unilaterally (see ref. ^24^, Ch. 4). The matching climate game (MCG), on the other hand, is a two-stage game in which a strategy for country *i* (*i* = 1, 2) specifies its choice of a matching factor, *m*_*i*_ = *μ*_*i*_ in stage I, and of an unconditional abatement level for any combination of matching factors chosen by both countries in stage II, *a*_*i*_ (*m*_1_, *m*_2_) (*i* = 1, 2). A natural choice of solution concept for the MCG is therefore the **subgame-perfect equilibrium** (**SPE**; see Ch. 7 of ref. ^24^), which yields Nash equilibria at every subgame (the MCG has infinitely many subgames: in addition to the MCG itself, for any choice of matching factors in stage I, the game played in stage II is a subgame of the MCG). For the MCG, an SPE must then satisfy two conditions: first, it must constitute a Nash equilibrium for the full MCG, and second, for any pair of matching factors (*m*_1_, *m*_2_), the abatements (*a*_1_ (*m*_1_, *m*_2_), *a*_2_ (*m*_1_, *m*_2_)) must constitute a Nash equilibrium of stage II. For any choice of matching factors played in stage I, an SPE selects abatements that are Nash equilibria for stage II, so this solution concept avoids Nash equilibria (for the full MCG game) that are sustained by incredible threats or other irrational behaviour in stage II. We focus on SPEs precisely because they avoid such irrational behaviour, and our main aim is to identify and compare SPEs of the MCG with the baseline emissions profile.

## Results

### The matching climate game has a unique equilibrium emissions profile

We first use backward induction to find subgame-perfect equilibria (SPEs) for the matching climate game (MCG; see the “Model and methods” section and Definition A.9) between two countries: We assume that given any choice of matching factors in stage I, countries may expect one another to choose unconditional abatement levels that are Nash equilibria given their choices of matching factors, (*m*_1_, *m*_2_); we then find which strategies will be played in stage I. A more detailed account of the process follows below, and the full details are given in Appendix B.

**Figure 3:**
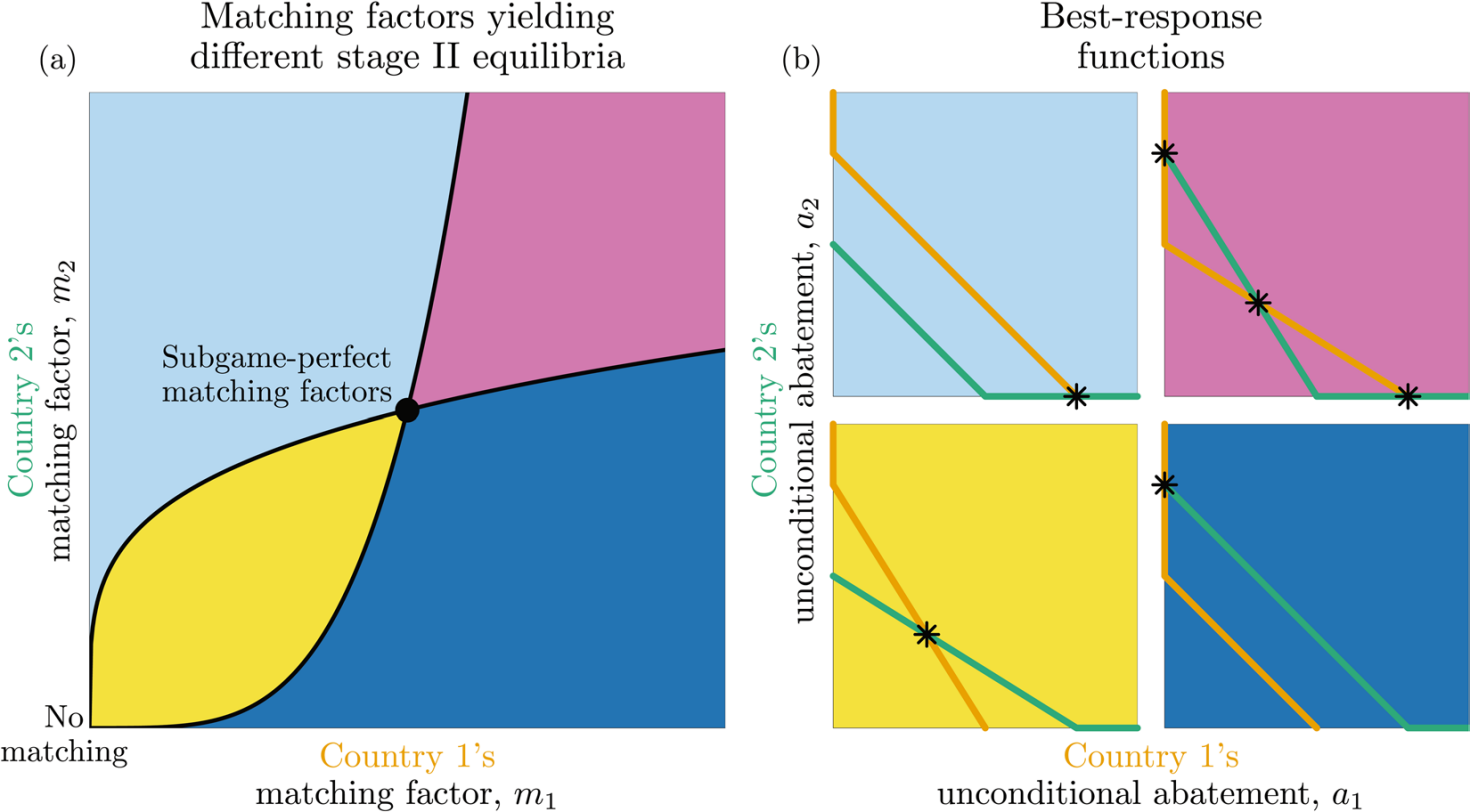
(**a**) Schematic overview of outcomes in stage II of the matching climate game (MCG) for pairs of matching factors chosen in stage I; different colours correspond to qualitatively different stage-II Nash equilibria. The coloured areas are separated by the stage-II delimiter curves (*ϕ*_1_ and *ϕ*_2_, shown as the concave and convex black curves, respectively), and the filled circle marks the pair of matching factors yielding equilibria. (**b**) Each panel shows a qualitatively different configuration of the best-response functions for pairs of matching factors in the correspondingly coloured area in panel (a), with asterisks indicating stage-II Nash equilibria. When the subgame-perfect matching factors are played in stage I, the stage-II best-response functions overlap for positive unconditional abatements. Note that, in general, the best-response functions need not be piecewise linear.

In Appendix B.1, we find the Nash equilibrium levels of unconditional abatement in stage II, given a choice of matching factors in stage I, which can be geometrically characterized as intersections of the stage-II best-response functions, ${ {\mathcal R} }_{1}\,{\rm{and}}\,{ {\mathcal R} }_{2}$. The results of our analysis are summarized in Fig. 3. In particular, we find that there are four regions in the set of non-negative matching factors, in which the stage-II Nash equilibria are qualitatively different. These regions are delimited by the two **stage-II delimiter curves**, *ϕ*_1_ and *ϕ*_2_ (shown in Fig. 3a as the concave and convex curves, respectively), defined as follows: for any ${m}_{j}\ge 0$, ${m}_{i}={\phi }_{i}({m}_{j})$ is the matching factor at which the intercepts of the two countries’ stage-II best-response functions with the *a*_*i*_-axis are identical (Appendix C.7). Between *ϕ*_*i*_ and the *a*_*j*_-axis, country *i*’s stage-II best-response curve intercepts the *a*_*i*_-axis higher than does *j*’s.

The functions *ϕ*_1_ and *ϕ*_2_ intersect at two pairs of matching factors: (1) *m*_1_ = *m*_2_ = 0, for which the unique stage-II Nash equilibrium is no abatement (baseline), and (2) a pair of matching factors $({m}_{1}^{{\rm{c}}},\,{m}_{2}^{{\rm{c}}})$ that are reciprocal (satisfy ${m}_{1}^{{\rm{c}}},\,{m}_{2}^{{\rm{c}}}=1$; since our model is a one-shot game, there is no future time at which countries have occasion to reciprocate in the sense of responding to one another’s actions), defined by Eqs. (5) and (6) below, for which the best-response functions overlap for positive unconditional abatements, yielding a continuum of stage-II Nash equilibria.

Furthermore, we find that for pairs of matching factors other than $({m}_{1}^{{\rm{c}}},\,{m}_{2}^{{\rm{c}}})$, the stage-II best-response functions cross at most once in the interior of the quadrant of positive unconditional abatements, i.e., $\{({a}_{1},\,{a}_{2})|{a}_{1} > 0,\,{a}_{2} > 0\}$. This implies that for $({m}_{1},\,{m}_{2})\ne ({m}_{1}^{{\rm{c}}},\,{m}_{2}^{{\rm{c}}})$ either one best-response function is above the other (upper-left and lower-right panels of Fig. 3b), or they cross at some point in the interior of that quadrant (upper-right and lower-left panels of Fig. 3b). Consequently, there are either one, two, or three stage-II Nash equilibria (Fig. 3b), and these cases can be distinguished by comparing the stage-II best-response functions’ intercepts with the *a*_1_- and *a*_2_-axes:

- On or between *ϕ*_1_ (resp. *ϕ*_2_) and the *a*_2_-axis (*a*_1_-axis), there is a stage-II Nash equilibrium at which only country 1 (2) abates unconditionally.
- Between the curves *ϕ*_1_ and *ϕ*_2_, there is a stage-II Nash equilibrium at which both countries abate unconditionally.

Next, for any pair of non-negative matching factors, $({m}_{1},\,{m}_{2})\in {{\mathbb{R}}}_{\ge 0}^{2}$, the payoffs ∏_*i*_(*m*_1_, *m*_2_) (*i* = 1, 2) can be determined assuming that a Nash equilibrium is played in stage II; we ascribe these payoffs to pairs of matching factors and obtain payoff functions ∏_*i*_(*m*_1_, *m*_2_) that we then use to find matching factors that are Nash equilibria of stage I (assuming Nash equilibrium unconditional abatements are played in stage II). These Nash equilibrium matching factors, along with the choice of Nash equilibria played in stage II that was used to calculate the stage I payoff functions, constitute SPEs.

However, the multiplicity of stage-II Nash equilibria for some pairs of matching factors introduces a complication, because the payoff to country *i* given (*m*_1_, *m*_2_) may then not be uniquely defined even under the assumption that only Nash equilibria are played in stage II (see top-right corner of Fig. 3b); one must then analyze an infinite family of continuous-strategy games, each with payoff functions ∏_*i*_(*m*_1_, *m*_2_) (*i* = 1, 2) determined by a particular choice of stage-II equilibria (for all pairs of matching factors for which multiple stage-II equilibria exist).

Despite this complication, it turns out that there is a unique SPE emissions profile. In particular, analyzing all possible pairs of payoff functions ∏_*i*_(*m*_1_, *m*_2_) (*i* = 1, 2) obtained assuming equilibrium stage-II play (see Theorem B.19 in Appendix B.2), we show that although the MCG has infinitely many SPEs, the unique pair of matching factors $({m}_{1}^{{\rm{c}}},\,{m}_{2}^{{\rm{c}}})$ is played at all SPEs. Moreover, the equilibrium abatements *A*_*i*_ (and, therefore the payoffs ∏_*i*_) for the MCG are uniquely defined in the sense that they do not depend on which SPE is chosen. Specifically, with *m* and *a* denoting the unique positive solutions of

5 $${B{\prime} }_{1}({\bar{e}}_{1}-a)=(1+m){D{\prime} }_{1}(\bar{e}-(1+m)a),$$

6 $$m{B{\prime} }_{2}({\bar{e}}_{2}-ma)=(1+m){D{\prime} }_{2}(\bar{e}-(1+m)a),$$

the equilibrium matching factors are $({m}_{i}^{{\rm{c}}},\,{m}_{j}^{{\rm{c}}})=(1/m,\,m)$, and the emissions profile at any SPE is

6 $$({e}_{1},\,{e}_{2})=({\bar{e}}_{1}-a,\,{\bar{e}}_{2}-ma).$$

### Both countries are better off playing the matching climate game than the basic climate game

Comparing the subgame-perfect emissions profile of the matching climate game (MCG) given by Eq. (7) with the baseline emissions profile ($\bar{e}$, the Nash equilibrium of the basic climate game, abbreviated BCG), we find:

- At the equilibrium emissions profile of the MCG, both countries’ emissions are lower, but their payoffs are higher, than at the baseline equilibrium (i.e., the subgame-perfect emissions profile is a Pareto improvement on the Nash equilibrium of the BCG).
- The equilibrium emissions profile of the MCG is locally Pareto efficient, i.e., a small deviation from the MCG’s equilibrium abatement levels cannot increase both countries’ payoffs.
- The MCG’s equilibrium emissions profile is socially optimal iff the two countries’ marginal benefits at the equilibrium emissions profile are equal, which need not generally be the case; it then follows that from the perspective of a social planner, the country having lower marginal benefits at the subgame-perfect emissions profile emits too much, while the one with higher marginal benefits emits too little (Lemma A.8).

## Discussion

Matching-commitment agreements have been garnering increasing interest as a way to construct international agreements to lower global greenhouse gas (GHG) emissions without requiring extensive international monitoring and enforcement. Our main contribution in this paper is to show the theoretical viability of such an approach assuming that countries behave strategically in the absence of an agreement. We show that matching-commitment agreements can indeed result in increased abatement and welfare for the countries involved, relative to a baseline of strategic behavior in the absence of agreements. Our results suggest that matching-commitment agreements can be a promising option for addressing other public goods problems when enforcement is difficult.

To conclude, in the next few subsections we position our results in a broader context. First, we highlight some appealing features of the matching climate game (MCG). Next, we touch on related models from the literature on matching-commitment agreements. We then discuss the main simplifying assumptions made in our model, and mention how our assumptions regarding the damage and benefit functions could be relaxed. Lastly, we discuss how our methods can be applied to the provision of public goods in general.

### Advantages over other climate-agreement schemes

In addition to improving on the baseline scenario, the matching climate game (MCG) has a number of attractive features in comparison with other mechanisms (including other matching mechanisms) suggested to address international public goods problems such as reducing global GHG emissions, which we describe below.

The MCG asks countries to relinquish very little of their sovereignty and entails no negotiation. First, a country that agrees to participate in the MCG may still choose not to match, and hence does not commit to any policy change, other than refraining from increasing its emissions in response to other countries’ emissions reductions, a phenomenon known as carbon leakage. (Similar to Barrett’s^9^ approach, including a clause stipulating that the matching agreement does not come into force unless all countries are signatories can help assuage countries’ fears that their environmental policies would be undone by defecting countries, and hence stabilize participation in the treaty and avoid leakage.) Second, unlike many other climate-agreement schemes (e.g., refs. ^8,13^), signatories are not asked to expose themselves to the possibility of economic sanctions. Third, countries decide on their own policies for matching factors and unconditional abatements, rather than submitting to the authority of an institution or coalition of countries (see, e.g., ref. ^34^ for such a model without matching; see refs. ^35,36^, respectively, for matching mechanisms in which a coalition of signatories decides on a common matching factor at which all members will match the total unconditional abatement of all countries, or of other coalition members). Fourth, countries are only asked to commit to conditional actions (as opposed to the Paris Agreement’s pledges), and to refrain from emitting more than they would in the absence of an agreement. In other words, the only encroachments on national sovereignty that the MCG makes is requiring countries to keep their word and fulfill a conditional commitment that they choose to make.

The fact that the MCG does not employ costly punishments is also appealing, because it sidesteps the second-order free-riding problem: when punishment is costly, players are tempted to let others do the punishing^37,38^. While in the MCG countries can implicitly punish other countries for insufficiently high matching commitments by reducing their unconditional abatements, at the subgame-perfect emissions profile doing so is not costly in the sense that the countries choose Nash equilibrium unconditional abatements (given the matching factors chosen in stage I).

In an analysis of a similar matching-commitment agreement between *n* identical countries, Rübbelke^39^ points out that, because countries commit only to matching factors, they can still adjust their unconditional abatements if they discover (after having made their matching commitments) that they underestimated the benefits of abatement. This flexibility allows countries to at least partially (and sometimes fully) correct their emissions abatements based on updated estimates of benefits, avoiding the need for costly renegotiations. Although we do not repeat Rübbelke’s analysis for our model, the flexibility in the choice of unconditional abatements in the MCG is likely to give rise to a similar ability to compensate for updated estimates of the benefits of abatement.

Lastly, many suggestions for climate agreements involve international monetary (or in-kind) transfers through which countries pay to subsidize the abatement efforts of other countries^13,26,34,40–42^. The MCG similarly subsidizes abatements in that positive matching factors effectively reduce the marginal costs of abatements. However, in the MCG, countries’ subsidies are implemented not by transferring resources, but through additional abatements provided conditionally. This type of subsidy is essentially unilateral and seems more likely to be implemented than one that involves monetary or in-kind transfers, especially in the case of countries between which diplomatic relations are tense or nonexistent.

### Related literature

To our knowledge, Rübbelke’s^39^ was the first to analyze a matching-commitment agreement to reduce GHG emissions, in which each country declares a single matching factor at which it will match any other country’s abatement. As is common in the literature on matching-commitment agreements, his analysis is restricted to identical countries and symmetric equilibria, whereas we allow for heterogeneous countries and asymmetric equilibria. The style of our model is also different from Rübbelke’s^39^ in that—similarly to Boadway *et al*.^21^—we use Finus’s^22^ well-established emissions game to specify payoffs from emissions profiles (ref. ^39^ instead builds on the standard model of public goods, see, e.g., ref. ^43^). Our analysis is closest in spirit to Boadway *et al*.’*s*^21^, with the most important difference being this: Boadway *et al*.’*s* main result concerns the efficacy of the matching climate game (MCG) in emissions reductions relative to an exogenously-determined baseline scenario in which at least one country would benefit from unilaterally reducing its emissions—in which case emissions would be reduced even without an agreement, if this country simply followed its incentives. By contrast, our main contribution here is to show that the MCG incentivizes countries to lower emissions relative to an endogenously-determined baseline scenario from which they have no incentive to deviate.

Matching mechanisms are most commonly studied in the general context of public goods or externality problems, and in that context our analysis is closest to Guttman and Schnytzer^44^. However, those authors (a) show that a subgame-perfect equilibrium (SPE) exists for positive externalities, and show that their approach may not work for negative externalities (such as GHG emissions, analyzed here), and (b) do not establish that matching commitments yield unique equilibrium actions (i.e., an emissions profile in a climate agreement), whereas we are able to do so. Note also that in the context of public goods provision, Buchholz *et al*.^45,46^ have shown that when players are heterogeneous, matching often leads to inefficient equilibria at which not all players contribute. Our analysis shows that this problem is avoided in our approach, regardless of any possible inequalities between the countries.

Lastly, we mention that another growing branch of the research into social dilemmas in general, and climate governance in particular, involves the application of methods from statistical physics (see ref. ^47^ for a review, or ref. ^48^ for a notable example). Broadly, this literature takes a dynamic, evolutionary game-theoretic perspective, in which the independent agents (e.g., people, but also possibly countries) interact many times, and over time alter their strategies based on the payoffs they and others receive (but do not strategically analyze the game they are facing). To our knowledge, these methods have not yet been applied to matching-commitment agreements, and are a promising avenue for circumventing the technical difficulty of analyzing matching-commitment agreements among many countries in the “classical” game-theoretic setting.

### Main simplifying assumptions

Our analysis makes use of a number of simplifying assumptions about climate agreements, the most important of which are discussed below.

#### One-shot game

Similar to, e.g., ref. ^49^, our simple formulation of the choice of GHG emissions levels in a one-shot game forces countries to make all environmental policy decisions at one point in time. It thus removes the possibility of countries responding to either observed environmental changes or one another’s behaviour. In Barrett’s^49^ words, it “can be interpreted as compressing perhaps a century of decision-making into a single period,” and the damage and benefit in question “should be interpreted as capturing the full consequences, including into the distant future, of decisions taken this century”. Some of the unforeseen environmental consequences of these emissions will only be experienced in the far future, so no observations of such consequences are available to affect policy in the near future. However, the assumption that countries cannot observe and respond to each other’s behaviour is more restrictive. Even if the full consequences of emissions are not immediately apparent, countries can still assess other countries’ emissions, and would likely vary their own emissions in response. We therefore regard this model as a first step and proof-of-concept for the use of matching-commitment agreements to lower GHG emissions.

There is, however, some experimental evidence that matching-commitment agreements can increase the provision of public goods. In particular, Guttman^50^ and Bracht *et al*.^51^ observe that matching-commitment mechanisms similar to that employed in our matching climate game (MCG) yield significant improvements in public good provision in repeated public goods games played in groups, and between pairs, respectively (see refs. ^52,53^ for reviews of the experimental literature on matching).

#### Commitment

Because our model is a one-shot game, it only addresses the issue of commitment in a limited fashion: even if country 2 could renege on the matching factor it committed to in stage I, it has no incentive to do so, and in particular, it does better playing a cooperative subgame-perfect equilibrium (SPE) than it does by reverting to its baseline emissions, i.e., choosing *m*_2_ = 0 and *a*_2_ = 0. This is because, even if country 1 continues to play ${m}_{1}^{{\rm{c}}}$, it can still respond to country 2’s defection by decreasing its unconditional abatement, ${a}_{1}({m}_{1}^{{\rm{c}}},{m}_{2})$. But, if both countries are free to renege on their matching commitments, stage I is merely “cheap talk” and the original basic climate game (BCG; Definition A.1)—in which the baseline emissions profile is the only equilibrium—is recovered.

In the language of Barrett^14^, the ability of countries to commit assumes the “enforcement problem” is solved. As Barrett^49^ notes, this is justified by the international-law custom of treaties being binding, *pacta sunt servanda*. This custom is codified in the Vienna Convention on the Law of Treaties: “Every treaty in force is binding upon the parties to it and must be performed by them in good faith” (ref. ^54^, p. 339). Thus, if countries can agree that whatever matching commitments they themselves select be *legally binding*, they are more likely to keep their word. Since countries are also free to refrain from any matching (i.e., matching factors may be zero), and since the matching commitments are conditional on other countries’ actions, such commitments are weaker—and hence safer from being exploited, and thus more likely to be kept—than commitments to specific emissions abatements.

Two recent examples suggest that legally binding commitments to match emissions reductions would likely be an improvement on the status quo, even without an explicit enforcement mechanism in place. First, some parties to both the Kyoto Protocol and the Paris Agreement rejected legally binding targets^55,56^; this suggests that countries ascribe more weight to commitments that are explicitly legally binding, possibly due to the risk of reputation loss^57^. Second, although the Paris Agreement’s emissions targets are not binding, other aspects of the agreement are, and even the withdrawal of the US from the Paris Agreement will conform to the agreement’s rules for withdrawal^58^.

#### Two *vs*. many countries

While it is widely acknowledged that an effective agreement must include as parties the two largest emitters, the US and China, their combined emissions amount to about 45% of global emissions, so a climate agreement between only these countries will likely be insufficient^3^. We leave the analysis of matching-commitment agreements among many countries for future work, but note that a number of studies suggest that such agreements among many countries can also be effective in reducing emissions. For instance, encouraging analytical results include those of Fujita^35^ and Rübbelke^39^ (described in the sections “Advantages over other climate-agreement schemes” and “Related literature”). Using the STACO model^40^ to estimate countries’ damages and benefits from emissions, Kawamata and Horita^59^ simulate a matching scheme similar to the matching climate game (MCG) for six countries and regions and show that emissions are substantially reduced. Lastly, in Guttman’s^50^ experimental study, matching commitments also increased public good provision in repeated games in groups of 3–6 players.

In the context of multilateral matching-commitment agreements, it will likely be imperative that *all* countries be signatories. This is because although matching-commitment agreements do not strictly require signatories to perform any abatement, they do require that countries refrain from emitting more than they would in the absence of an agreement. If some countries opt out of the agreement, and signatories collectively abate their total emissions, then non-signatories will have an incentive to increase their emissions from their no-agreement baseline emissions (this is the carbon leakage mentioned in the section “Advantages over other climate-agreement schemes”).

#### Increasing damages from climate change

Ricke *et al*.^60^ recently estimated the country-level social cost of carbon (CSCC)—a measure of the long-run economic cost of a tonne of CO_2_ emitted in 2020. The CSCC is analogous to the marginal damage suffered by each country, ${D{\prime} }_{i}$: it quantifies the marginal cost of emissions by comparing a reference emissions pathway (describing how emissions are distributed over a length of time) to a pathway that begins with an *additional* pulse of one tonne of CO_2_ emissions, but is otherwise identical to the reference pathway. Ricke *et al*.^60^ repeat these calculations for a variety of different socioeconomic scenarios (that result in different emissions pathways) and for different empirical estimates of the economic impacts of warming. (Note that warming, and consequently damage due to climate change, does not depend strongly on the emissions pathway and is well described by the cumulative emissions^61,62^. Thus, our formulation assumes that damages result from global cumulative emissions over a long time-horizon, ignoring the possible impact of the emissions pathway).

While one of Ricke *et al*.’*s*^60^ model specifications results in a negative CSCC (i.e., a climate *benefit*) for some countries, they note that “[t]hese results are among the most sensitive in the analysis, as under the BHM long-run and DJO damage model specifications all countries have positive CSCC”, where BHM and DJO refer to the damage-function estimates of Burke *et al*.^63^ and Dell *et al*.^64^, respectively. Moreover, they find that “under the long-run impact model specifications [...] countries like Russia, Canada, Germany and France that have negative CSCC under the reference case switch to having among the highest positive CSCCs”. Another analysis by Kompas *et al*.^65^ applies a “computable general equilibrium” approach and finds that all of the 139 countries included in their analysis benefit from complying with the Paris Agreement, providing additional support for our assumption that the marginal damages of emissions are positive.

### Relaxation of assumptions on the damages and benefits of emissions

The assumption that global emissions are bounded (Definition A.2) can be relaxed: its function is to ensure that the basic climate game (BCG) has a unique Nash equilibrium, but all that is required for our results to hold is that the BCG has a Nash equilibrium, and that it is chosen as the baseline emissions profile. In contrast, the assumption of bounded abatement with matching (BAM) is both necessary and sufficient for the matching climate game’s (MCG) stage-II best-response functions to be well-defined, and hence cannot be relaxed.

### Application to the provision of general public goods

While the matching climate game (MCG) and our analysis are framed in terms of climate agreements, they can be applied equally well to other public goods problems. The public good provided in our model is the *abatement* of GHG emissions, and so reduced emissions correspond to increased abatement. This in turn implies that our assumption that the benefits and damages of emissions are decelerating and accelerating, respectively, translate into requiring that the costs and benefits of providing the public good being accelerating and decelerating, respectively. Our analysis then shows that the MCG may also be useful in promoting the provision of public goods satisfying these assumptions, in situations in which enforcing cooperation is difficult^46,53,66^.

## Supplementary information

Electronic supplementary material.
