# Supplementary material for: Combating climate change with matching-commitment agreements
Source: Sci Rep. 2020 Jun 19;10:10251. doi: 10.1038/s41598-020-63446-1 (PMC7305217; doi:10.1038/s41598-020-63446-1)
Supplement: Supplementary file 1 — Electronic supplementary material. [file 41598_2020_63446_MOESM1_ESM.pdf]

Combating climate change with matching-commitment agreements: Appendices

Chai Molina*[1,2,3], Erol Akçay†[3], Ulf Dieckmann†[1], Simon A. Levin†[1,2,4,5], and Elena A. Rovenskaya†[1,6]

[1]International Institute for Applied Systems Analysis, Laxenburg, A-2361, Austria
[2]Department of Ecology and Evolutionary Biology, Princeton University, Princeton 08544 NJ, USA
[3]Department of Biology, University of Pennsylvania, Philadelphia, PA, 19104, USA
[4]Beijer Institute of Ecological Economics, SE-104 05 Stockholm, Sweden
[5]Resources for the Future, Washington, DC 20036, USA
[6]Faculty of Computational Mathematics and Cybernetics, Lomonosov Moscow State University, Leninskiye Gory 1-52, Moscow 119234, Russia

April 23, 2020 @ 18:28

*Corresponding author; electronic address: chai.molina@gmail.com; telephone number: +1 (609) 258-7437.

†These authors contributed equally to this study and are listed alphabetically.

Main text DOI: 10.1038/s41598-020-63446-1.

Contents

A Model setup **3**
- A.1 Assumptions, notation and terminology . . . 3
- A.2 The baseline emissions levels . . . 5
- A.3 The social optimum . . . 6
- A.4 Characterization of local Pareto efficiency . . . 7
- A.5 Comparison of Pareto-efficient and socially optimal emissions profiles . . . 9
- A.6 The Matching Climate Game . . . 10

B Analysis of the matching climate game for two countries **13**
- B.1 Stage-II equilibria: choosing unconditional abatement levels . . . 13
  - B.1.1 Best-response functions . . . 14
  - B.1.2 Nash equilibria when no interior Nash equilibrium exists . . . 16
  - B.1.3 Nash equilibria when matching factors are not reciprocal and an interior equilibrium exists . . . 17
  - B.1.4 Equilibrium abatements with reciprocal matching factors . . . 17
- B.2 Stage-I equilibria: choosing matching factors . . . 19

C Auxiliary proofs for matching-commitment agreements between two countries **21**
- C.1 Characterization of the best-response functions (proof of Lemma B.1) . . . 21
- C.2 Continuity and derivatives of the best-response functions (Lemma B.2) . . . 22
  - C.2.1 Case one . . . 23
  - C.2.2 Case two . . . 23
  - C.2.3 Case three . . . 24
- C.3 Slope of the best-response difference, $\Delta\mathcal{R}$ (Proposition B.6) . . . 25
- C.4 Nash equilibria when no interior equilibrium exists . . . 27
  - C.4.1 The baseline scenario (proof of Proposition B.7) . . . 27
  - C.4.2 If one country will not match, the other will not abate unconditionally (proof of Proposition B.8) . . . 27
  - C.4.3 Nash equilibria when both countries match, but no interior equilibrium exists (proof of Proposition B.9) . . . 27
- C.5 Nash equilibria when no interior equilibrium exists and matching factors are not reciprocal (proof of Proposition B.10) . . . 29
- C.6 Proofs for reciprocal matching factors . . . 30
  - C.6.1 Best-responses for reciprocal matching factors (proof of Lemma B.11) . . . 30
  - C.6.2 Uniqueness of reciprocal matching factors yielding interior Nash equilibria (proof of Lemma B.12) . . . 30
  - C.6.3 Existence of reciprocal matching factors for which interior equilibria exist (proof of Lemma B.13) . . . 32
  - C.6.4 Characterization of the continuum of Nash equilibria (proof of Lemma B.14) . . . 33
- C.7 Map of the matching plane . . . 34
  - C.7.1 The stage-II delimiter curves are well-defined . . . 34
  - C.7.2 The stage-II delimiter curves are differentiable and increasing . . . 34
  - C.7.3 The stage-II delimiter curves are onto $\mathbb{R}_{\geq 0}$ . . . 35
  - C.7.4 Intersections of the stage-II delimiter curves . . . 36
  - C.7.5 Edge equilibria and the stage-II delimiter curves . . . 37
  - C.7.6 The distance between the stage-II delimiter curves . . . 37
- C.8 Multiple possible stage I payoffs under stage-II equilibrium play . . . 37
- C.9 Baseline is not an equilibrium of the matching climate game (proof of Proposition B.16) . . . 39
- C.10 A cooperative equilibrium exists and dominates baseline (proof of Proposition B.17) . . . 40
  - C.10.1 Proof of existence of cooperative equilibrium . . . 40
  - C.10.2 Both countries' payoffs are higher at the cooperative equilibrium emissions profile than at baseline . . . 40

C.10.3 Derivatives of the intercepts of the best-response functions with the unconditional abatement axes . . . 41
C.10.4 Derivative of country 1's payoff with respect to its own matching factor, when only country 2 abates unconditionally . . . 42
C.11 Uniqueness of equilibrium matching factors (proof of Proposition B.18) . . . 44
C.11.1 Region 1 . . . 45
C.11.2 Region 2 . . . 47
C.11.3 Region 3 . . . 50
C.11.4 Region 4 . . . 50
C.11.5 Region 5 . . . 51
C.11.6 On the argument given by Boadway et al. [1] for the uniqueness of the equilibrium . . 51

D Linear-algebraic lemmas **53**

A Model setup

In this appendix, we begin with the stylized model suggested by Boadway et al. [1] to describe national economic considerations for deciding on greenhouse gas (GHG) emissions, which we call the basic climate game (BCG; Definition A.1). Our interest in this study is to compare the outcome of two scenarios: one in which countries independently decide on their emissions levels, *vs.* one in which countries enter a matching-commitment agreement (i.e., play a matching climate game, MCG; see Definition A.9). In order to do so, additional assumptions are necessary to guarantee well-defined outcomes for these scenarios.

Appendix A.1 lays out the assumptions of the BCG between n heterogeneous countries and identifies a sufficient condition for it having a unique Nash equilibrium, which we interpret as the **baseline emissions profile**. That this condition is indeed sufficient for the existence and uniqueness of a Nash equilibrium is established in Appendix A.2. In Appendix A.3, we characterize when a socially optimal emissions profile (which maximizes global welfare) also exists for the BCG. Appendix A.4 characterizes locally Pareto-efficient emissions profiles, and Appendix A.5 compares locally Pareto-efficient emissions profiles with socially optimal ones. Lastly, Appendix A.6 formally defines the matching climate game (MCG; Definition A.9) between n countries and characterizes when the matching-commitment agreement's stage-II best-response functions are well-defined.

A.1 Assumptions, notation and terminology

We first define some convenient notation and terminology. For any emissions profile (i.e., a vector of the emissions of all countries) $\boldsymbol{e} = (e_1, \ldots, e_n) \in \mathbb{R}^n$ and country i ($1 \leq i \leq n$) we denote the vector of the emissions of the $n-1$ countries other than i by

$$\boldsymbol{e}_{-i} = (e_1, \ldots, e_{i-1}, e_i, \ldots, e_n) \; . \tag{A.1}$$

The payoff for country i at an emissions profile $\boldsymbol{e}$ is (with minor abuse of notation)

$$\Pi_i(e_i, \boldsymbol{e}_{-i}) = B_i(e_i) - D_i\left(e\right) \; , \tag{A.2}$$

where $e = \sum_{i=1}^{n} e_i$ denotes the total emissions.

In the notation of Boadway et al. [1], we assume that

$$B_i'(e) > 0\,, \tag{A.3a}$$
$$D_i'(e) > 0\,, \tag{A.3b}$$
$$B_i''(e) < 0\,, \tag{A.3c}$$
$$D_i''(e) > 0\,, \tag{A.3d}$$

for all $1 \leq i \leq n$.

Definition A.1. *The **Basic Climate Game** (BCG) is the game in which countries* $1, \ldots, n$ *simultaneously and non-cooperatively choose their emissions levels to determine their payoffs as follows: if the emissions profile is* $\boldsymbol{e} = (e_1, \ldots, e_n)$*, then country i's payoff* ($1 \le i \le n$) *is*

$$\Pi_i(\boldsymbol{e}) = B_i(e_i) - D_i(e)\,, \tag{A.4}$$

with its benefit and damage functions (B_i *and* D_i*, respectively) satisfying Equation (A.3).*

Note that Equation (A.3) implies that

$$\frac{\partial}{\partial e_i}\Pi_i = B_i'(e_i) - D_i'(e)\ , \tag{A.5a}$$

$$\frac{\partial^2}{\partial {e_i}^2}\Pi_i = B_i''(e_i) - D_i''(e) < 0\,. \tag{A.5b}$$

The BCG does not necessarily have a Nash equilibrium, but the following additional realistic hypothesis ensures that it does[1] (Lemma A.5):

Definition A.2. *We say that global emissions are bounded if there is a box in the space of emissions profiles,*

$$\mathcal{B} = \prod_{i=1}^{n}[e_i^{\mathrm{l}}, e_i^{\mathrm{u}}] \subset \mathbb{R}^n$$

such that

- *for any emissions profile outside* $\mathcal{B}$, $\boldsymbol{e} \in \mathbb{R}^n \setminus \mathcal{B}$, *some country i* ($1 \le i \le n$) *satisfies*

$$\frac{\partial}{\partial e_i}\Pi_i\left(\boldsymbol{e}\right) \neq 0\,; \tag{A.6}$$

- *for any emissions profile on the boundary of* $\mathcal{B}$, $\boldsymbol{e} \in \partial\mathcal{B}$, *if country i's emissions are on the boundary of* $[e_i^{\mathrm{l}}, e_i^{\mathrm{u}}]$*, then it is better off changing its emissions slightly so that* e_i *is in the interior of* $[e_i^{\mathrm{l}}, e_i^{\mathrm{u}}]$*. More precisely, if* $(e_i, \boldsymbol{e}_{-i}) \in \mathcal{B}$ *and* $e_i = e_i^{\mathrm{l}}$ *then*

$$\frac{\partial}{\partial e_i}\Pi_i\left(\boldsymbol{e}\right) > 0\,; \tag{A.7}$$

if $(e_i, \boldsymbol{e}_{-i}) \in \mathcal{B}$ *and* $e_i = e_i^{\mathrm{u}}$ *then*

$$\frac{\partial}{\partial e_i}\Pi_i\left(\boldsymbol{e}\right) < 0\,. \tag{A.8}$$

Lastly, we define BCGs with bounded individual emissions. In these games, no country has an incentive to emit or abate without bound, no matter what other countries do.

Definition A.3. *We say that individual emissions are bounded if for any country i* ($1 \le i \le n$) *and for any emissions profile* $\boldsymbol{e}_{-i}$ *by countries* $1, \ldots, i-1, i+1, \ldots, n$*, there exist emissions levels* e_i^r *and* e_i^l *such that if* $e_i > e_i^r$ *then* $\frac{\partial}{\partial e_i}\Pi_i\left(e_i, \boldsymbol{e}_{-i}\right) < 0$*, and if* $e_i < e_i^l$ *then* $\frac{\partial}{\partial e_i}\Pi_i\left(e_i, \boldsymbol{e}_{-i}\right) > 0$*.*

Because $\frac{\partial^2}{\partial {e_i}^2}\Pi_i\left(e_i, \boldsymbol{e}_{-i}\right) < 0$, individual emissions are bounded if and only if (iff) for any emissions profile $\boldsymbol{e}_{-i}$ by countries $1, \ldots, i-1, i+1, \ldots, n$, there exists e_i for which

$$\frac{\partial}{\partial e_i}\Pi_i\left(e_i, \boldsymbol{e}_{-i}\right) = 0\,. \tag{A.9}$$

Moreover, if e_i satisfies Equation (A.9) then $\Pi_i\left(\cdot, \boldsymbol{e}_{-i}\right)$ has a global maximum at e_i, so e_i is country i's best-response to $\boldsymbol{e}_{-i}$.

[1] Definition A.2 is analogous to the assumption of bounded industry output in Cournot games used in, for example, Kolstad and Mathiesen [2] (Definition 4).

Proposition A.4 (If global emissions are bounded, then individual emissions are bounded)**.** *If countries play a BCG (Definition A.1) and global emissions are bounded (Definition A.2) then individual emissions are bounded, so countries have well-defined best-response functions.*

The following notation will be useful in proving Proposition A.4, as well as in the sequel: for any country i, let

$$\mathcal{B}_{-i} = \prod_{\substack{1 \le j \le n \\ j \ne i}} [e_j^{\mathrm{l}}, e_j^{\mathrm{u}}] \,,$$

be the projection of the box $\mathcal{B}$ onto the space of emissions profiles of all *other* countries.

Proof of Proposition A.4. For any country i ($1 \le i \le n$), Equation (A.3) implies the existence of the following limits:

$$\lim_{e \to \infty} B_i'(e) = \beta_+ \ge 0 \,, \tag{A.10a}$$

$$\lim_{e \to -\infty} B_i'(e) = \beta_- \in (\beta_+, \infty] \,, \tag{A.10b}$$

$$\lim_{e \to -\infty} D_i'(e) = \delta_- \ge 0 \,, \tag{A.10c}$$

$$\lim_{e \to \infty} D_i'(e) = \delta_+ \in (\delta_-, \infty] \,. \tag{A.10d}$$

Let $\boldsymbol{e}_{-i} \in \mathcal{B}_{-i}$. Because global emissions are bounded (Definition A.2), Equation (A.5b) implies

$$0 \le \frac{\partial}{\partial e_i} \Pi_i \left(e_i^{\mathrm{l}}, \boldsymbol{e}_{-i}\right) < \lim_{e \to -\infty} \frac{\partial}{\partial e_i} \Pi_i \left(e_i, \boldsymbol{e}_{-i}\right) = \beta_- - \delta_-$$
$$0 \ge \frac{\partial}{\partial e_i} \Pi_i \left(e_i^{\mathrm{u}}, \boldsymbol{e}_{-i}\right) > \lim_{e \to +\infty} \frac{\partial}{\partial e_i} \Pi_i \left(e_i, \boldsymbol{e}_{-i}\right) = \beta_+ - \delta_+ \,,$$

(with the standard arithmetic and order relation on the extended real line $\overline{\mathbb{R}} = [-\infty, +\infty]$). It follows that for any $\boldsymbol{e}_{-i} \in \mathbb{R}^{n-1}$, we have

$$\lim_{e \to -\infty} \frac{\partial}{\partial e_i} \Pi_i \left(e_i, \boldsymbol{e}_{-i}\right) = \beta_- - \delta_- > 0$$
$$\lim_{e \to +\infty} \frac{\partial}{\partial e_i} \Pi_i \left(e_i, \boldsymbol{e}_{-i}\right) = \beta_+ - \delta_+ < 0 \,,$$

and since $\frac{\partial}{\partial e_i} \Pi_i \left(e_i, \boldsymbol{e}_{-i}\right)$ is continuous, Equation (A.9) has a solution $e_i \in \mathbb{R}$. ☐

A.2 The baseline emissions levels

If $\boldsymbol{e}$ is a Nash equilibrium for the BCG (Definition A.1) with bounded global emissions, then $\boldsymbol{e}$ must be in the interior of the box $\mathcal{B}$ stipulated to exist in Definition A.2. In this section, we establish that the BCG with bounded global emissions has a unique Nash equilibrium $\overline{\boldsymbol{e}} \in \mathcal{B}$, as claimed in the following lemma:

Lemma A.5. *Existence and uniqueness of Nash equilibrium for the BCG with bounded global emissions*

The BCG (Definition A.1) exhibits bounded global emissions (Definition A.2), then it has exactly one Nash equilibrium, which is the unique emissions profile $\overline{\boldsymbol{e}}$ satisfying

$$B_i'(e_i) = D_i'(e) \quad \textit{for all } i = 1, \ldots, n \,. \tag{A.13}$$

Proof. First, observe that Condition A.6 implies that there are no Nash equilibria exist outside of $\mathcal{B}$. Second, using Folmer and von Mouche's [3] theorem 5 (part 2), since the BCG with emissions restricted to $\mathcal{B}$ is a uniformly distributed formal transboundary pollution game (see definitions 1 and 2 in Folmer and von Mouche [3]), the BCG with emissions restricted to $\mathcal{B}$ has a unique Nash equilibrium, which we denote $\overline{\boldsymbol{e}} \in \mathcal{B}$. Lastly, we must show that $\overline{\boldsymbol{e}}$ remains a Nash equilibrium, even when strategies are not restricted to $\overline{\boldsymbol{e}}$, i.e.,

that a unilateral deviation from $\overline{\boldsymbol{e}}$ by country i to an emissions level $e_i \notin [e_i^{\mathrm{l}}, e_i^{\mathrm{u}}]$ does not increase country i's payoff. This follows from the fact that

$$\frac{\partial}{\partial e_i}\Pi_i(\overline{\boldsymbol{e}}) = 0\,, \tag{A.14}$$

(because $e_i = \overline{e}_i$ is the best response to $\overline{\boldsymbol{e}}_{-i}$ when e_i is restricted to$[e_i^{\mathrm{l}}, e_i^{\mathrm{u}}]$), so from Equation (A.5b), $e_i = \overline{e}_i$ is a global maximum of $\Pi_i(e_i; \overline{\boldsymbol{e}}_{-i})$. Lastly, Equation (A.14) is manifestly equivalent to Equation (A.13). □

A.3 The social optimum

Suppose that n countries are playing the BCG, and let Π be the **global welfare**, that is, the total payoffs of all countries,

$$\Pi = \sum_{j=1}^{n} \Pi_j\,.$$

An emissions profile $\widehat{\boldsymbol{e}}$ that locally (resp. globally) maximizes the global welfare Π is a **local (resp. global) social optimum** (SO).

If $\widehat{\boldsymbol{e}}$ is a social optimum, then each country's emissions $\widehat{e}_i$ $(1 \leq i \leq n)$, maximizes global welfare when all other countries' emissions are fixed, that is,

$$\frac{\partial}{\partial e_i}\Pi\left(\widehat{\boldsymbol{e}}\right) = \frac{\partial}{\partial e_i}\sum_{j=1}^{n}\Pi_j\left(\widehat{\boldsymbol{e}}\right) = B_i'\left(\widehat{e}_i\right) - \sum_{j=1}^{n} D_j'\left(\widehat{e}\right) = 0\,.$$

It follows that

$$\frac{\partial}{\partial e_i}\Pi_i\left(\widehat{\boldsymbol{e}}\right) = B_i'\left(\widehat{e}_i\right) - D_i'\left(\widehat{e}\right) > 0\,,$$

so each country has an incentive to increase its emissions at an SO, and hence an SO cannot be a Nash equilibrium of the BCG. Moreover, if the BCG has Nash equilibria, then the total emissions at an SO must be lower than at any Nash equilibrium. To see this, let $\overline{\boldsymbol{e}}$ be a Nash equilibrium, and suppose in order to derive a contradiction that $\widehat{e} \geq \overline{e}$. Then $\widehat{e}_i \geq \overline{e}_i$ for at least one country i $(1 \leq i \leq n)$, which (from Equations (A.3c) and (A.3d)) implies that

$$0 = \frac{\partial}{\partial e_i}\Pi\left(\widehat{\boldsymbol{e}}\right) = B_i'\left(\widehat{e}_i\right) - \sum_{j=1}^{n} D_j'\left(\widehat{e}\right) < B_i'\left(\widehat{e}_i\right) - D_i'\left(\widehat{e}\right) \leq B_i'\left(\overline{e}_i\right) - D_i'\left(\overline{e}\right) = 0\,,$$

a contradiction.

A similar calculation shows that at a Nash equilibrium of the BCG, decreasing *any* country's emissions will increase global welfare:

$$\frac{\partial}{\partial e_i}\Pi\left(\overline{\boldsymbol{e}}\right) = B_i'\left(\overline{e}_i\right) - \sum_{j=1}^{n} D_j'\left(\overline{e}\right) = -\sum_{\substack{1\leq j\leq n\\ j\neq i}} D_j'\left(\overline{e}\right) < 0\,.$$

Proposition A.6. *An emissions profile $\widehat{\boldsymbol{e}}$ is a local SO iff $\boldsymbol{\nabla}\,\Pi = \mathbf{0}$, that is,*

$$B_i'\left(\widehat{e}_i\right) = \sum_{j=1}^{n} D_j'\left(\widehat{e}\right)\,,$$

for each country i $(1 \leq i \leq n)$; moreover, there is at most one local SO, which is necessarily a global SO.

Proof. An SO is a global minimum of $-\Pi$. The Hessian matrix of $-\Pi$ is

$$\begin{aligned}\boldsymbol{\nabla}^2(-\Pi) &= -\begin{pmatrix}\frac{\partial^2}{\partial e_1 \partial e_1}\Pi, & \dots, & \frac{\partial^2}{\partial e_n \partial e_1}\Pi \\ \vdots & & \vdots \\ \frac{\partial^2}{\partial e_1 \partial e_n}\Pi, & \dots, & \frac{\partial^2}{\partial e_n \partial e_n}\Pi\end{pmatrix} \\ &= \begin{pmatrix}-B_1''(e_1) + \sum_{j=1}^n D_j''(e), & \dots, & \sum_{j=1}^n D_j''(e) \\ \vdots & & \vdots \\ \sum_{j=1}^n D_j''(e), & \dots, & -B_n''(e_n) + \sum_{j=1}^n D_j''(e)\end{pmatrix}.\end{aligned}$$

It follows from Corollary D.2 that $-\boldsymbol{\nabla}^2\Pi$ is positive definite. Consequently, $-\Pi$ is everywhere strictly convex [4], which implies that if $\widehat{\boldsymbol{e}}$ solves $\boldsymbol{\nabla}\Pi = \mathbf{0}$ then it is a global maximum of Π. □

The BCG (Definition A.1) is not guaranteed to have an SO. However, a natural necessary and sufficient condition guaranteeing the existence of an SO is that it is not in the global interest to increase or decrease one (or some) country's emissions without bound. Mathematically, this statement is equivalent to the existence of a compact set K outside of which global welfare is lower than its maximum inside K, i.e., such that for any $\boldsymbol{e} \notin K$, $\Pi(\boldsymbol{e}) < \max_K \Pi$.

A.4 Characterization of local Pareto efficiency

In this section, we consider the n-country BCG (Definition A.1) and derive a necessary and sufficient condition[2] for an emissions profile $\boldsymbol{e}$ being locally Pareto efficient, that is, no small deviation from it can increase the payoffs of all countries.

Lemma A.7. *An emissions profile* $\mathbf{e}$ *is locally Pareto efficient iff*

$$\sum_{i=1}^n \frac{D_i'(e)}{B_i'(e_i)} = 1. \tag{A.15}$$

It follows immediately from Lemmas A.5 and A.7 that the baseline emissions profile is *not* Pareto-efficient.

Proof of Lemma A.7. Suppose that $\boldsymbol{e}$ is locally Pareto efficient. We will show that Equation (A.15) holds at $\boldsymbol{e}$.

First, define the Lagrangian

$$\mathcal{L}(\boldsymbol{e}) = \sum_{i=1}^n \lambda_i \Pi_i(\boldsymbol{e}).$$

By theorem 22.15 of Simon and Blume [4], if $\boldsymbol{e}$ is locally Pareto efficient, then there exist $\lambda_i \geq 0$ $(1 \leq i \leq n)$ not all zero, such that

$$\boldsymbol{\nabla}\mathcal{L} = \sum_{i=1}^n \lambda_i \boldsymbol{\nabla}\Pi_i(\boldsymbol{e}) = \mathbf{0}. \tag{A.16}$$

Because $\frac{\partial}{\partial e_j}\Pi_i(\boldsymbol{e}) = \delta_{i,j} B_i'(e_i) - D_i'(e)$, Equation (A.16) becomes

$$\left(\lambda_1 B_1'(e_1) - \sum_{i=1}^n \lambda_i D_i'(e), \dots, \lambda_n B_n'(e_i) - \sum_{i=1}^n \lambda_i D_i'(e)\right) = \mathbf{0},$$

[2] Lemma A.7 is equivalent to Samuelson's [5] condition, as applied to the BCG.

or, setting $\boldsymbol{\lambda} = (\lambda_1, \dots, \lambda_n)^{\mathrm{T}}$,

$$\begin{aligned}
\mathbf{0} &= \begin{pmatrix} B_1'(e_1) - D_1'(e), & \dots, & -D_n'(e) \\ \vdots & & \vdots \\ -D_1'(e), & \dots, & B_n'(e_n) - D_n'(e) \end{pmatrix} \boldsymbol{\lambda} \\
&= \left[\operatorname{diag}\left(B_1'(e_1), \dots, B_n'(e_1) \right) - \begin{pmatrix} D_1'(e), & \dots, & D_n'(e) \\ \vdots & & \vdots \\ D_1'(e), & \dots, & D_n'(e) \end{pmatrix} \right] \boldsymbol{\lambda} . \qquad (A.17)
\end{aligned}$$

Lemma D.3 then implies that when Equation (A.15) holds, the set of solutions of Equation (A.17) is

$$\operatorname{span}\left\{ \left(1/B_1'(e_1), \dots, 1/B_n'(e_1) \right)^{\mathrm{T}} \right\} ; \qquad (A.18)$$

when Equation (A.15) does not hold, Equation (A.17) implies $\lambda_i = 0$ for all $i = 1, \dots, n$. Thus, Equation (A.15) must hold at a local Pareto optimum.

Now suppose that Equation (A.15) holds at $\boldsymbol{e}$. We will show that $\boldsymbol{e}$ is locally Pareto efficient. By theorem 22.17 of Simon and Blume [4], a sufficient condition for the local Pareto efficiency of an emissions profile $\boldsymbol{e}$ is that there exist $\lambda_i \geq 0$ $(1 \leq i \leq n)$ not all zero, such that

(a) Equation (A.16) holds;

(b) for any vector $\boldsymbol{v} \neq 0$ satisfying $\lambda_i \, \boldsymbol{\nabla} \Pi_i \boldsymbol{v} = 0$ for all $i = 1, \dots, n$,

$$\boldsymbol{v}^{\mathrm{T}} \, \boldsymbol{\nabla}^2 \, \mathcal{L} \boldsymbol{v} < 0 . \qquad (A.19)$$

We will check that these conditions hold.

We have already seen that Equation (A.15) implies that Equation (A.16) holds iff

$$\boldsymbol{\lambda} \in \operatorname{span}\left\{ \left(1/B_1'(e_1), \dots, 1/B_n'(e_1) \right)^{\mathrm{T}} \right\} .$$

Since we are interested in non-negative Lagrange multipliers, we restrict attention to

$$\boldsymbol{\lambda} \in \left\{ \lambda \left(1/B_1'(e_1), \dots, 1/B_n'(e_1) \right)^{\mathrm{T}} \,\middle|\, \lambda > 0 \right\} . \qquad (A.20)$$

Now, to verify that there exist nontrivial solutions of Equation (A.16) such that Condition A.19 is satisfied. First, we find the set of vectors $\boldsymbol{v} \neq 0$ satisfying $\lambda_i \, \boldsymbol{\nabla} \Pi_i \boldsymbol{v} = 0$ for all i such that $1 \leq i \leq n$. From Equation (A.20), $\lambda_i > 0$ for $i = 1, \dots, n$, so we must find the kernel of

$$\begin{aligned}
\begin{pmatrix} \boldsymbol{\nabla} \Pi_1 \\ \vdots \\ \boldsymbol{\nabla} \Pi_n \end{pmatrix} &= \begin{pmatrix} \frac{\partial}{\partial e_1} \Pi_1, & \dots, & \frac{\partial}{\partial e_n} \Pi_1 \\ \vdots & & \vdots \\ \frac{\partial}{\partial e_1} \Pi_n, & \dots, & \frac{\partial}{\partial e_n} \Pi_n \end{pmatrix} = \begin{pmatrix} B_1'(e_1) - D_1'(e), & \dots, & -D_n'(e) \\ \vdots & & \vdots \\ -D_1'(e), & \dots, & B_n'(e_n) - D_n'(e) \end{pmatrix} \\
&= \left[\operatorname{diag}\left(B_1'(e_1), \dots, B_n'(e_1) \right) - \begin{pmatrix} D_1'(e), & \dots, & D_n'(e) \\ \vdots & & \vdots \\ D_1'(e), & \dots, & D_n'(e) \end{pmatrix} \right] .
\end{aligned}$$

which, as we have seen, is given by Equation (A.18).

Observe that

$$\begin{aligned}
\boldsymbol{\nabla}^2 \, \mathcal{L} &= \begin{pmatrix} \lambda_1 B_1''(e_1) - \sum_{i=1}^n \lambda_i D_i''(e), & \dots, & -\sum_{i=1}^n \lambda_i D_i''(e) \\ \vdots & & \vdots \\ -\sum_{i=1}^n \lambda_i D_i''(e), & \dots, & \lambda_n B_n''(e_n) - \sum_{i=1}^n \lambda_i D_i''(e) \end{pmatrix} \\
&= \left[\operatorname{diag}\left(\lambda_1 B_1''(e_1), \dots, \lambda_n B_n''(e_1) \right) - \left(\sum_{i=1}^n \lambda_i D_i''(e) \right) \begin{pmatrix} 1, & \dots, & 1 \\ \vdots & & \vdots \\ 1, & \dots, & 1 \end{pmatrix} \right] ,
\end{aligned}$$

and hence,

$$\boldsymbol{v}^{\mathrm{T}}\,\boldsymbol{\nabla}^2\,\mathcal{L}\boldsymbol{v} = \boldsymbol{v}^{\mathrm{T}}\,\mathrm{diag}\left(\lambda_1 B_1''\left(e_1\right),\ldots,\lambda_n B_n''\left(e_1\right)\right)\boldsymbol{v} - \left(\sum_{i=1}^{n}\lambda_i D_i''\left(e\right)\right)\boldsymbol{v}^{\mathrm{T}}\begin{pmatrix}1, & \ldots, & 1\\ \vdots & & \vdots\\ 1, & \ldots, & 1\end{pmatrix}\boldsymbol{v}$$

$$= \sum_{i=1}^{n}\lambda_i B_i''\left(e_i\right)v_i^2 - \left(\sum_{i=1}^{n}\lambda_i D_i''\left(e\right)\right)\left(\sum_{i=1}^{n}v_i\right)^2 .$$

Because $\lambda_i > 0$ $B_i'' < 0$ and $D_i'' > 0$ for all $i = 1,\ldots,n$, it follows that for all $\boldsymbol{v} \neq \boldsymbol{0}$,

$$\boldsymbol{v}^{\mathrm{T}}\,\boldsymbol{\nabla}^2\,\mathcal{L}\boldsymbol{v} \leq \sum_{i=1}^{n}\lambda_i B_i''\left(e_i\right)v_i^2 < 0\,.$$

Condition A.19 therefore holds on the subspace of vectors given in Equation (A.18), which completes our proof[3]. □

A.5 Comparison of Pareto-efficient and socially optimal emissions profiles

Lemma A.8 establishes that a locally Pareto-efficient emissions profile is almost never socially optimal (unless all countries' marginal benefits are equal). Moreover, at a locally Pareto-efficient emissions profile that is *not* socially optimal (SO) a country whose marginal benefits are higher (resp. lower) than all others' emits less (more) than is socially optimal.

Lemma A.8 (Pareto-efficiency *vs.* social optimality)**.** *Consider n countries play the BCG (Definition A.1), and let $\boldsymbol{e}$ be a locally Pareto-efficient emissions profile. Then, $\boldsymbol{e}$ is also SO iff all countries' marginal benefits at $\boldsymbol{e}$ are equal.*

Moreover, suppose that an SO, $\widehat{\boldsymbol{e}}$, exists and is distinct from the Pareto-efficient profile $\boldsymbol{e}$. If i_{M} is a country whose marginal benefit at $\boldsymbol{e}$ is maximal among all countries (i.e., $B_{i_{\mathrm{M}}}'\left(e_{i_{\mathrm{M}}}\right) \geq B_i'\left(e_i\right)$ for all $i = 1,\ldots,n$), then it emits more at the SO than at $\boldsymbol{e}$, $\widehat{e_{i_{\mathrm{M}}}} > e_{i_{\mathrm{M}}}$; similarly, if $B_{i_{\mathrm{m}}}'\left(e_{i_{\mathrm{m}}}\right) \leq B_i'\left(e_i\right)$ for all $i = 1,\ldots,n$, then $\widehat{e_{i_{\mathrm{m}}}} < e_{i_{\mathrm{m}}}$.

Proof. At a locally Pareto-efficient emissions profile $\boldsymbol{e}$, Lemma A.7 implies that

$$B_j'\left(e_j\right) = \sum_{i=1}^{n}\frac{B_j'\left(e_j\right)}{B_i'\left(e_i\right)}D_i'\left(e\right)\,, \tag{A.21}$$

so for any country j $(1 \leq j \leq n)$,

$$\frac{\partial}{\partial e_j}\Pi\left(\boldsymbol{e}\right) = B_j'\left(e_j\right) - \sum_{i=1}^{n}D_i'\left(e\right) = \sum_{i=1}^{n}\left(\frac{B_j'\left(e_j\right)}{B_i'\left(e_i\right)} - 1\right)D_i'\left(e\right)\,. \tag{A.22}$$

If all countries' marginal benefits at the Pareto-efficient emissions profile $\boldsymbol{e}$ are equal, then Equation (A.22) gives $\boldsymbol{\nabla}\,\Pi\left(\boldsymbol{e}\right) = \boldsymbol{0}$, so by Proposition A.6, it is SO.

If not all marginal benefits are equal at the Pareto-efficient emissions profile $\boldsymbol{e}$, let i_{m} be a country with the minimal marginal benefits at $\boldsymbol{e}$, that is,

$$i_{\mathrm{m}} \in \underset{1\leq i\leq n}{\arg\min}\left\{B_i'\left(e_i\right)\right\}\,.$$

Then, for any country i $(1 \leq i \leq n)$, $B_{i_{\mathrm{m}}}'\left(e_{i_{\mathrm{m}}}\right)/B_i'\left(e_i\right) \leq 1$ with a strict inequality for at least one country. Since $D_i'\left(e\right) > 0$ $(1 \leq i \leq n)$, it follows that $\frac{\partial}{\partial e_{i_{\mathrm{m}}}}\Pi\left(\boldsymbol{e}\right) < 0$, and hence $\boldsymbol{e}$ is not socially optimal.

Now, let the country cost and benefit functions be such that an SO emissions profile $\widehat{\boldsymbol{e}}$ exists, and that it is distinct from the Pareto-efficient emissions profile $\boldsymbol{e}$. From our proof thus far, we know that not all

[3]Because we show below that $\boldsymbol{\nabla}^2\,\mathcal{L}$ is negative definite, we actually do not need to restrict the space of $\boldsymbol{v}$'s we consider in Condition A.19.

countries' marginal benefits at the SPE emissions profile are equal. Letting i_{M} be a country with maximal marginal benefits at the Pareto-efficient emissions profile $\boldsymbol{e}$, that is,

$$i_{\mathrm{M}} \in \underset{1\leq i\leq n}{\arg\max}\left\{B_i'\left(e_i\right)\right\} ,$$

(similarly to i_{m} above), we have $i_{\mathrm{m}} \neq i_{\mathrm{M}}$. In the remainder of this proof, we consider the emissions of these two countries, i_{m} and i_{M}.

Observe that Proposition A.6 implies that for any country $j = 1, \ldots, n$, at the SO we have

$$B_j'\left(\widehat{e_j}\right) = \sum_{i=1}^{n} D_i'\left(\widehat{e}\right) .$$

Hence, from Equation (A.21), for any country j, we have

$$B_j'\left(e_j\right) - B_j'\left(\widehat{e_j}\right) = \sum_{i=1}^{n}\left[\frac{B_j'\left(e_j\right)}{B_i'\left(e_i\right)} D_i'\left(e\right) - D_i'\left(\widehat{e}\right)\right] . \tag{A.23}$$

Suppose, in order to derive a contradiction, that country i_{m}'s emissions at the SO are no less than at the Pareto-efficient emissions profile, i.e., $\widehat{e_{i_{\mathrm{m}}}} \geq e_{i_{\mathrm{m}}}$. Then for any $i = 1, \ldots, n$, we have

$$B_i'\left(e_i\right) \geq B_{i_{\mathrm{m}}}'\left(e_{i_{\mathrm{m}}}\right) \geq B_{i_{\mathrm{m}}}'\left(\widehat{e_{i_{\mathrm{m}}}}\right) = B_i'\left(\widehat{e_i}\right) ,$$

($B_i'\left(e_i\right)$ decreases in e_i; see Equation (A.3c)). Thus, $\widehat{e_i} \geq e_i$ for all $i = 1, \ldots, n$, and the inequality must be strict for at least one country (because we assume that $\boldsymbol{e}$ is not SO), so $\widehat{e} > e$. Then, Equation (A.23) gives

$$B_{i_{\mathrm{m}}}'\left(e_{i_{\mathrm{m}}}\right) - B_{i_{\mathrm{m}}}'\left(\widehat{e_{i_{\mathrm{m}}}}\right) = \sum_{i=1}^{n}\left[\frac{B_{i_{\mathrm{m}}}'\left(e_{i_{\mathrm{m}}}\right)}{B_i'\left(e_i\right)} D_i'\left(e\right) - D_i'\left(\widehat{e}\right)\right] \leq \sum_{i=1}^{n}\left[\frac{B_{i_{\mathrm{m}}}'\left(e_{i_{\mathrm{m}}}\right)}{B_i'\left(e_i\right)} - 1\right] D_i'\left(e\right) < 0 ,$$

where the rightmost inequality follows because $B_{i_{\mathrm{m}}}'\left(e_{i_{\mathrm{m}}}\right) \leq B_i'\left(e_i\right)$ for all $i = 1, \ldots, n$ with a strict inequality for $i = i_{\mathrm{M}}$. Hence $B_{i_{\mathrm{m}}}'\left(e_{i_{\mathrm{m}}}\right) < B_{i_{\mathrm{m}}}'\left(\widehat{e_{i_{\mathrm{m}}}}\right)$, so that $e_{i_{\mathrm{m}}} > \widehat{e_{i_{\mathrm{m}}}}$, a contradiction. Thus, $e_{i_{\mathrm{m}}} > \widehat{e_{i_{\mathrm{m}}}}$ must hold.

Similarly, suppose that $e_{i_{\mathrm{M}}} \geq \widehat{e_{i_{\mathrm{M}}}}$. Then for any $i = 1, \ldots, n$, we have

$$B_i'\left(e_i\right) \leq B_{i_{\mathrm{M}}}'\left(e_{i_{\mathrm{M}}}\right) \leq B_{i_{\mathrm{M}}}'\left(\widehat{e_{i_{\mathrm{M}}}}\right) = B_i'\left(\widehat{e_i}\right) ,$$

which implies that $e_i \geq \widehat{e_i}$, so that $e > \widehat{e}$ and hence $D_i'\left(\widehat{e}\right) < D_i'\left(e\right)$. Then Equation (A.23) gives

$$B_j'\left(e_j\right) - B_j'\left(\widehat{e_j}\right) > \sum_{i=1}^{n}\left[\frac{B_j'\left(e_j\right)}{B_i'\left(e_i\right)} - 1\right] D_i'\left(e\right) ,$$

and consequently, for i_{M},

$$B_{i_{\mathrm{M}}}'\left(e_{i_{\mathrm{M}}}\right) > B_{i_{\mathrm{M}}}'\left(\widehat{e_{i_{\mathrm{M}}}}\right) ,$$

so $\widehat{e_{i_{\mathrm{M}}}} > e_{i_{\mathrm{M}}}$, contradicting our assumption. Thus, $\widehat{e_{i_{\mathrm{M}}}} > e_{i_{\mathrm{M}}}$ holds. □

A.6 The Matching Climate Game

We define the Matching Climate Game for n countries:

Definition A.9. *Suppose that n countries have payoffs Π_i ($\imath = 1, \ldots, n$) determined by their own emissions (e_i) and the emissions of all other countries $\boldsymbol{e}_{-i}$) as follows:*

$$\Pi_i(e_i, \boldsymbol{e}_{-i}) = B_i(e_i) - D_i\left(e\right) , \quad \textit{for all} \quad i = 1, \ldots, n . \tag{A.24}$$

Suppose in addition that benefits and damages are decelerating and accelerating functions of emissions (Equation (A.3)) and that global emissions are bounded (Definition A.2). Let the baseline emissions profile $\overline{\boldsymbol{e}}$ be the unique Nash equilibrium emissions profile (guaranteed by Lemma A.5).

We say that the countries are playing the ***Matching Climate Game*** *(MCG) if*

- *the countries play the following two stage game:*

  Stage I: *Countries simultaneously and non-cooperatively choose their (non-negative) matching factors, $m_{i,j} \geq 0$, to which they are subsequently committed.*

  Stage II: *Countries simultaneously and non-cooperatively choose their unconditional abatement levels, a_i, with full knowledge of the matching factors chosen in stage I.*

- *country i's payoff is given by*

$$\Pi_i = B_i\left(\overline{e}_i - A_i\right) - D_i\left(\overline{e} - A\right) , \tag{A.25}$$

where each country's abatement A_i is given by

$$A_i = a_i + \sum_{\substack{1 \leq j \leq n \\ j \neq i}} m_{i,j} a_j , \tag{A.26}$$

$\overline{e} = \sum_{i=1}^n \overline{e}_i$ *are the total baseline emissions and* $A = \sum_{i=1}^n A_i$ *is total abatement.*

Some additional notation is convenient in the n-country setting. Letting $m_{i,i} = 1$ for all $i = 1, \ldots, n$, we have

$$A_i = \sum_{j=1}^n m_{i,j} a_j .$$

We also denote the vector of unconditional abatements by $\boldsymbol{a}$, the vector of the *nonfocal* (i.e., other) countries' unconditional abatements by $\boldsymbol{a}_{-i} = (a_1, \ldots, a_{i-1}, a_{i+1}, \ldots, a_n)$ and the vector of the factors at which the focal country i matches the unconditional abatements of the nonfocal countries by $\boldsymbol{m}^{\mathrm{p}}_{-i} = (m_{i,1}, \ldots, m_{i,i-1}, m_{i,i+1}, \ldots, m_{i,n})$ (the superscript "p" indicates that i performs this matching). For $i = 1, \ldots, n$, let the total matching received by country i be

$$m_i^{\mathrm{r}} = \sum_{\substack{1 \leq j \leq n \\ j \neq i}} m_{j,i} ,$$

(the superscript "r" indicates that i is the recipient of this matching).

It is in general possible that a given choice of matching factors in the MCG's stage I can incentivize some country to abate infinitely, and hence the MCG's stage-II best-response functions may not be well-defined. Proposition A.10 gives necessary and sufficient conditions guaranteeing the existence of well-defined best-response functions for stage II of the MCG for any choice of matching factors $m_{i,j} \geq 0$ ($i, j = 1, \ldots, n$, $i \neq j$). Because this condition guarantees that no matching factors can incentivize infinite abatement (see Equation (A.31) below), we call this condition **bounded abatement with matching (BAM)**.

Proposition A.10 (The MCG's stage-II best-response functions are well defined iff the BAM condition holds)**.** *Suppose that n countries are parties to an MCG. The countries' stage-II best-response functions are well defined for all $\boldsymbol{a}_{-i} \in \mathbb{R}^{n-1}_{\geq 0}$, given any $m_{i,j} \geq 0$ and $m_{j,i} \geq 0$ ($j = 1, \ldots, i, i+1, \ldots, n$), iff for each $i = 1, \ldots, n$ either*

$$\lim_{e \to -\infty} B_i'(e) = \infty , \tag{A.27a}$$

or

$$\lim_{e \to -\infty} D_i'(e) = 0 . \tag{A.27b}$$

Proof. Fix a focal country i and note that

$$\frac{\partial}{\partial a_i}\Pi_i = -B_i'\left(\overline{e}_i - a_i - \boldsymbol{m}^{\mathrm{p}}_{-i}\boldsymbol{a}_{-i}\right) + (1 + m_i^{\mathrm{r}}) D_i'\left(\overline{e} - \sum_{k=1}^n (1 + m_k^{\mathrm{r}}) a_k\right) , \tag{A.28}$$

and

$$\frac{\partial^2}{\partial {a_i}^2}\Pi_i = B_i''\left(\overline{e}_i - a_i - \boldsymbol{m}^{\mathrm{p}}_{-i}\boldsymbol{a}_{-i}\right) - (1 + m_i^{\mathrm{r}})^2 D_i''\left(\overline{e} - \sum_{k=1}^n (1 + m_k^{\mathrm{r}}) a_k\right) < 0 . \tag{A.29}$$

Let $m_{i,j} \geq 0$, $m_{j,i} \geq 0$ $(j = 1, \ldots, i-1, i+1, \ldots, n)$ and $\boldsymbol{a}_{-i} \in \mathbb{R}^{n-1}_{\geq 0}$. If

$$\frac{\partial}{\partial a_i}\Pi_i\bigg|_{a_i=0} = -B'_i\left(\bar{e}_i - \boldsymbol{m}^{\mathrm{p}}_{-i}\boldsymbol{a}_{-i}\right) + (1+m^{\mathrm{r}}_i)D'_i\left(\bar{e} - \sum_{\substack{1\leq j\leq n\\ j\neq i}}(1+m^{\mathrm{r}}_j)a_j\right) \leq 0\,,$$

then Equation (A.29) implies that $\frac{\partial}{\partial a_i}\Pi_i$ decreases with a_i, so country i's best-response to $\boldsymbol{a}_{-i}$, given $m_{i,j}$ and $m_{j,i}$ $(j = 1, \ldots, i, i+1, \ldots, n)$, is 0. Suppose then that

$$\frac{\partial}{\partial a_i}\Pi_i\bigg|_{a_i=0} > 0\,,$$

(note that this holds for any fixed $\boldsymbol{m}^{\mathrm{p}}_{-i} \geq 0$ and $\boldsymbol{a}_{-i} \geq 0$ if the matching received by i, m^{r}_i, is sufficiently large). Because $\frac{\partial}{\partial a_i}\Pi_i$ decreases with a_i, (Equation (A.29)), the limit $\lim_{a_i\to\infty}\frac{\partial}{\partial a_i}\Pi_i$ exists and

$$\lim_{a_i\to\infty}\frac{\partial}{\partial a_i}\Pi_i \in [-\infty, +\infty)\,.$$

It follows that if

$$\lim_{a_i\to\infty}\frac{\partial}{\partial a_i}\Pi_i > 0\,,$$

then Π_i increases for all a_i, and country i's best-response to so country i's best-response to $\boldsymbol{a}_{-i}$, given $m_{i,j}$ and $m_{j,i}$ $(j = 1, \ldots, i, i+1, \ldots, n)$, is unbounded, and thus undefined. Conversely, if

$$\lim_{a_i\to\infty}\frac{\partial}{\partial a_i}\Pi_i < 0\,,$$

then there exists a unique a_i satisfying $\frac{\partial}{\partial a_i}\Pi_i = 0$; Π_i is maximal at this a_i, and hence country i's best-response to $\boldsymbol{a}_{-i}$, given $m_{i,j}$ and $m_{j,i}$ $(j = 1, \ldots, i, i+1, \ldots, n)$, is well-defined and equal to a_i.

To summarize what we have learned so far: Let $\boldsymbol{a}_{-i} \in \mathbb{R}^{n-1}_{\geq 0}$, $m_{i,j} \geq 0$ and $m_{j,i} \geq 0$ $(j = 1, \ldots, i, i+1, \ldots, n)$.

- If

$$\lim_{a_i\to\infty}\frac{\partial}{\partial a_i}\Pi_i > 0\,, \tag{A.30}$$

  that is, if $m_{i,j} \geq 0$ and $m_{j,i} \geq 0$ $(j = 1, \ldots, i, i+1, \ldots, n)$ incentivize country i to abate infinitely, then country i's best-response to $\boldsymbol{a}_{-i}$, given $m_{i,j}$ and $m_{j,i}$ $(j = 1, \ldots, i, i+1, \ldots, n)$, is undefined.

- If

$$\lim_{a_i\to\infty}\frac{\partial}{\partial a_i}\Pi_i < 0\,, \tag{A.31}$$

  then country i's best-response to $\boldsymbol{a}_{-i}$, given $m_{i,j}$ and $m_{j,i}$ $(j = 1, \ldots, i, i+1, \ldots, n)$, is well-defined.

Now, observe that $D''_i > 0$, so $D'_i(\bar{e} - a)$ decreases with a and is positive, and hence has a non-negative limit,

$$\lim_{a\to\infty} D'_i(\bar{e}_i - a) = \lim_{e\to-\infty} D'_i(e) = \delta_- \in [0, \infty)\,.$$

Similarly, since $B''_i < 0$ and $B'_i > 0$

$$\lim_{a\to\infty} B'_i(\bar{e}_i - a) = \lim_{e\to-\infty} B'_i(e) = \beta_- \in (0, \infty]\,,$$

($\beta_- = 0$ is impossible because $B_i''(\bar{e}_i - a) > 0$ and increases with a). If $\beta_- = \infty$ then Equation (A.28) gives

$$\lim_{a_i \to \infty} \frac{\partial}{\partial a_i} \Pi_i = -\infty \,,$$

and hence country i's best-response is well defined for all $\boldsymbol{a}_{-i}$, $m_{i,j}$ and $m_{j,i}$ ($j = 1, \ldots, i, i+1, \ldots, n$).

If $\beta_- \in (0, \infty)$ then Equation (A.28) gives

$$\lim_{a_i \to \infty} \frac{\partial}{\partial a_i} \Pi_i = (1 + m_i^{\mathrm{r}})\delta_- - \beta_- \,,$$

and hence for any $\beta_- \in (0, \infty)$ and $\delta_- > 0$, there exists m_i^{r} large enough that

$$\lim_{a_i \to \infty} \frac{\partial}{\partial a_i} \Pi_i > 0 \,.$$

Thus, if $\beta_- \in (0, \infty)$, country i's best-response is well defined for all $\boldsymbol{a}_{-i}$, $m_{i,j}$ and $m_{j,i}$ ($j = 1, \ldots, i, i+1, \ldots, n$), iff $\delta_- = 0$.

We have thus proved that country i's stage-II best-response to $\boldsymbol{a}_{-i}$ is well defined for all $m_{i,j}$ and $m_{j,i}$ ($j = 1, \ldots, i, i+1, \ldots, n$), iff either

$$\lim_{e \to -\infty} B_i'(e) = \infty \,, \tag{A.32a}$$

or

$$\lim_{e \to -\infty} D_i'(e) = 0 \,; \tag{A.32b}$$

if neither of these conditions are met then there are values of $\boldsymbol{a}_{-i}$, $m_{i,j}$ and $m_{j,i}$ ($j = 1, \ldots, i, i+1, \ldots, n$), for which country i's best-response is unbounded. Equation (A.32) means that at the limit of negative unbounded emissions (or infinite abatement), either the marginal benefit of emissions is infinite, or the marginal damage of emissions vanishes. □

B Analysis of the matching climate game for two countries

B.1 Stage-II equilibria: choosing unconditional abatement levels

In stage II of the MCG, countries choose their unconditional emissions abatements (relative to their baseline emissions, $\bar{e}_i$), given matching factors that were chosen in stage I, and country i's payoff is then

$$\Pi_i = B_i(\bar{e}_i - A_i) - D_i(\bar{e} - A) \,.$$

To find the best-response functions for the two countries, observe that if country j's unconditional abatement is a_j, country i's best-response is defined by

$$\mathcal{R}_i(m_1, m_2; a_j) = \underset{a_i \geq 0}{\arg\max} \left\{ \Pi_i \right\} = \underset{a_i \geq 0}{\arg\max} \left\{ B_i(\bar{e}_i - A_i) - D_i(\bar{e} - A) \right\} . \tag{B.33}$$

In Appendix A.6 we show that under the assumptions of the BCG (Definition A.1) the stage-II best-response functions are well-defined and bounded iff for each country $i = 1, 2$

$$\lim_{e \to -\infty} B_i'(e) = \infty \,, \tag{B.34a}$$

or

$$\lim_{e \to -\infty} D_i'(e) = 0 \,. \tag{B.34b}$$

Because an unbounded best-response is unrealistic, we henceforth assume that one of these conditions holds (for each country $i = 1, 2$).

Given matching factors m_1 and m_2, solutions of

$$a_1 = \mathcal{R}_1(m_1, m_2; a_2) \,, \tag{B.35a}$$

$$a_2 = \mathcal{R}_2(m_1, m_2; a_1) \,, \tag{B.35b}$$

constitute the set of Nash equilibria for the second stage of the game.

B.1.1 Best-response functions

Under our assumptions, the best-response functions are well-defined (Appendix A.6). Since the expected payoffs are differentiable, country i's best-response to $m_1 \geq 0$, $m_2 \geq 0$ and a_j, $a_i = \mathcal{R}_i(m_1, m_2; a_j)$, is either on the boundary ($a_i = 0$) or (using Equation (A.25)) satisfies[4]

$$\frac{\partial}{\partial a_i}\Pi_i = -B_i'(\bar{e}_i - A_i) + (1+m_j)D_i'(\bar{e} - A) = 0\,. \tag{B.36}$$

The following lemmas (proved in Appendices C.1 and C.2) characterize the best-response functions.

Lemma B.1 (Characterization of the best-response functions)**.** *If $m_j = 0$, then country i's best-response is to abate nothing unconditionally, regardless of country j's unconditional abatement, $\mathcal{R}_i(m_1, m_2; a_j) \equiv 0$.*

If $m_j > 0$ then $\mathcal{R}_i(m_1, m_2; a_j)$ intercepts the a_j axis at a unique point $a_{i,j}^{\text{int}}(m_1, m_2) > 0$ such that $\mathcal{R}_i(m_1, m_2; a_j) > 0$ for all a_j satisfying $0 \leq a_j < a_{i,j}^{\text{int}}$, and $\mathcal{R}_i(m_1, m_2; a_j) = 0$ for all $a_j \geq a_{i,j}^{\text{int}}$. Moreover, $a_{i,j}^{\text{int}}$ is the unique solution of

$$\left.\frac{\partial}{\partial a_i}\Pi_i\right|_{a_i=0} = -B_i'(\bar{e}_i - m_i a_j) + (1+m_j)D_i'(\bar{e} - (1+m_i)a_j) = 0\,. \tag{B.37}$$

Lemma B.2 (Continuity and derivatives of the best-response functions)**.** *$\mathcal{R}_i(m_1, m_2; a_j)$ is continuous in its arguments, m_1, m_2 and a_j.*

Moreover, if $\vec{p} = (m_1, m_2, a_j) \in \mathbb{R}^3_{\geq 0}$ satisfies $\mathcal{R}_i(\vec{p}) \geq 0$, then[5]

$$\frac{\partial}{\partial a_j}\mathcal{R}_i(m_1, m_2; a_j) = -\left.\frac{m_i B_i''(\bar{e}_i - A_i) - (1+m_i)(1+m_j)D_i''(\bar{e} - A)}{B_i''(\bar{e}_i - A_i) - (1+m_j)^2 D_i''(\bar{e} - A)}\right|_{a_i = \mathcal{R}_i(m_1, m_2; a_j)} < 0\,, \tag{B.38a}$$

$$\frac{\partial}{\partial m_i}\mathcal{R}_i(m_1, m_2; a_j) = -a_j\left.\frac{B_i''(\bar{e}_i - A_i) - (1+m_j)D_i''(\bar{e} - A)}{B_i''(\bar{e}_i - A_i) - (1+m_j)^2 D_i''(\bar{e} - A)}\right|_{a_i = \mathcal{R}_i(m_1, m_2; a_j)} < 0\,, \tag{B.38b}$$

$$\frac{\partial}{\partial m_j}\mathcal{R}_i(m_1, m_2; a_j) = -\left.\frac{D_i'(\bar{e} - A) - (1+m_j)D_i''(\bar{e} - A)a_i}{B_i''(\bar{e}_i - A_i) - (1+m_j)^2 D_i''(\bar{e} - A)}\right|_{a_i = \mathcal{R}_i(m_1, m_2; a_j)}\,. \tag{B.38c}$$

In particular, wherever country i's best-response function does not vanish, it is decreasing in country j's unconditional abatement.

Remark B.3 (Country i's stage-II best-response when country j does not abate unconditionally)**.** *If $m_j > 0$, $\mathcal{R}_i(m_1, m_2; a_j)$ decreases for $a_j \in [0, a_{i,j}^{\text{int}}]$, and $\mathcal{R}_i(m_1, m_2; a_{i,j}^{\text{int}}) = 0$, so $\mathcal{R}_i(m_1, m_2; a_j)$ intercepts the a_i-axis at a positive height $a_{i,i}^{\text{int}}(m_1, m_2) = \mathcal{R}_i(m_1, m_2; 0) > 0$. Using Equation (B.36), $a_{i,i}^{\text{int}}$ is the unique solution of*

$$\left.\frac{\partial}{\partial a_i}\Pi_i\right|_{a_j=0} = -B_i'(\bar{e}_i - a_i) + (1+m_j)D_i'(\bar{e} - (1+m_j)a_i) = 0\,. \tag{B.39}$$

Figure B.1 schematically illustrates the intercepts of country i's stage-II best-response function $\mathcal{R}_i$ with the unconditional abatement axes, $a_{i,i}^{\text{int}}$ and $a_{i,j}^{\text{int}}$.

The following observations will also be of use:

Remark B.4 (Country i's stage-II best response function when country j does not match)**.** *If $m_j = 0$, $\mathcal{R}_i(m_1, m_2; a_j) \equiv 0$ (Lemma B.1), so the intercept of $\mathcal{R}_i$ with the a_i axis is $a_{i,i}^{\text{int}} = 0$. Note that even when $m_j = 0$, $a_{i,i}^{\text{int}} = 0$ is the unique solution of Equation (B.39).*

In this case, we also let $a_{i,j}^{\text{int}} = 0$, so that (similar to the case when $m_j > 0$), $\mathcal{R}_i(m_1, m_2; a_j) = 0$ for all $a_j \geq a_{i,j}^{\text{int}}$.

Remark B.5. *If $m_1 > 0$ or $m_2 > 0$, then $(a_{i,i}^{\text{int}}, 0)$ is a Nash equilibrium iff $a_{i,i}^{\text{int}} \geq a_{j,i}^{\text{int}}$ ($i, j \in \{1, 2\}$, $j \neq i$).*

[4] Since $\frac{\partial}{\partial a_i}A_i = 1$ and, $A = A_1 + A_2 = (1+m_i)a_j + (1+m_j)a_i$, so $\frac{\partial}{\partial a_i}A = 1 + m_j$.

[5] In Equation (B.38), if $\vec{p} = (m_1, m_2, a_j) \in \partial\mathbb{R}^3_{\geq 0}$, some of the derivatives are interpreted as right-hand derivatives.

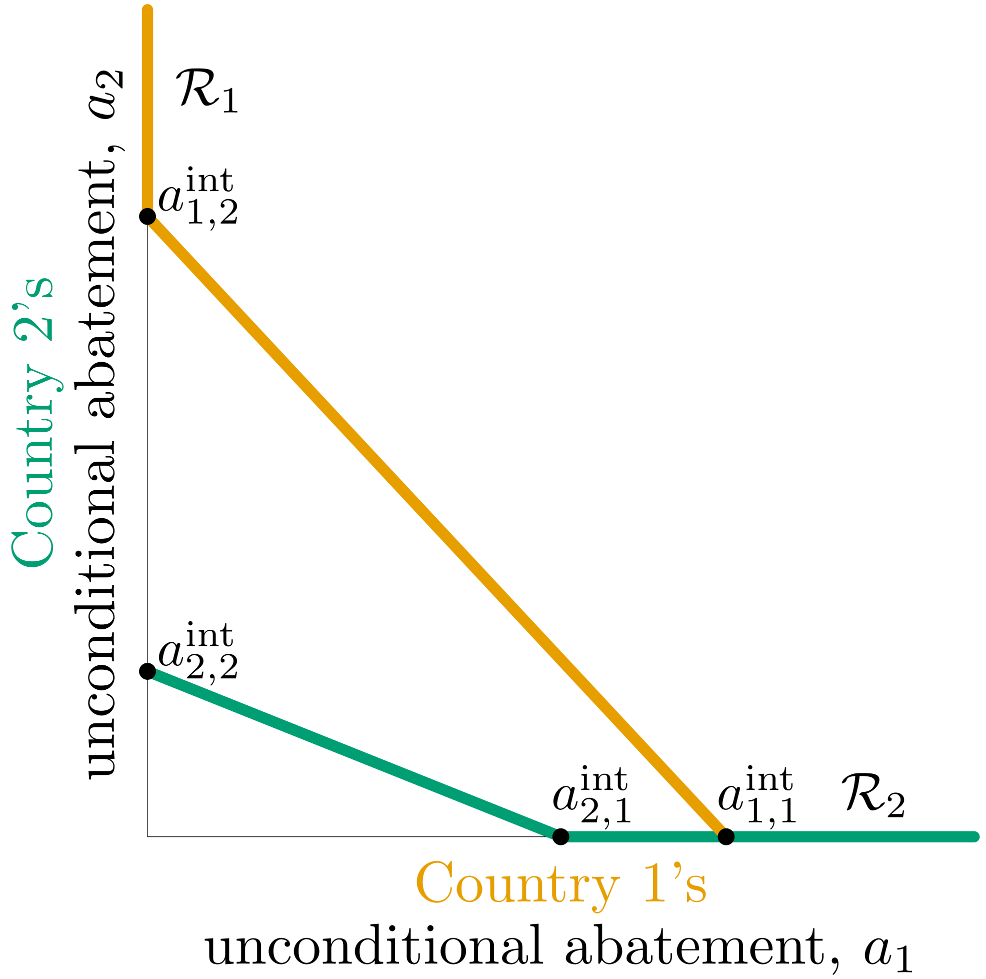

Figure B.1: Schematic illustration of the intercepts of the matching climate game's (MCG) stage-II best-response functions with the unconditional abatement axes. For a given pair of matching factors, (m_1, m_2), country i's stage-II best-response function $\mathcal{R}_i$ intercepts the a_j axis at $a_{i,j}^{\mathrm{int}}$, which is implicitly defined by Equation (B.37), and intercepts the a_i axis at $a_{i,i}^{\mathrm{int}} = \mathcal{R}_i(m_1, m_2; 0)$, which is implicitly defined by Equation (B.39).

Fixing matching factors $m_i > 0$ $(i = 1, 2)$, it is convenient to analyze the *vertical* distance between the two best-response functions in the a_1–a_2 plane,

$$\Delta\mathcal{R}(m_1, m_2; a_1) = \mathcal{R}_2(m_1, m_2; a_1) - \mathcal{R}_1^{-1}(m_1, m_2; a_1)\,,$$

(since Nash equilibria are intersections of the best-response functions; see Equation (B.35)). The following proposition, proved in Appendix C.3, gives the slope of $\Delta\mathcal{R}(m_1, m_2; a_1)$ as a function of a_1.

Proposition B.6 (Slope of the best-response difference for fixed matching factors)**.** *If $m_i > 0$ for $i = 1, 2$, then $\Delta\mathcal{R}(m_1, m_2; a_1)$ is continuously differentiable with respect to a_1 for all $a_1 \in (0, \min\{a_{1,1}^{\text{int}}, a_{2,1}^{\text{int}}\})$, and*

$$\begin{aligned}\frac{\partial}{\partial a_1}\Delta\mathcal{R}(m_1, m_2; a_1) &= \frac{\partial}{\partial a_1}\mathcal{R}_2(m_1, m_2; a_1) - \frac{\partial}{\partial a_1}\mathcal{R}_1^{-1}(m_1, m_2; a_1)\,,\\ &= -\left.\frac{m_2 B_2''\left(\bar{e}_2 - A_2\right) - \left(1 + m_2\right)\left(1 + m_1\right) D_2''\left(\bar{e} - A\right)}{B_2''\left(\bar{e}_2 - A_2\right) - \left(1 + m_1\right)^2 D_2''\left(\bar{e} - A\right)}\right|_{a_2 = \mathcal{R}_2(m_1, m_2; a_1)}\\ &\quad + \left.\frac{B_1''\left(\bar{e}_1 - A_1\right) - \left(1 + m_2\right)^2 D_1''\left(\bar{e} - A\right)}{m_1 B_1''\left(\bar{e}_1 - A_1\right) - \left(1 + m_1\right)\left(1 + m_2\right) D_1''\left(\bar{e} - A\right)}\right|_{a_2 = \mathcal{R}_1^{-1}(m_1, m_2; a_1)}.\end{aligned}$$

In particular, if an interior Nash equilibrium (a_1^*, a_2^*) exists, then

$$\begin{aligned}\frac{\partial}{\partial a_1}\Delta\mathcal{R}(m_1, m_2; a_1^*) &= \frac{\left(1 - m_1 m_2\right)}{\left[\left(1 + m_1\right)^2 D_2''\left(\bar{e} - A\right) - B_2''\left(\bar{e}_2 - A_2\right)\right]\left[\left(1 + m_1\right)\left(1 + m_2\right) D_1''\left(\bar{e} - A\right) - m_1 B_1''\left(\bar{e}_1 - A_1\right)\right]}\\ &\quad \times \Big[B_1''\left(\bar{e}_1 - A_1\right) B_2''\left(\bar{e}_2 - A_2\right) - (1 + m_1) B_1''\left(\bar{e}_1 - A_1\right) D_2''\left(\bar{e} - A\right)\\ &\qquad - (1 + m_2) B_2''\left(\bar{e}_2 - A_2\right) D_1''\left(\bar{e} - A\right)\Big]\end{aligned} \tag{B.40}$$

where A_1, A_2 and A are evaluated at the Nash equilibrium (a_1^*, a_2^*). All the terms in square brackets are positive, so $\frac{\partial}{\partial a_1}\Delta\mathcal{R}(m_1, m_2; a_1^*)$ is positive if $m_1 m_2 < 1$, negative if $m_1 m_2 > 1$ and vanishes if $m_1 m_2 = 1$.

For equilibria $(a_i^*, a_j^*) = (a_{i,i}^{\text{int}}, 0)$, such that $a_{i,i}^{\text{int}} = a_{j,i}^{\text{int}}$, analogues of Equation (B.40) hold, where the derivative is replaced with an appropriate one-sided derivative.

B.1.2 Nash equilibria when no interior Nash equilibrium exists

When no interior Nash equilibria exist, the only possible equilibria are ones in which at least one country does not abate unconditionally. We refer to equilibria on the a_1- or a_2- axes excluding $(0, 0)$ as **edge equilibria**, because they are on the edges of the set of possible strategy combinations in the second stage of the game, $\{(a_1, a_2) \mid a_i \geq 0 \text{ for } i = 1, 2\}$. The strategy profile $(a_i^*, a_j^*) = (0, 0)$ results in the baseline emissions profile $\bar{\boldsymbol{e}}$, and hence will also be referred to as the baseline emissions profile.

Finding the Nash equilibria is particularly simple when one or both countries' best-response functions are identically 0: there will be no interior Nash equilibria; either both countries do not abate, or one country induces the other to abate unconditionally (but does not itself abate unconditionally). The next two propositions, proved in Appendices C.4.1 and C.4.2, formally characterize these two possibilities.

Proposition B.7 (The baseline scenario)**.** *$(a_i^*, a_j^*) = (0, 0)$ is a Nash equilibria iff $m_1 = m_2 = 0$.*

When $m_i = 0$ and $m_j > 0$, then country j's best-response is identically 0, but country i's is not, so the unique Nash equilibrium is one in which only country i abates unconditionally:

Proposition B.8 (If one country will not match, the other will not abate unconditionally)**.** *If $m_i = 0$ and $m_j > 0$, then the unique Nash equilibrium is $(a_i^*, a_j^*) = (a_{i,i}^{\text{int}}, 0)$.*

Proposition B.9 (proved in Appendix C.4.3) lists the possibilities in the more complicated situation in which both countries match (so neither country's best-response function is identically 0), but no interior equilibria exist. In this case, either one country can induce the other to abate unconditionally alone, or the two countries play a game of "chicken" as regards their unconditional abatements (the latter is only possible when $m_1 m_2 > 1$).

Proposition B.9 (Nash equilibria when both countries match, but no interior equilibrium exists)**.** *Let $m_i > 0$ for $i = 1, 2$ such that there are no interior Nash equilibria. Then,*

if $m_1 m_2 < 1$**:** *If $a_{1,1}^{\text{int}} \geq a_{2,1}^{\text{int}}$ (or equivalently, $a_{1,2}^{\text{int}} > a_{2,2}^{\text{int}}$) then the unique Nash equilibrium is $(a_1^*, a_2^*) = (a_{1,1}^{\text{int}}, 0)$; otherwise, $a_{2,1}^{\text{int}} > a_{1,1}^{\text{int}}$, $a_{2,2}^{\text{int}} \geq a_{1,2}^{\text{int}}$, and the unique Nash equilibrium is $(a_1^*, a_2^*) = (0, a_{2,2}^{\text{int}})$;*

if $m_1 m_2 = 1$**:** *If $a_{1,1}^{\text{int}} > a_{2,1}^{\text{int}}$ (or equivalently, $a_{1,2}^{\text{int}} > a_{2,2}^{\text{int}}$) then the unique Nash equilibrium is $(a_1^*, a_2^*) = (a_{1,1}^{\text{int}}, 0)$; otherwise, $a_{2,1}^{\text{int}} > a_{1,1}^{\text{int}}$, $a_{2,2}^{\text{int}} > a_{1,2}^{\text{int}}$, and the unique Nash equilibrium is $(a_1^*, a_2^*) = (0, a_{2,2}^{\text{int}})$;*

if $m_1 m_2 > 1$**:** *Either one or two (edge) Nash equilibria exist.*

- *If $a_{1,2}^{\text{int}} > a_{2,2}^{\text{int}}$ then $a_{1,1}^{\text{int}} > a_{2,1}^{\text{int}}$ and the unique Nash equilibrium is $(a_1^*, a_2^*) = (a_{1,1}^{\text{int}}, 0)$;*
- *If $a_{2,1}^{\text{int}} > a_{1,1}^{\text{int}}$ then $a_{2,2}^{\text{int}} > a_{1,2}^{\text{int}}$ and the unique Nash equilibrium is $(a_1^*, a_2^*) = (0, a_{2,2}^{\text{int}})$;*
- *Both $(a_{1,1}^{\text{int}}, 0)$ and $(0, a_{2,2}^{\text{int}})$ are Nash equilibria iff either*

$$\begin{cases} a_{1,1}^{\text{int}} & > a_{2,1}^{\text{int}}\,, \\ a_{2,2}^{\text{int}} & = a_{1,2}^{\text{int}}\,, \end{cases} \qquad \textit{or} \qquad \begin{cases} a_{1,1}^{\text{int}} & = a_{2,1}^{\text{int}}\,, \\ a_{2,2}^{\text{int}} & > a_{1,2}^{\text{int}}\,. \end{cases}$$

B.1.3 Nash equilibria when matching factors are not reciprocal and an interior equilibrium exists

The following proposition, proved in Appendix C.5, shows that if $m_1 m_2 \neq 1$ and there exists an interior Nash equilibrium, then it is either the only Nash equilibrium, or there are two additional edge equilibria.

Proposition B.10 (Nash equilibria when $m_1 m_2 \neq 1$ and an interior equilibrium exists)**.** *If $m_1 m_2 \neq 1$ and an interior Nash equilibrium (a_1^*, a_2^*) exists, then:*

If $m_1 m_2 > 1$ *then the set of Nash equilibria is*

$$\big\{(a_{1,1}^{\text{int}}, 0), (0, a_{2,2}^{\text{int}}), (a_1^*, a_2^*)\big\}.$$

Moreover, $a_{1,1}^{\text{int}} > a_{2,1}^{\text{int}}$ and $a_{2,2}^{\text{int}} > a_{1,2}^{\text{int}}$.

If $m_1 m_2 < 1$ *then (a_1^*, a_2^*) is the unique Nash equilibrium. Moreover, $a_{2,1}^{\text{int}} > a_{1,1}^{\text{int}}$ and $a_{1,2}^{\text{int}} > a_{2,2}^{\text{int}}$.*

B.1.4 Equilibrium abatements with reciprocal matching factors

Let $\mathcal{M}_{\text{r}}$ be the set of reciprocal matching factors,

$$\mathcal{M}_{\text{r}} = \big\{(m_1, m_2) \,\big|\, m_i > 0 \text{ for } i = 1, 2\,, \text{ and } m_1 m_2 = 1\big\}\,.$$

We will show that

- there is a unique pair of reciprocal matching factors $(m_1^{\text{c}}, m_2^{\text{c}}) \in \mathcal{M}_{\text{r}}$ for which a continuum of interior Nash equilibria in unconditional abatements exists. For $(m_1, m_2) = (m_1^{\text{c}}, m_2^{\text{c}})$, each country's total abatement is constant over the set of equilibrium unconditional abatements, so while unconditional abatements at equilibrium are indeterminate the equilibrium payoffs are uniquely defined.
- for all $(m_1, 1/m_1) \in \mathcal{M}_{\text{r}}$ ($m_1 > 0$) such that $m_1 \neq m_1^{\text{c}}$, the unique Nash equilibrium is an edge equilibrium. In particular,
  - if $0 < m_1 < m_1^{\text{c}}$, the unique Nash equilibrium is $(a_1^*, a_2^*) = \big(a_{1,1}^{\text{int}}, 0\big)$;
  - if $m_1 > m_1^{\text{c}}$, the unique Nash equilibrium is $(a_1^*, a_2^*) = \big(0, a_{2,2}^{\text{int}}\big)$.

Our claims are justified in the following sequence of lemmas (proved in Appendix C.6):

Lemma B.11 (Best-responses for reciprocal matching factors)**.** *If* $(m_1, m_2) \in \mathcal{M}_\mathrm{r}$*, then* $a_{i,i}^{\mathrm{int}} > 0$*,* $a_{i,j}^{\mathrm{int}} > 0$ *and*

$$\mathcal{R}_i(m_1, m_2; a_j) = \begin{cases} -m_i a_j + a_{i,i}^{\mathrm{int}} & \text{if } 0 \le a_j \le a_{i,j}^{\mathrm{int}}\,, \\ 0 & \text{if } a_j > a_{i,j}^{\mathrm{int}}\,. \end{cases} \tag{B.41}$$

In particular, $a_{i,i}^{\mathrm{int}} = m_i a_{i,j}^{\mathrm{int}}$*. Moreover,* $\mathcal{R}_i(m_1, m_2; a_j)$ *is injective for* $0 \le a_j \le a_{i,j}^{\mathrm{int}}$*, and thus possesses an inverse in the range* $0 \le a_i \le a_{i,i}^{\mathrm{int}}$*,*

$$\mathcal{R}_i^{-1}(m_1, m_2; a_i) = -m_j a_i + a_{i,j}^{\mathrm{int}}\,.$$

Thus, $\mathcal{R}_j^{-1}(m_1, m_2; a_j)$ is parallel to country i's best-response function $\mathcal{R}_i(m_1, m_2; a_j)$ in the interior of the quadrant of positive unconditional abatements,. There are then two possible cases:

- $\mathcal{R}_i(m_1, m_2; a_j) > \mathcal{R}_j^{-1}(m_1, m_2; a_j)$ for all $a_j \in \left[0, \min\left\{a_{i,j}^{\mathrm{int}}, a_{j,j}^{\mathrm{int}}\right\}\right]$ for $(i, j) = (1, 2)$ or $(2, 1)$: In this case, country i's best-response function is always *above* country j's in the interior of that quadrant. Then, the unique Nash equilibrium is the edge equilibrium $(a_i^*, a_j^*) = \left(a_{i,i}^{\mathrm{int}}, 0\right)$.
- The two best-response functions overlap in the interior of that quadrant. In this case, for any $a_1 \in [0, a_{1,1}^{\mathrm{int}}]$, $(a_1^*, a_2^*) = \left(a_1, \mathcal{R}_2(m_1, m_2; a_1)\right)$ is a Nash equilibrium, that is, the equilibrium unconditional abatements are indeterminate.

The next two lemmas show that there exists a unique pair of matching factors $(m_1, m_2) \in \mathcal{M}_\mathrm{r}$ for which there are interior Nash equilibria in unconditional abatements:

Lemma B.12 (Uniqueness of reciprocal matching factors yielding interior Nash equilibria)**.** *If* $(m_1, m_2) \in \mathcal{M}_\mathrm{r}$*,*

- $\Delta\mathcal{R}(m_1, m_2; a_1)$ *is constant for all* $a_1 \in [0, \min(a_{1,1}^{\mathrm{int}}, a_{2,1}^{\mathrm{int}})]$ *and thus can be written as* $\Delta\mathcal{R}\,(m_1)$*;*
- *if* $\Delta\mathcal{R}\,(m_1) = 0$ *then* $\frac{\mathrm{d}}{\mathrm{d}m_1}\Delta\mathcal{R}\,(m_1) > 0$*.*

Thus, there is at most a unique pair of reciprocal matching factors $(m_1^\mathrm{c}, m_2^\mathrm{c}) \in \mathcal{M}_\mathrm{r}$ *for which the two country's best-response functions overlap in the interior of the quadrant of positive unconditional abatements.*

Lemma B.13 (Existence of reciprocal matching factors yielding interior Nash equilibria)**.** *There exists a pair of matching factors,* $(m_1^\mathrm{c}, m_2^\mathrm{c}) \in \mathcal{M}_\mathrm{r}$ *such the best-response functions overlap in the interior of the quadrant of positive unconditional abatements.*

Lemma B.14 combines the results of the previous lemmas with characterizations of the reciprocal matching factors for which there is a continuum of Nash equilibria, and of the set of Nash equilibria.

Lemma B.14 (Characterization of Nash equilibria for reciprocal matching factors)**.** *There are unique* $m > 0$ *and* $a > 0$ *satisfying*

$$B_1'\left(\bar{e}_1 - a\right) = (1+m) D_1'\big(\bar{e} - (1+m)a\big)\,, \tag{B.42a}$$

$$m B_2'\left(\bar{e}_2 - ma\right) = (1+m) D_2'\big(\bar{e} - (1+m)a\big)\,. \tag{B.42b}$$

Then, letting $(m_1^\mathrm{c}, m_2^\mathrm{c}) = (1/m, m)$*, we have* $(m_1^\mathrm{c}, m_2^\mathrm{c}) \in \mathcal{M}_\mathrm{r}$*. Moreover, for any pair of reciprocal matching factors* $(m_1, m_2) \in \mathcal{M}_\mathrm{r}$*,*

- *if* $0 < m_1 < m_1^\mathrm{c}$ *the unique Nash equilibrium is* $(a_1^*, a_2^*) = \left(a_{1,1}^{\mathrm{int}}, 0\right)$*;*
- *if* $m_1 > m_1^\mathrm{c}$*, the unique Nash equilibrium is* $(a_1^*, a_2^*) = \left(0, a_{2,2}^{\mathrm{int}}\right)$*;*
- *if* $m_1 = m_1^\mathrm{c}$*, the set of Nash equilibria are*

$$\left\{ \left(a_1, \mathcal{R}_2(m_1, m_2; a_1)\right) \,\middle|\, a_1 \in \left[0, a_{1,1}^{\mathrm{int}}\right] \right\}\;;$$

on this set of Nash equilibria, the countries' abatements remain constant and equal to $A_1 = a$ *and* $A_2 = ma$*.*

Figure 3 consolidates the results of this appendix (Appendix B.1) about the existence of stage-II equilibria for each pair of matching factors, dividing the set of non-negative matching factors $\mathbb{R}^2_{\geq 0}$ into regions in which the stage-II best-response functions behave differently. In Appendix C.7, we justify Figure 3 by showing that for a given $m_j \geq 0$, the equation

$$a^{\text{int}}_{i,i}(m_1, m_2) = a^{\text{int}}_{j,i}(m_1, m_2)\,, \tag{B.43}$$

can be solved to give the matching factor $m_i = \phi_i(m_j)$ at which the intercepts of $\mathcal{R}_1$ and $\mathcal{R}_2$ with the a_i axis are equal. Moreover, we show that the **stage-II delimiter curves** ϕ_i ($i = 1, 2$) are differentiable, increasing, unbounded, and have the following properties:

- these curves intersect only at $m_1 = m_2 = 0$ and at $(m^{\text{c}}_1, m^{\text{c}}_2)$;
- the curve $\phi_1^{-1}(m_1)$ is above (resp. below) $\phi_2(m_1)$ in the region $m_1 m_2 < 1 (> 1)$;
- for any $(m_i, m_j) \neq (0, 0)$, $(a^*_i, a^*_j) = (a^{\text{int}}_{i,i}, 0)$ is a stage-II Nash equilibrium iff $m_i \leq \phi_i(m_j)$.

Remark B.15 (On Boadway et al.'s [1] argument identifying the SPE matching factors)**.** *Boadway et al.'s proof of their Proposition 1 (on the existence and uniqueness of an SPE) relies on the assertion that there is a unique pair of reciprocal matching factors (i.e., satisfying $m_1 m_2 = 1$) such that the two stage-II best-response functions overlap in the quadrant of positive unconditional abatements (similar to the conclusions of Lemmas B.12 and B.13).*

Boadway et al.'s [1] argument for the uniqueness *of such matching factors (in footnote 5 on page 356) is as follows: if (m_1, m_2) are reciprocal, and such that the two best-response functions overlap for positive unconditional abatements, then a small increase in country 1's matching factor, from m_1 to m'_1, (without changing country 2's matching factor) causes country 2's stage-II best-response function to be everywhere above country 1's; a subsequent decrease in country 2's matching factor (keeping country 1's matching factor at its new value, m'_1) has the same effect. Consequently, the stage-II best response functions do not overlap for positive unconditional abatements when $m'_2 = 1/m'_1$ and $m'_1 > m_1$ (an analogous claim holds for $m'_1 < m_1$).*

In justifying this argument, Boadway et al. infer whether one of the best-response functions is above the other in the quadrant of positive unconditional abatements from their intersections with the a_1-axis. This is manifestly valid when matching factors are reciprocal, because the stage-II best-response functions are parallel for positive unconditional abatements. But when (m'_1, m_2) are played, $m'_1 m_2 \neq 1$, so the stage-II best-response functions need not be parallel, and two additional assertions are needed to complete this argument:

1. *When the countries' matching factors are (m'_1, m_2) the two countries' stage-II best-response functions do not intersect in the quadrant of positive unconditional abatements. This is justified in the second paragraph of page 366 of Boadway et al. [1] (though not referred to in footnote 5).*
2. *The stage-II best-response functions are continuous (which Boadway et al. implicitly assumed). Our Proposition A.10 and Lemma B.2 establish that this is indeed true iff the BAM condition holds.*

Along with these assertions, Boadway et al.'s [1] argument establishes the uniqueness of a pair of reciprocal matching factors for which the two stage-II best-response functions overlap; however the existence *of such matching factors does not follow from this argument. In order to do so, an argument similar to the proof of Lemma B.13 can be used.*

B.2 Stage-I equilibria: choosing matching factors

For any pair of matching factors, each choice of Nash equilibria in the second stage defines the two players' payoffs for these matching factors; however, for matching factors m_i ($i = 1, 2$) that yield multiple Nash equilibria in stage II, each choice of equilibrium may yield a different payoff. From Appendix B.1, we know that for a given pair of matching factors, m_i ($i = 1, 2$), there are either one, two , three or a continuum of Nash equilibria in unconditional abatements. Thus, infinitely many possible combinations of Nash equilibria in stage II give rise to infinitely many possible payoff functions for stage I, even when we assume equilibrium play in stage II.

Conveniently, when equilibrium play in stage II is assumed, the pair of matching factors for which a continuum of equilibria exists in stage II, $(m^{\text{c}}_1, m^{\text{c}}_2)$, has uniquely defined payoffs: we observed (in Lemma B.14)

that when a continuum of Nash equilibria exists, the countries' abatements are constant over the set of equilibrium unconditional abatement levels, and thus the total abatement is also constant over this set. Since the payoff functions depend only on the two countries' abatements (rather than their unconditional abatements), if a continuum of equilibria exists, the countries' expected payoffs are constant over the set of equilibrium unconditional abatements. Thus, when a continuum of equilibria exists, the second-stage equilibrium payoffs of the two countries are well-defined and given by Equation (A.25) (evaluated at any of the equilibria).

Consequently, any pair of matching factors $m_1, m_2 \geq 0$ yields either one, two or three possible equilibrium payoffs (assuming equilibrium play in stage II):

- three payoffs are possible in stage I for all m_1, m_2 yielding an interior Nash equilibrium and two edge equilibria in stage II;
- two payoffs are possible in stage I for all m_1, m_2 yielding two edge equilibria (and no interior equilibrium) in stage II;
- otherwise, the stage I payoff is uniquely defined.

Let $\mathcal{A}$ be the set of **stage-II equilibrium choices**, that is, pairs of functions, $a_i\left(m_1, m_2\right) : \mathbb{R}^2_{\geq 0} \to \mathbb{R}$ such that for any pair of matching factors, $(m_1, m_2) \in \mathbb{R}^2_{\geq 0}$, if (m_1, m_2) are played in stage I then

$$\big(a_1\left(m_1, m_2\right), a_2\left(m_1, m_2\right)\big)\,,$$

is a Nash equilibrium in stage II. Any such stage-II equilibrium choice $\big(a_1\left(m_1, m_2\right), a_2\left(m_1, m_2\right)\big) \in \mathcal{A}$ defines a continuous-strategy 2-player game in stage I by setting (with slight abuse of notation)

$$\Pi_i(m_1, m_2) = \Pi_i\big(m_1, m_2, a_1\left(m_1, m_2\right), a_2\left(m_1, m_2\right)\big)\,. \tag{B.44}$$

Letting $\mathcal{E}_{\text{mult}}$ be the set of matching factors for which there are multiple equilibria, we note that $(m_1^{\text{c}}, m_2^{\text{c}}) \in \mathcal{E}_{\text{mult}}$, and for $(m_1^{\text{c}}, m_2^{\text{c}})$ there are infinitely many possible Nash equilibria in stage II. Thus, there are infinitely pairs of functions $a_i\left(m_1, m_2\right)$ $(i = 1, 2)$ in $\mathcal{A}$. Moreover, in Appendix C.8 we show that there are infinitely many pairs of matching factors yielding multiple stage-II equilibria, and for which different stage-II equilibria generate different payoffs. Consequently, there are infinitely many possible games in stage I, depending on the choice of equilibria in stage II. Note that the multiplicity of possible stage I payoffs for a given pair of matching factors (assuming equilibrium play in stage II) implies that the two countries' payoff functions in the resulting stage I game need not be continuous.

If the stage I game has a Nash equilibrium (μ_1, μ_2), then the two-stage (i.e., full) game has an SPE, in which the two countries' strategy profiles are $\big(\mu_i, a_i\left(m_1, m_2\right)\big)$ $i = 1, 2$. Conversely, if a strategy profile $\big(\mu_i, a_i\left(m_1, m_2\right)\big)$ $(i = 1, 2)$ is an SPE, then if (m_1, m_2) is played in stage I then $\big(a_1\left(m_1, m_2\right), a_2\left(m_1, m_2\right)\big)$ is one of the possible Nash equilibria in stage II (for any (m_1, m_2)), and hence (μ_1, μ_2) is a Nash equilibrium for the stage I game with payoffs given by Equation (B.44).

A matching-commitment agreement achieves two things: first, it causes the baseline emissions profile to cease being an equilibrium (Proposition B.16 proved in Appendix C.9); second, it generates a cooperative SPE (i.e., one in which both countries abate) for any stage-II equilibrium choice (see Proposition B.17 proved in Appendix C.10). Importantly, both countries do better at the cooperative SPE emissions profile identified in Proposition B.17 than they do at their baseline emissions levels.

Proposition B.16. *The baseline scenario is* not *an SPE outcome of the MCG.*

Proposition B.17 (A cooperative SPE exists and dominates baseline)**.** *Let $m > 0$ and $a >$ be the unique positive solutions of*

$$B_1'\left(\bar{e}_1 - a\right) = (1 + m) D_1'\big(\bar{e} - (1 + m)a\big)\,, \tag{B.45a}$$

$$m B_2'\left(\bar{e}_2 - ma\right) = (1 + m) D_2'\big(\bar{e} - (1 + m)a\big)\,, \tag{B.45b}$$

and let $\left(m_i^{\text{c}}, m_j^{\text{c}}\right) = (1/m, m)$.

For any stage-II equilibrium choice

$$\big(a_1\left(m_1, m_2\right), a_2\left(m_1, m_2\right)\big) \in \mathcal{A}\,, \tag{B.46}$$

the strategy profile

$$\left(m_i^{\mathrm{c}}, a_i\left(m_1, m_2\right)\right) \quad \text{for } i = 1, 2\,,$$

is an SPE. At any such SPE, the countries' abatements are $A_1 = a$ *and* $A_2 = ma$*, and the total abatement is* $A = (1+m)a$*. Moreover, both countries' payoffs are higher at the SPE emissions profile than at the baseline emissions profile* $\bar{\boldsymbol{e}}$*.*

Proposition B.17 implies that the MCG generates multiple SPEs (because there are multiple possible choices for the stage-II equilibria; see Equation (B.46)). However, we show in Proposition B.18 (proved in Appendix C.11) that only $(m_1^{\mathrm{c}}, m_2^{\mathrm{c}})$ are played on an SPE path. When $(m_1, m_2) = (m_1^{\mathrm{c}}, m_2^{\mathrm{c}})$ is played in stage I the abatements A_i and payoffs Π_i $(i = 1, 2)$ do not depend on the choice of stage-II Nash equilibria, so the SPE outcome is for all intents and purposes uniquely defined[6].

Proposition B.18 (Uniqueness of subgame-perfect emissions profile)**.** *If*

$$\left(m_i^{\mathrm{c}}, a_i\left(m_1, m_2\right)\right) \quad \text{for } i = 1, 2\,,$$

is an SPE, then $m_i = m_i^{\mathrm{c}}$ $(i = 1, 2)$ *are as in Proposition B.17. Thus, while multiple SPEs exist, the subgame-perfect emissions profile is uniquely determined and given by*

$$(e_1, e_2) = (\bar{e}_1 - a, \bar{e}_2 - ma)\ ,$$

with m *and* a *as in Proposition B.17. Consequently, the SPE payoffs are also uniquely determined.*

Lastly, Equation (B.42) implies that Equation (A.15) holds at the SPE emissions profile, so from Lemma A.7, it is locally Pareto efficient. This, along with Propositions B.17 and B.18 justifies the following:

Theorem B.19. *Consider two countries playing the MCG (Definition A.9) for which the BAM condition holds (Equation (A.27)). Then, the MCG has infinitely many SPEs, but there is a unique pair of matching factors* $(m_1^{\mathrm{c}}, m_2^{\mathrm{c}})$ *that is played in all SPEs. The equilibrium abatement* (A_i) *levels (and therefore payoffs* Π_i*) for the MCG are uniquely defined in the sense that they do not depend on which SPE is chosen, and result in a locally Pareto efficient emissions profile*[7]*. Moreover, at an SPE of the MCG both countries' emissions are lower, but their payoffs are higher than at the baseline emissions profile (i.e., the Nash equilibrium of the BCG, in which these countries independently and simultaneously select their emissions levels). The equilibrium matching factors, abatements and emissions profile are given (implicitly) in Propositions B.17 and B.18.*

C Auxiliary proofs for matching-commitment agreements between two countries

C.1 Characterization of the best-response functions (proof of Lemma B.1)

Because

$$\frac{\partial^2}{\partial {a_i}^2}\Pi_i = \frac{\partial^2}{\partial {a_i}^2}\Big[B_i\left(\bar{e}_i - A_i\right) - D_i\left(\bar{e} - A\right)\Big] = B_i''\left(\bar{e}_i - A_i\right) - (1+m_j)^2 D_i''\left(\bar{e} - A\right) < 0\,, \tag{C.47}$$

it follows that if

$$\left.\frac{\partial}{\partial a_i}\Pi_i\right|_{a_i=0} \le 0\,,$$

then country i's best-response is to abate nothing,

$$\mathcal{R}_i(m_1, m_2; a_j) = \operatorname*{arg\,max}_{a_i \ge 0}\left\{\Pi_i\right\} = 0\,;$$

[6]Boadway et al. [1] claim that the SPE they identify is unique. However, their justification for this claim is problematic; see Appendix C.11.6.

[7]By locally Pareto efficient, we mean that a small deviation from the SPE emissions profile must decrease at least one country's payoff.

otherwise, there is a unique positive level of unconditional abatement $a_i > 0$ that solves Equation (B.36), and

$$\mathcal{R}_i(m_1, m_2; a_j) = \underset{a_i \geq 0}{\arg\max} \left\{\Pi_i\right\} = a_i \,.$$

Now, recalling that $\overline{\boldsymbol{e}}$ is a Nash equilibrium,

$$\frac{\partial}{\partial e_i}\Pi_i\left(\bar{e}_i, \overline{\boldsymbol{e}}_{-i}\right) = B_i'\left(\bar{e}_i\right) - D_i'\left(\bar{e}\right) = 0\,,$$

which implies that

$$\left.\frac{\partial}{\partial a_i}\Pi_i\right|_{a_i=a_j=0} = -B_i'\left(\bar{e}_i\right) + (1+m_j)D_i'\left(\bar{e}\right) = m_j D_i'\left(\bar{e}\right)\,,$$

so $\left.\frac{\partial}{\partial a_i}\Pi_i\right|_{a_i=a_j=0} = 0$ if $m_j = 0$ and $\left.\frac{\partial}{\partial a_i}\Pi_i\right|_{a_i=a_j=0} > 0$ otherwise. Noting that

$$\left.\frac{\partial}{\partial a_i}\Pi_i\right|_{a_i=0} = -B_i'(\bar{e}_i - m_i a_j) + (1+m_j)D_i'\left(\bar{e} - (1+m_i)a_j\right)\,,$$

we have

$$\frac{\partial}{\partial a_j}\left[\left.\frac{\partial}{\partial a_i}\Pi_i\right|_{a_i=0}\right] = m_i B_i''(\bar{e}_i - m_i a_j) - (1+m_i)(1+m_j)D_i''\left(\bar{e} - A\right) < 0\,, \tag{C.48}$$

and hence $\left.\frac{\partial}{\partial a_i}\Pi_i\right|_{a_i=0}$ decreases with a_j. It follows that if $m_j = 0$ then $a_{i,j}^{\text{int}} = 0$ for all a_j. If $m_j > 0$ then using Equation (B.34),

$$\lim_{a_j\to\infty}\left.\frac{\partial}{\partial a_i}\Pi_i\right|_{a_i=0} < 0\,,$$

so there exists $a_{i,j}^{\text{int}} > 0$ such that $\left.\frac{\partial}{\partial a_i}\Pi_i\right|_{a_i=0} > 0$ for all $a_j \in (0, a_{i,j}^{\text{int}})$, and $\left.\frac{\partial}{\partial a_i}\Pi_i\right|_{a_i=0} \leq 0$ for all $a_j \geq a_{i,j}^{\text{int}}$. Consequently, $\mathcal{R}_i(m_1, m_2; a_j) > 0$ for all $a_j < a_{i,j}^{\text{int}}$, and $\mathcal{R}_i(m_1, m_2; a_j) = 0$ for all $a_j \geq a_{i,j}^{\text{int}}$.

C.2 Continuity and derivatives of the best-response functions (Lemma B.2)

We show that $\mathcal{R}_i(m_1, m_2; a_j)$ is continuous in its domain of definition, i.e., for any triplet of parameters $\vec{p} = (m_1, m_2, a_j) \in \mathbb{R}^3_{\geq 0}$. We will divide the domain of $\mathcal{R}_i(m_1, m_2; a_j)$ into the three following cases:

1. $\vec{p} = (m_1, m_2, a_j) \in \mathbb{R}^3_{\geq 0}$ such that $\mathcal{R}_i(\vec{p}) = 0$;
2. $\vec{p} = (m_1, m_2, a_j) \in \mathbb{R}^3_{>0}$ such that $\mathcal{R}_i(\vec{p}) > 0$;
3. $\vec{p} = (m_1, m_2, a_j) \in \partial\,\mathbb{R}^3_{\geq 0}$ such that $\mathcal{R}_i(\vec{p}) > 0$.

These cases are analyzed in Appendices C.2.1 to C.2.3, respectively.

We will use Lemma C.1, which relates country i's stage-II best-response function to the derivative of its expected payoff Π_i with respect to its own unconditional abatement a_i; Lemma C.1 follows immediately from the fact that $\frac{\partial^2}{\partial {a_i}^2}\Pi_i < 0$ for all $a_i \geq 0$ (Equation (C.47)).

Lemma C.1.

1. *If* $\mathcal{R}_i(\vec{p}) = 0$ *then* $\left.\frac{\partial}{\partial a_i}\Pi_i\right|_{\vec{p}} < 0$ *for all* $a_i > 0$.
2. *If* $\mathcal{R}_i(\vec{p}) > 0$ *then* $a_i = \mathcal{R}_i(\vec{p})$ *is the unique solution of*

$$\frac{\partial}{\partial a_i}\Pi_i = -B_i'\left(\bar{e}_i - A_i\right) + (1+m_j)D_i'\left(\bar{e} - A\right) = 0\,.$$

Moreover, $\left.\frac{\partial}{\partial a_i}\Pi_i\right|_{\vec{p}} > 0$ *for all* $a_i \in \big[0, \mathcal{R}_i(\vec{p})\big)$ *and* $\left.\frac{\partial}{\partial a_i}\Pi_i\right|_{\vec{p}} < 0$ *for all* $a_i \in \big(\mathcal{R}_i(\vec{p}), \infty\big)$.

C.2.1 Case one: continuity of $\mathcal{R}_i(\vec{p})$ for $\vec{p} \in \mathbb{R}^3_{\geq 0}$ such that $\mathcal{R}_i(\vec{p}) = 0$

Consider a triplet of arguments $\vec{p} = (m_1, m_2, a_j) \in \mathbb{R}^3_{\geq 0}$ for which $\mathcal{R}_i(\vec{p}) = 0$, and a sequence $\{\vec{p}_r\}_{r=0}^{\infty} \subset \mathbb{R}^3_{\geq 0}$ such that $\vec{p}_r \xrightarrow{r\to\infty} \vec{p}$. We wish to show that

$$\lim_{r\to\infty} \mathcal{R}_i(\vec{p}_r) = 0 = \mathcal{R}_i(\vec{p}) = \mathcal{R}_i(\lim_{r\to\infty} \vec{p}_r)\,. \tag{C.49}$$

Our proof proceeds as follows: we first show that $\mathcal{R}_i(\vec{p}_r)$ is bounded, and then that 0 is the unique partial limit of $\mathcal{R}_i(\vec{p}_r)$; these two facts imply Equation (C.49).

Suppose, in order to derive a contradiction, that $\mathcal{R}_i(\vec{p}_r)$ is not bounded. Then, because $\mathcal{R}_i(\cdot) \geq 0$, without loss of generality, we can assume that $\mathcal{R}_i(\vec{p}_r)$ is increasing, $\mathcal{R}_i(\vec{p}_r) \xrightarrow{r\to\infty} \infty$ and that $\mathcal{R}_i(\vec{p}_r) > 0$ for all $r \geq 0$. From Lemma C.1, for any integer $r \geq 0$, $\frac{\partial}{\partial a_i}\Pi_i\left(\vec{p}_r; a_i\right)$ is positive for all $a_i \in \big[0, \mathcal{R}_i(\vec{p}_r)\big)$ and negative for all $a_i \in \big(\mathcal{R}_i(\vec{p}_r), \infty\big)$. In particular, fixing any $a_i > 0$, there is a large enough integer $R \geq 0$ such that $\frac{\partial}{\partial a_i}\Pi_i\left(\vec{p}_r; a_i\right) > 0$ for all $r \geq R$. However, $\frac{\partial}{\partial a_i}\Pi_i$ is manifestly continuous in the arguments m_1, m_2 and a_j, and $\mathcal{R}_i(\vec{p}) = 0$ implies that $\frac{\partial}{\partial a_i}\Pi_i\left(\vec{p}; a_i\right) < 0$ for all $a_i > 0$ (Lemma C.1), so for r large enough, $\frac{\partial}{\partial a_i}\Pi_i\left(\vec{p}_r; a_i\right) < 0$, which is a contradiction. Consequently, $\mathcal{R}_i(\vec{p}_r)$ must be bounded.

Let $\{\vec{p}_{r_k}\}_{k\geq 0}$ be a subsequence of $\{\vec{p}_r\}_{r\geq 0}$ such that $\{\mathcal{R}_i(\vec{p}_{r_k})\}_{k\geq 0}$ is convergent, and suppose in order to derive a contradiction, that

$$\lim_{k\to\infty} \mathcal{R}_i(\vec{p}_{r_k}) = a_{i,\infty} > 0\,.$$

Because $\mathcal{R}_i(\vec{p}_{r_k}) = 0$ for at most finitely many k's (otherwise, 0 would be a partial limit of $\mathcal{R}_i(\vec{p}_{r_k})$, contradicting the assumption that $\lim_{k\to\infty} \mathcal{R}_i(\vec{p}_{r_k}) > 0$), no generality is lost by assuming that $\mathcal{R}_i(\vec{p}_{r_k}) > 0$ for all integer $k \geq 0$.

Since $\mathcal{R}_i(\vec{p}_{r_k}) > 0$,

$$\left.\frac{\partial}{\partial a_i}\Pi_i\left(\vec{p}_{r_k}; a_i\right)\right|_{a_i=\mathcal{R}_i(\vec{p}_{r_k})} = 0 \text{ for all } k \geq 0\,,$$

(Lemma C.1) and because $\frac{\partial}{\partial a_i}\Pi_i$ is manifestly continuous in m_1, m_2, a_j and a_i, the limit $k \to \infty$ gives

$$\frac{\partial}{\partial a_i}\Pi_i\left(\vec{p}; a_{i,\infty}\right) = 0\,.$$

From Lemma C.1, this implies that $\mathcal{R}_i(\vec{p}) = a_{i,\infty} > 0$, which contradicts our assumption that $\mathcal{R}_i(\vec{p}) = 0$. Thus, 0 is the unique partial limit of $\mathcal{R}_i(\vec{p}_r)$, which concludes our proof of Equation (C.49).

C.2.2 Case two: continuous differentiability of $\mathcal{R}_i(\vec{p})$ for $\vec{p} \in \mathbb{R}^3_{>0}$ such that $\mathcal{R}_i(\vec{p}) > 0$

Let $\vec{p} = (m_1, m_2, a_j) \in \mathbb{R}^3_{>0}$ satisfy $\mathcal{R}_i(\vec{p}) > 0$, Because $\frac{\partial^2}{\partial {a_i}^2}\Pi_i < 0$ (Equation (C.47)) and $\frac{\partial}{\partial a_i}\Pi_i$ is continuously differentiable in all its variables, it follows from the implicit function theorem [6] that there is a neighbourhood of $\vec{p}$ in which $\mathcal{R}_i(\vec{p})$ is defined implicitly by Equation (B.36) and continuously differentiable with respect to its arguments.

Now, from Lemma B.1, $\mathcal{R}_i(m_1, m_2; a_j) > 0$ for all a_j such that $0 \leq a_j < a^{\text{int}}_{i,j}$. Observe that

$$\frac{d}{da_j}\left(A_i|_{a_i=\mathcal{R}_i(m_1,m_2;a_j)}\right) = \frac{\partial}{\partial a_j}\mathcal{R}_i(m_1, m_2; a_j) + m_i\,, \tag{C.50a}$$

$$\frac{d}{da_j}\left(A|_{a_i=\mathcal{R}_i(m_1,m_2;a_j)}\right) = (1+m_j)\frac{\partial}{\partial a_j}\mathcal{R}_i(m_1, m_2; a_j) + (1+m_i)\,. \tag{C.50b}$$

Substituting $a_i = \mathcal{R}_i(m_1, m_2; a_j)$ into Equation (B.36) and differentiating with respect to country j's unconditional abatement a_j we see that for interior solutions of Equation (B.33) (i.e., whenever $\mathcal{R}_i(m_1, m_2; a_j) > 0$)

$$\begin{aligned}0 = &\left(\frac{\partial}{\partial a_j}\mathcal{R}_i(m_1, m_2; a_j) + m_i\right) B_i''\left(\bar{e}_i - A_i\right)\\ &- \left((1+m_j)\frac{\partial}{\partial a_j}\mathcal{R}_i(m_1, m_2; a_j) + (1+m_i)\right)(1+m_j)\, D_i''\left(\bar{e} - A\right)\,,\end{aligned}$$

which, after rearrangement becomes

$$\frac{\partial}{\partial a_j}\mathcal{R}_i(m_1,m_2;a_j) = -\frac{m_i B_i''(\bar{e}_i - A_i) - (1+m_i)(1+m_j) D_i''(\bar{e} - A)}{B_i''(\bar{e}_i - A_i) - (1+m_j)^2 D_i''(\bar{e} - A)}\Bigg|_{a_i=\mathcal{R}_i(m_1,m_2;a_j)} < 0\,,$$

so country i's best-response function is decreasing country j's unconditional abatement.

Similarly,

$$\begin{aligned}
\frac{d}{dm_i}\left(A_i|_{a_i=\mathcal{R}_i(m_1,m_2;a_j)}\right) &= \frac{\partial}{\partial m_i}\mathcal{R}_i(m_1,m_2;a_j) + a_j\,,\\
\frac{d}{dm_j}\left(A_i|_{a_i=\mathcal{R}_i(m_1,m_2;a_j)}\right) &= \frac{\partial}{\partial m_j}\mathcal{R}_i(m_1,m_2;a_j)\,,\\
\frac{d}{dm_i}\left(A|_{a_i=\mathcal{R}_i(m_1,m_2;a_j)}\right) &= (1+m_j)\frac{\partial}{\partial m_i}\mathcal{R}_i(m_1,m_2;a_j) + a_j\,,\\
\frac{d}{dm_j}\left(A|_{a_i=\mathcal{R}_i(m_1,m_2;a_j)}\right) &= (1+m_j)\frac{\partial}{\partial m_j}\mathcal{R}_i(m_1,m_2;a_j) + \mathcal{R}_i(m_1,m_2;a_j)\,.
\end{aligned}$$

Substituting $a_i = \mathcal{R}_i(m_1,m_2;a_j)$ into Equation (B.36) and differentiating with respect to the matching factors m_i and m_j, we see that interior solutions of Equation (B.33) satisfy

$$\begin{aligned}
0 &= \left(\frac{\partial}{\partial m_i}\mathcal{R}_i(m_1,m_2;a_j) + a_j\right) B_i''(\bar{e}_i - A_i)\\
&\quad - \left((1+m_j)\frac{\partial}{\partial m_i}\mathcal{R}_i(m_1,m_2;a_j) + a_j\right)(1+m_j) D_i''(\bar{e} - A)\,,\\
0 &= \left(\frac{\partial}{\partial m_j}\mathcal{R}_i(m_1,m_2;a_j)\right) B_i''(\bar{e}_i - A_i) + D_i'(\bar{e} - A)\\
&\quad - \left((1+m_j)\frac{\partial}{\partial m_j}\mathcal{R}_i(m_1,m_2;a_j) + \mathcal{R}_i(m_1,m_2;a_j)\right)(1+m_j) D_i''(\bar{e} - A)\,,
\end{aligned}$$

and hence,

$$\begin{aligned}
\frac{\partial}{\partial m_i}\mathcal{R}_i(m_1,m_2;a_j) &= -a_j\frac{B_i''(\bar{e}_i - A_i) - (1+m_j) D_i''(\bar{e} - A)}{B_i''(\bar{e}_i - A_i) - (1+m_j)^2 D_i''(\bar{e} - A)}\Bigg|_{a_i=\mathcal{R}_i(m_1,m_2;a_j)} < 0\,,\\
\frac{\partial}{\partial m_j}\mathcal{R}_i(m_1,m_2;a_j) &= -\frac{D_i'(\bar{e} - A) - (1+m_j) D_i''(\bar{e} - A)\,a_i}{B_i''(\bar{e}_i - A_i) - (1+m_j)^2 D_i''(\bar{e} - A)}\Bigg|_{a_i=\mathcal{R}_i(m_1,m_2;a_j)}\,.
\end{aligned}$$

C.2.3 Case three: continuous differentiability of $\mathcal{R}_i(\vec{p})$ for $\vec{p} \in \partial\,\mathbb{R}^3_{>0}$ such that $\mathcal{R}_i(\vec{p}) > 0$

In the MCG, the matching factors m_i and m_j, and the unconditional abatement levels a_i and a_j, are assumed to be non-negative. However, the payoff function Π_i is well-defined for any $(m_1, m_2; a_1, a_2) \in \mathbb{R}^4$. For the remainder of this proof, we allow the arguments of Π_i to take on negative values, as this considerably simplifies our proof.

Suppose that $\vec{p} \in \partial\,\mathbb{R}^3_{\geq 0}$ satisfies $\mathcal{R}_i(\vec{p}) > 0$. From Lemma B.1, $a_i = \mathcal{R}_i(\vec{p})$ solves the equation

$$\frac{\partial}{\partial a_i}\Pi_i(\vec{p}; a_i) = 0\,,$$

so $m_j > 0$. Thus, because $\vec{p} \in \partial\,\mathbb{R}^3_{\geq 0}$, $m_i = 0$ or $a_j = 0$ (or both). $\frac{\partial}{\partial a_i}\Pi_i$ has continuous partial derivatives in its four arguments (in all of $\mathbb{R}^4$), and using Equation (C.47), there is a neighbourhood of $\left(\vec{p}; \mathcal{R}_i(\vec{p})\right)$ in which

$$\frac{\partial^2}{\partial {a_i}^2}\Pi_i(\vec{p}; a_i) < 0\,,$$

(all that is required for this to hold is that $m_i > -1$). It follows from the implicit function theorem [6] that there is a neighbourhood of $\vec{p}$ in which $\mathcal{R}_i(\vec{p})$ is defined implicitly by Equation (B.36) and continuously differentiable with respect to its arguments, and the formulae for the derivatives of $\mathcal{R}_i(\vec{p})$ obtained in Appendix C.2.2 remain valid (and in particular, hold for the one-sided derivatives of $\mathcal{R}_i(\vec{p})$).

C.3 Slope of the best-response difference, $\Delta\mathcal{R}$ (Proposition B.6)

Suppose that $m_i > 0$ for $i = 1, 2$. Then from Lemma B.2, $\frac{\partial}{\partial a_1}\mathcal{R}_2(m_1, m_2; a_1)$ and $\frac{\partial}{\partial a_2}\mathcal{R}_1(m_1, m_2; a_2)$ are continuously differentiable for all $a_1 \in (0, a_{2,1}^{\text{int}})$ and $a_2 \in (0, a_{1,2}^{\text{int}})$, and satisfy

$$\frac{\partial}{\partial a_1}\mathcal{R}_2(m_1, m_2; a_1) = -\frac{m_2 B_2''(\bar{e}_2 - A_2) - (1+m_2)(1+m_1) D_2''(\bar{e} - A)}{B_2''(\bar{e}_2 - A_2) - (1+m_1)^2 D_2''(\bar{e} - A)}\Bigg|_{a_2 = \mathcal{R}_2(m_1, m_2; a_1)} < 0\,,$$

$$\frac{\partial}{\partial a_2}\mathcal{R}_1(m_1, m_2; a_2) = -\frac{m_1 B_1''(\bar{e}_1 - A_1) - (1+m_1)(1+m_2) D_1''(\bar{e} - A)}{B_1''(\bar{e}_1 - A_1) - (1+m_2)^2 D_1''(\bar{e} - A)}\Bigg|_{a_1 = \mathcal{R}_1(m_1, m_2; a_2)} < 0\,.$$

From the inverse function theorem [6], $\mathcal{R}_1^{-1}(m_1, m_2; a_1)$ is locally invertible and continuously differentiable for all $a_1 \in (0, a_{1,1}^{\text{int}})$. Moreover, if $a_1 = \mathcal{R}_1(m_1, m_2; a_2)$, then $a_2 = \mathcal{R}_1^{-1}(m_1, m_2; a_1)$ and

$$\begin{aligned}
\frac{\partial}{\partial a_1}\mathcal{R}_1^{-1}(m_1, m_2; a_1) &= \frac{1}{\frac{\partial}{\partial a_2}\mathcal{R}_1(m_1, m_2; a_2)}\Bigg|_{a_2 = \mathcal{R}_1^{-1}(m_1, m_2; a_1)} \\
&= -\frac{B_1''(\bar{e}_1 - A_1) - (1+m_2)^2 D_1''(\bar{e} - A)}{m_1 B_1''(\bar{e}_1 - A_1) - (1+m_1)(1+m_2) D_1''(\bar{e} - A)}\Bigg|_{a_2 = \mathcal{R}_1^{-1}(m_1, m_2; a_1)} < 0\,.
\end{aligned}$$

Thus, $\Delta\mathcal{R}(m_1, m_2; a_1)$ is continuously differentiable with respect to a_1 for all $a_1 \in \big(0, \min\{a_{1,1}^{\text{int}}, a_{2,1}^{\text{int}}\}\big)$, and

$$\begin{aligned}
\frac{\partial}{\partial a_1}\Delta\mathcal{R}(m_1, m_2; a_1) &= \frac{\partial}{\partial a_1}\mathcal{R}_2(m_1, m_2; a_1) - \frac{\partial}{\partial a_1}\mathcal{R}_1^{-1}(m_1, m_2; a_1)\,, \\
&= -\frac{m_2 B_2''(\bar{e}_2 - A_2) - (1+m_2)(1+m_1) D_2''(\bar{e} - A)}{B_2''(\bar{e}_2 - A_2) - (1+m_1)^2 D_2''(\bar{e} - A)}\Bigg|_{a_2 = \mathcal{R}_2(m_1, m_2; a_1)} \\
&\quad + \frac{B_1''(\bar{e}_1 - A_1) - (1+m_2)^2 D_1''(\bar{e} - A)}{m_1 B_1''(\bar{e}_1 - A_1) - (1+m_1)(1+m_2) D_1''(\bar{e} - A)}\Bigg|_{a_2 = \mathcal{R}_1^{-1}(m_1, m_2; a_1)}\,.
\end{aligned}$$

At an interior Nash equilibrium (a_1^*, a_2^*), $a_i^* = \mathcal{R}_i(m_1, m_2; a_j^*) > 0$ for $(i, j) = (1, 2)$ and $(2, 1)$. Thus

$$\mathcal{R}_1^{-1}(m_1, m_2; a_1^*) = a_2^* = \mathcal{R}_2(m_1, m_2; a_1^*)\,.$$

Consequently,

$$\begin{aligned}
\frac{\partial}{\partial a_1}\Delta\mathcal{R}(m_1, m_2; a_1^*) = \Bigg(&-\frac{m_2 B_2''(\bar{e}_2 - A_2) - (1+m_2)(1+m_1) D_2''(\bar{e} - A)}{B_2''(\bar{e}_2 - A_2) - (1+m_1)^2 D_2''(\bar{e} - A)} \\
&+ \frac{B_1''(\bar{e}_1 - A_1) - (1+m_2)^2 D_1''(\bar{e} - A)}{m_1 B_1''(\bar{e}_1 - A_1) - (1+m_1)(1+m_2) D_1''(\bar{e} - A)}\Bigg)\Bigg|_{\substack{a_1 = a_1^* \\ a_2 = a_2^*}}\,.
\end{aligned}$$

It follows that

$$\frac{\partial}{\partial a_1}\Delta\mathcal{R} = -\frac{N_1}{D_1} + \frac{N_2}{D_2} = \frac{N_2 D_1 - N_1 D_2}{D_1 D_2}\,,$$

where

$$\begin{aligned}
N_1 &= m_2 B_2'' (\bar{e}_2 - A_2) - (1+m_2)(1+m_1) D_2'' (\bar{e} - A) \ , \\
D_1 &= B_2'' (\bar{e}_2 - A_2) - (1+m_1)^2 D_2'' (\bar{e} - A) \ , \\
N_2 &= B_1'' (\bar{e}_1 - A_1) - (1+m_2)^2 D_1'' (\bar{e} - A) \ , \\
D_2 &= m_1 B_1'' (\bar{e}_1 - A_1) - (1+m_1)(1+m_2) D_1'' (\bar{e} - A) \ ,
\end{aligned}$$

and we implicitly evaluate all terms at the Nash equilibrium $(a_1, a_2) = (a_1^*, a_2^*)$.

Simplifying, we have

$$\begin{aligned}
N_2 D_1 = \quad & B_1'' (\bar{e}_1 - A_1) B_2'' (\bar{e}_2 - A_2) \\
& - (1+m_1)^2 B_1'' (\bar{e}_1 - A_1) D_2'' (\bar{e} - A) \\
& - (1+m_2)^2 B_2'' (\bar{e}_2 - A_2) D_1'' (\bar{e} - A) \\
& + (1+m_1)^2 (1+m_2)^2 D_1'' (\bar{e} - A) D_2'' (\bar{e} - A) \ .
\end{aligned}$$

and,

$$\begin{aligned}
N_1 D_2 = \quad & m_1 m_2 B_1'' (\bar{e}_1 - A_1) B_2'' (\bar{e}_2 - A_2) \\
& - m_1 (1+m_1)(1+m_2) B_1'' (\bar{e}_1 - A_1) D_2'' (\bar{e} - A) \\
& - m_2 (1+m_1)(1+m_2) B_2'' (\bar{e}_2 - A_2) D_1'' (\bar{e} - A) \\
& + (1+m_1)^2 (1+m_2)^2 D_1'' (\bar{e} - A) D_2'' (\bar{e} - A) \ ,
\end{aligned}$$

so

$$\begin{aligned}
N_2 D_1 - N_1 D_2 = \quad & (1 - m_1 m_2) B_1'' (\bar{e}_1 - A_1) B_2'' (\bar{e}_2 - A_2) \\
& - \big((1+m_1)^2 - m_1 (1+m_1)(1+m_2) \big) B_1'' (\bar{e}_1 - A_1) D_2'' (\bar{e} - A) \\
& - \big((1+m_2)^2 - m_2 (1+m_1)(1+m_2) \big) B_2'' (\bar{e}_2 - A_2) D_1'' (\bar{e} - A) \\
= \quad & (1 - m_1 m_2) B_1'' (\bar{e}_1 - A_1) B_2'' (\bar{e}_2 - A_2) \\
& - \big(1 + m_1 - m_1 (1+m_2) \big) (1+m_1) B_1'' (\bar{e}_1 - A_1) D_2'' (\bar{e} - A) \\
& - \big(1 + m_2 - m_2 (1+m_1) \big) (1+m_2) B_2'' (\bar{e}_2 - A_2) D_1'' (\bar{e} - A) \ ,
\end{aligned}$$

or

$$\begin{aligned}
N_2 D_1 - N_1 D_2 = \big(1 - m_1 m_2 \big) \Big[& B_1'' (\bar{e}_1 - A_1) B_2'' (\bar{e}_2 - A_2) - (1+m_1) B_1'' (\bar{e}_1 - A_1) D_2'' (\bar{e} - A) \\
& - (1+m_2) B_2'' (\bar{e}_2 - A_2) D_1'' (\bar{e} - A) \Big] \ .
\end{aligned}$$

Thus,

$$\begin{aligned}
& \frac{\partial}{\partial a_1} \Delta \mathcal{R} = \\
& \quad \big(1 - m_1 m_2 \big) \times \Big[B_1'' (\bar{e}_1 - A_1) B_2'' (\bar{e}_2 - A_2) - (1+m_1) B_1'' (\bar{e}_1 - A_1) D_2'' (\bar{e} - A) \\
& \qquad - (1+m_2) B_2'' (\bar{e}_2 - A_2) D_1'' (\bar{e} - A) \Big] \\
& \quad \Big/ \Big[\Big(B_2'' (\bar{e}_2 - A_2) - (1+m_1)^2 D_2'' (\bar{e} - A) \Big) \\
& \qquad \times \Big(m_1 B_1'' (\bar{e}_1 - A_1) - (1+m_1)(1+m_2) D_1'' (\bar{e} - A) \Big) \Big] \ . \qquad \text{(C.51)}
\end{aligned}$$

Note that because the terms in square brackets are positive, the signs of $\frac{\partial}{\partial a_1} \Delta \mathcal{R}(m_1, m_2; a_1^*)$ and $1 - m_1 m_2$ are identical.

Lastly, if $(a_{1,1}^{\text{int}}, 0)$ is an equilibrium, since $\frac{\partial}{\partial a_1} \mathcal{R}_2(m_1, m_2; a_1)$ and $\frac{\partial}{\partial a_1} \mathcal{R}_1^{-1}(m_1, m_2; a_1)$ are continuous in a left-neighbourhood of $a_1 = a_{1,1}^{\text{int}}$ and each approaches a finite limit as $a_1 \to a_{1,1}^{\text{int}}$, the derivation leading to Equation (C.51) still holds, and hence the right-sided derivative of $\Delta \mathcal{R}(m_1, m_2; a_1)$ at $a_1 = a_{1,1}^{\text{int}}$ exists and is equal to the right hand side of Equation (C.51). An analogous statement holds if $(0, a_{2,2}^{\text{int}})$ is an equilibrium.

C.4 Nash equilibria when no interior equilibrium exists

C.4.1 The baseline scenario (proof of Proposition B.7)

If $m_1 = m_2 = 0$ then, Lemma B.1, implies that $\frac{\partial}{\partial a_i}\Pi_i \leq 0$ for $i = 1$ and $i = 2$, so both best-response functions are identically 0 (i.e., both countries are better off not abating unconditionally, regardless of the other country's unconditional abatement). The unique Nash equilibrium is the $(a_1, a_2) = (0, 0)$ (i.e., baseline).

Conversely, Lemma B.1 implies that if $\mathcal{R}_i(m_1, m_2; 0) = 0$ then $\mathcal{R}_i(m_1, m_2; a_j) = 0$ for all $a_j > 0$, and hence $m_i = 0$. Consequently, if $(a_i, a_j) = (0, 0)$ is a Nash equilibrium, then $m_1 = m_2 = 0$.

C.4.2 If one country will not match, the other will not abate unconditionally (proof of Proposition B.8)

If $m_i = 0$ and $m_j > 0$, then by Lemma B.1, $\mathcal{R}_j(m_1, m_2; a_i) \equiv 0$, and there is $a^{\text{int}}_{i,j} > 0$ such that $\mathcal{R}_i(m_1, m_2; a_j) > 0$ for all a_j satisfying $0 \leq a_j < a^{\text{int}}_{i,j}$, and $\mathcal{R}_i(m_1, m_2; a_j) = 0$ for all $a_j \geq a^{\text{int}}_{i,j}$. Thus, $(a^*_i, a^*_j) = (a^{\text{int}}_{i,i}, 0)$ is the unique point at which the best-response functions intersect (Equation (B.35)), and so the only Nash equilibrium.

C.4.3 Nash equilibria when both countries match, but no interior equilibrium exists (proof of Proposition B.9)

Suppose that $m_i > 0$ for both $i = 1$ and 2, but not interior equilibria exist. Lemma B.1 implies that neither country i's best-response function is identically 0: there exists $a^{\text{int}}_{i,j} > 0$ such that $\mathcal{R}_i(m_1, m_2; a_j) > 0$ for all a_j satisfying $0 \leq a_j < a^{\text{int}}_{i,j}$, and $\mathcal{R}_i(m_1, m_2; a_j) = 0$ for all $a_j \geq a^{\text{int}}_{i,j}$. Since there are no interior Nash equilibria, the best-response functions do not intersect at any $(a_i, a_j) \in \mathbb{R}^2_{>0}$. Because the best-response functions are continuous, this implies that one country's best-response function must be higher than the other's in the interior of the quadrant of positive unconditional abatements: either $\mathcal{R}_1(m_1, m_2; a_2)$ is higher than $\mathcal{R}_2(m_1, m_2; a_1)$ in that quadrant, in which case

$$\begin{cases} a^{\text{int}}_{1,1} & \geq a^{\text{int}}_{2,1}\,, \\ a^{\text{int}}_{1,2} & \geq a^{\text{int}}_{2,2}\,, \end{cases} \tag{C.52a}$$

or $\mathcal{R}_2(m_1, m_2; a_1)$ is higher than $\mathcal{R}_1(m_1, m_2; a_2)$ there, in which case

$$\begin{cases} a^{\text{int}}_{2,1} & \geq a^{\text{int}}_{1,1}\,, \\ a^{\text{int}}_{2,2} & \geq a^{\text{int}}_{1,2}\,. \end{cases} \tag{C.52b}$$

It follows that the only possible Nash equilibria are $(a^{\text{int}}_{1,1}, 0)$ and $(0, a^{\text{int}}_{2,2})$ (Remark B.5).

We now examine in more detail the combinations of strict inequalities and equalities allowed for in Equation (C.52). First, observe that if both inequalities in Equation (C.52a) are made strict, that is,

$$\begin{cases} a^{\text{int}}_{1,1} & > a^{\text{int}}_{2,1}\,, \\ a^{\text{int}}_{1,2} & > a^{\text{int}}_{2,2}\,, \end{cases} \tag{C.53}$$

then from Remark B.5 $(a^{\text{int}}_{1,1}, 0)$ is the unique Nash equilibrium, and similarly, if

$$\begin{cases} a^{\text{int}}_{2,1} & > a^{\text{int}}_{1,1}\,, \\ a^{\text{int}}_{2,2} & > a^{\text{int}}_{1,2}\,, \end{cases} \tag{C.54}$$

then $(0, a^{\text{int}}_{2,2})$ is the unique Nash equilibrium. Note that Equations (C.53) and (C.54) are possible if $m_1 m_2 < 1$, $m_1 m_2 = 1$ or $m_1 m_2 > 1$.

Next, we consider the cases in which the best-response functions intersect one another at their intercepts with at least one of the axes, that is, either $a^{\text{int}}_{2,2} = a^{\text{int}}_{1,2}$ or $a^{\text{int}}_{1,1} = a^{\text{int}}_{2,1}$.

Observe that if $m_1 m_2 = 1$, then using Lemma B.11, if $a^{\text{int}}_{2,2} = a^{\text{int}}_{1,2}$ or $a^{\text{int}}_{1,1} = a^{\text{int}}_{2,1}$, then the two best-response functions overlap in the interior of the quadrant of positive unconditional abatements, contradicting our assumption that there are no interior equilibria. Thus, if $m_1 m_2 = 1$ then the only possibilities are Equations (C.53) or (C.54).

Now suppose that $m_1 m_2 \neq 1$.

- If $a^{\text{int}}_{1,1} = a^{\text{int}}_{2,1}$, then $\Delta\mathcal{R}(m_1, m_2; a^{\text{int}}_{1,1}) = 0$ and from Proposition B.6,

$$\lim_{a_1 \to (a^{\text{int}}_{1,1})^+} \frac{\partial}{\partial a_1} \Delta\mathcal{R} > 0 \quad \text{if } m_1 m_2 < 1\,,$$
$$\lim_{a_1 \to (a^{\text{int}}_{1,1})^+} \frac{\partial}{\partial a_1} \Delta\mathcal{R} < 0 \quad \text{if } m_1 m_2 > 1\,,$$

  Because there are no interior equilibria (by assumption), $\Delta\mathcal{R}$ has a constant sign over $(0, a^{\text{int}}_{1,1})$:

$$\begin{cases} \Delta\mathcal{R} < 0 \quad \forall a_1 \in (0, a^{\text{int}}_{1,1}) & \text{if } m_1 m_2 < 1\,, \\ \Delta\mathcal{R} > 0 \quad \forall a_1 \in (0, a^{\text{int}}_{1,1}) & \text{if } m_1 m_2 > 1\,. \end{cases}$$

  In particular,

$$\begin{cases} a^{\text{int}}_{1,2} & > a^{\text{int}}_{2,2} & \text{if } m_1 m_2 < 1\,, \\ a^{\text{int}}_{1,2} & < a^{\text{int}}_{2,2} & \text{if } m_1 m_2 > 1\,. \end{cases}$$

- Similarly, if $a^{\text{int}}_{2,2} = a^{\text{int}}_{1,2}$, then

$$\begin{cases} \Delta\mathcal{R} & > 0 \quad \forall a_1 \in (0, a^{\text{int}}_{1,1}) & \text{if } m_1 m_2 < 1\,, \\ \Delta\mathcal{R} & < 0 \quad \forall a_1 \in (0, a^{\text{int}}_{1,1}) & \text{if } m_1 m_2 > 1\,, \end{cases}$$

  and

$$\begin{cases} a^{\text{int}}_{1,1} & < a^{\text{int}}_{2,1} & \text{if } m_1 m_2 < 1\,, \\ a^{\text{int}}_{1,1} & > a^{\text{int}}_{2,1} & \text{if } m_1 m_2 > 1\,. \end{cases}$$

To sum up, if $m_i > 0$ for $i = 1$ and 2, and there are no interior equilibria, then using Remark B.5:

If $m_1 m_2 < 1$ then either

$$\begin{cases} a^{\text{int}}_{1,1} & \geq a^{\text{int}}_{2,1}\,, \\ a^{\text{int}}_{1,2} & > a^{\text{int}}_{2,2}\,, \end{cases} \quad \text{or} \quad \begin{cases} a^{\text{int}}_{1,1} & < a^{\text{int}}_{2,1}\,, \\ a^{\text{int}}_{1,2} & \leq a^{\text{int}}_{2,2}\,, \end{cases}$$

in which case the unique Nash equilibrium is $(a^{\text{int}}_{1,1}, 0)$ or $(0, a^{\text{int}}_{2,2})$, respectively.

If $m_1 m_2 = 1$ then either

$$\begin{cases} a^{\text{int}}_{1,1} & > a^{\text{int}}_{2,1}\,, \\ a^{\text{int}}_{1,2} & > a^{\text{int}}_{2,2}\,, \end{cases} \quad \text{or} \quad \begin{cases} a^{\text{int}}_{1,1} & < a^{\text{int}}_{2,1}\,, \\ a^{\text{int}}_{1,2} & < a^{\text{int}}_{2,2}\,, \end{cases} \tag{C.55}$$

in which case the unique Nash equilibrium is $(a^{\text{int}}_{1,1}, 0)$ or $(0, a^{\text{int}}_{2,2})$, respectively.

If $m_1 m_2 > 1$ then either Equation (C.55) holds (with Nash equilibria determined identically to the case $m_1 m_2 = 1$), or

$$\begin{cases} a^{\text{int}}_{1,1} & = a^{\text{int}}_{2,1}\,, \\ a^{\text{int}}_{1,2} & < a^{\text{int}}_{2,2}\,, \end{cases} \quad \text{or} \quad \begin{cases} a^{\text{int}}_{1,1} & > a^{\text{int}}_{2,1}\,, \\ a^{\text{int}}_{1,2} & = a^{\text{int}}_{2,2}\,, \end{cases}$$

and the Nash equilibria are both $(a^{\text{int}}_{1,1}, 0)$ and $(0, a^{\text{int}}_{2,2})$.

C.5 Nash equilibria when no interior equilibrium exists and matching factors are not reciprocal (proof of Proposition B.10)

To prove Proposition B.10, we require the following lemma:

Lemma C.2 (Sufficient condition for a unique zero crossing)**.** *Let $f : I \to \mathbb{R}$ be a differentiable function defined on a nontrivial open interval $I = (a, b) \subseteq \mathbb{R}$ ($a, b \in \overline{\mathbb{R}}$ and $a < b$). If $f(x) = 0$ implies $f'(x) > 0$, then the equation $f(x) = 0$ has at most one solution in I.*

Proof. Suppose that $x_1, x_2 \in I$ satisfy $f(x_1) = f(x_2) = 0$ and that $x_1 < x_2$. Let

$$O = \left\{x \in (x_1, x_2] \,\middle|\, f(x) = 0\right\},$$

and $x_0 = \inf O$ (note that $O \neq \phi$ because $x_2 \in O$). Since f is continuous, $f(x_0) = 0$. Because $f'(x_1) > 0$ $x_0 > x_1$. Since f is continuous and is never 0 on the interval (x_1, x_0), it has constant sign on it. But $f'(x_1) > 0$ implies that f is positive on (x_1, x_0), and $f'(x_0) > 0$ implies that f is negative on this interval—a contradiction. Thus, $f(x) = 0$ has at most one solution in I. □

We now proceed with the proof of Proposition B.10: If $m_1 m_2 < 1$, and (a_1^*, a_2^*) is an interior Nash equilibrium, then by Proposition B.6,

$$\frac{\partial}{\partial a_1} \Delta\mathcal{R}(m_1, m_2; a_1^*) > 0\,,$$

that is, at $a_1 = a_1^*$, $\Delta\mathcal{R}(m_1, m_2; a_1)$ increases through 0 as a function of a_1. It follows immediately from Lemma C.2 above that no other interior Nash equilibrium exists.

An analogous argument shows that at most one interior Nash equilibrium exists when $m_1 m_2 > 1$ (the difference being that in this case, $\frac{\partial}{\partial a_1} \Delta\mathcal{R}(m_1, m_2; a_1) < 0$ at an interior Nash equilibrium, and we can apply Lemma C.2 to $-\Delta\mathcal{R}(m_1, m_2; a_1)$).

Thus:

If $m_1 m_2 < 1$ then because $\Delta\mathcal{R}(m_1, m_2; a_1)$ increases through 0 as a function of a_1,

$$\Delta\mathcal{R}(m_1, m_2; a_1) < 0 \quad \text{for all } a_1 \in (0, a_1^*)\,, \tag{C.56a}$$

$$\Delta\mathcal{R}(m_1, m_2; a_1) > 0 \quad \text{for all } a_1 \in (a_1^*, a_{1,1}^{\text{int}})\,. \tag{C.56b}$$

and by continuity of $\Delta\mathcal{R}(m_1, m_2; a_1)$,

$$a_{1,1}^{\text{int}} \leq a_{2,1}^{\text{int}}\,, \tag{C.57a}$$

$$a_{1,2}^{\text{int}} \geq a_{2,2}^{\text{int}}\,. \tag{C.57b}$$

In fact, we show below that these inequalities are strict, so no edge equilibria exist. Thus, (a_1^*, a_2^*) is the unique Nash equilibrium.

To see that Equation (C.57) can be made strict, that is,

$$a_{1,1}^{\text{int}} < a_{2,1}^{\text{int}}\,,$$
$$a_{1,2}^{\text{int}} > a_{2,2}^{\text{int}}\,,$$

(or equivalently, that $\Delta\mathcal{R}(m_1, m_2; 0) < 0$ and $\Delta\mathcal{R}(m_1, m_2; a_{1,1}^{\text{int}}) > 0$) we exploit the existence of the right- and left-sided limits of $\frac{\partial}{\partial a_1} \Delta\mathcal{R}(m_1, m_2; a_1)$ at $a_{1,1}^{\text{int}}$ and $a_1 = 0$ (respectively).

Suppose, in order to derive a contradiction, that $\Delta\mathcal{R}(m_1, m_2; a_{1,1}^{\text{int}}) = 0$, so that $(a_{1,1}^{\text{int}}, 0)$ is a Nash equilibrium. From Proposition B.6,

$$\lim_{a_1 \to (a_{1,1}^{\text{int}})^-} \frac{\partial}{\partial a_1} \Delta\mathcal{R}(m_1, m_2; a_1) > 0\,.$$

Since $\Delta\mathcal{R}(m_1, m_2; a_{1,1}^{\text{int}}) = 0$ by assumption, there is a left-neighbourhood of $a_1 = a_{1,1}^{\text{int}}$ in which

$$\Delta\mathcal{R}(m_1, m_2; a_1) < 0\,,$$

contradicting Equation (C.56b). Thus, $\Delta\mathcal{R}(m_1, m_2; a_{1,1}^{\text{int}}) > 0$ as required.

Analogously, if i.e., $(0, a_{2,2}^{\text{int}})$ is a Nash equilibrium, then Proposition B.6 implies

$$\lim_{a_1 \to 0^+} \frac{\partial}{\partial a_1} \Delta\mathcal{R}(m_1, m_2; a_1) > 0\,,$$

and because $\Delta\mathcal{R}(m_1, m_2; 0) = 0$, $\Delta\mathcal{R}(m_1, m_2; a_1) > 0$ in a right-neighbourhood of $a_1 = 0$, contradicting Equation (C.56a). Thus, $\Delta\mathcal{R}(m_1, m_2; a_{1,1}^{\text{int}}) < 0$.

If $m_1 m_2 > 1$ then because $\Delta\mathcal{R}(m_1, m_2; a_1)$ decreases through 0 as a function of a_1,

$$\begin{aligned} \Delta\mathcal{R}(m_1, m_2; a_1) > 0 \quad &\text{for all } a_1 \in (0, a_1^*)\,, \\ \Delta\mathcal{R}(m_1, m_2; a_1) < 0 \quad &\text{for all } a_1 \in (a_1^*, \min\{a_{1,1}^{\text{int}}, a_{2,1}^{\text{int}}\})\,, \end{aligned}$$

and by continuity of $\Delta\mathcal{R}(m_1, m_2; a_1)$, $a_{2,2}^{\text{int}} \geq a_{1,2}^{\text{int}}$ and $a_{1,1}^{\text{int}} \geq a_{2,1}^{\text{int}}$. It follows from Remark B.5 that two edge equilibria exist (in addition to the interior equilibrium[8]): $(0, a_{2,2}^{\text{int}})$ and $(a_{1,1}^{\text{int}}, 0)$.

An argument similar to the one used to show that the inequalities in Equation (C.57) can be made strict also establishes that $a_{1,1}^{\text{int}} > a_{2,1}^{\text{int}}$ and $a_{2,2}^{\text{int}} > a_{1,2}^{\text{int}}$.

C.6 Proofs for reciprocal matching factors

C.6.1 Best-responses for reciprocal matching factors (proof of Lemma B.11)

If $(m_1, m_2) \in \mathcal{M}_{\text{r}}$, then $m_1 m_2 = 1$ and $m_i > 0$. Thus, $a_{i,j}^{\text{int}}$ and $a_{i,i}^{\text{int}} > 0$ (see Lemma B.1 and Remark B.3) and for $0 \leq a_j < a_{i,j}^{\text{int}}$, Equation (B.38) becomes

$$\begin{aligned} \frac{\partial}{\partial a_j} \mathcal{R}_i(m_1, m_2; a_j) &= -\frac{(1/m_j)\, B_i''\,(\bar{e}_i - A_i) - (1 + 1/m_j)\,(1 + m_j)\, D_i''\,(\bar{e} - A)}{B_i''\,(\bar{e}_i - A_i) - (1 + m_j)^2\, D_i''\,(\bar{e} - A)} \\ &= -\frac{1}{m_j} \frac{B_i''\,(\bar{e}_i - A_i) - (1 + m_j)^2\, D_i''\,(\bar{e} - A)}{B_i''\,(\bar{e}_i - A_i) - (1 + m_j)^2\, D_i''\,(\bar{e} - A)} = -m_i\,, \end{aligned} \tag{C.58}$$

so $\mathcal{R}_i(m_1, m_2; a_j)$ is linear in the range $0 \leq a_j \leq a_{i,j}^{\text{int}}$; for $a_j > a_{i,j}^{\text{int}}$, $\mathcal{R}_i(m_1, m_2; a_j) = 0$, justifying Equation (B.41).

By definition, $\mathcal{R}_i(m_1, m_2; a_{i,j}^{\text{int}}) = 0$, so substituting $a_j = a_{i,j}^{\text{int}}$ in Equation (B.41) gives $a_{i,i}^{\text{int}} = m_i a_{i,j}^{\text{int}}$.

Since $m_i \neq 0$ (because $m_1 m_2 = 1$), country i's best-response function is decreasing in the range $0 \leq a_j \leq a_{i,j}^{\text{int}}$, and thus has an inverse

$$\mathcal{R}_i^{-1}(m_1, m_2; a_i) = -\frac{1}{m_i} a_i + a_{i,j}^{\text{int}} = -m_j a_i + a_{i,j}^{\text{int}}\,, \tag{C.59}$$

defined for $0 \leq a_i \leq a_{i,i}^{\text{int}}$.

C.6.2 Uniqueness of reciprocal matching factors yielding interior Nash equilibria (proof of Lemma B.12)

Consider the *vertical* distance between the two best-response functions in the a_1–a_2 plane when the matching factors are reciprocal, i.e.,

$$\Delta\mathcal{R}(m_1, 1/m_1; a_1) = \mathcal{R}_2(m_1, 1/m_1; a_1) - \mathcal{R}_1^{-1}(m_1, 1/m_1; a_1)\,, \tag{C.60}$$

[8] In this case, the interior equilibrium is unstable and the two edge equilibria are stable in the sense of "cobweb stability" [7, p. 287].

where

$$a_1 \in I = \big[0, \min\{a_{1,1}^{\text{int}}, a_{2,1}^{\text{int}}\}\big] \ .$$

We wish to analyze how the distance between the best-response functions changes with the matching factors (constrained to being reciprocal). From Lemma B.11, $\mathcal{R}_2(m_1, 1/m_1; a_1)$ and $\mathcal{R}_1^{-1}(m_1, 1/m_1; a_1)$ are positive, linear and parallel for all $a_1 \in I$, so the distance $\Delta\mathcal{R}(m_1, 1/m_1; a_1)$ is independent of $a_1 \in I$. More formally, using Lemma B.11, we see that for $a_1 \in I$, Equation (C.60) becomes

$$\Delta\mathcal{R}(m_1, 1/m_1; a_1) = \big(-m_2 a_1 + a_{2,2}^{\text{int}}\big) - \big(-m_2 a_1 + a_{1,2}^{\text{int}}\big) = a_{2,2}^{\text{int}} - a_{1,2}^{\text{int}} \,. \tag{C.61}$$

Hence, $\Delta\mathcal{R}(m_1, 1/m_1; a_1)$ does not depend on a_1, and we can (with minor abuse of notation) write $\Delta\mathcal{R}(m_1)$; note that since the matching factors are reciprocal, $a_{2,2}^{\text{int}}$, $a_{1,2}^{\text{int}}$ and $\Delta\mathcal{R}(m_1, 1/m_1; a_1)$ can be written as functions of either one of the matching factors.

In order to calculate $\frac{\mathrm{d}}{\mathrm{d}m_1}\Delta\mathcal{R}$, we must calculate the derivatives of $a_{2,2}^{\text{int}}$ and $a_{1,2}^{\text{int}}$ with respect to m_1. Recall from Remark B.3 that $a_{i,i}^{\text{int}}$, is the unique solution of $\frac{\partial}{\partial a_i}\Pi_i\Big|_{a_j=0} = 0$, that is,

$$-B_i'\left(\bar{e}_i - a_i\right) + (1+m_j) D_i'\big(\bar{e} - (1+m_j)a_i\big) = 0\,, \tag{C.62a}$$

and from Lemma B.1, $a_{i,j}^{\text{int}}$, is the unique solution of $\frac{\partial}{\partial a_i}\Pi_i\Big|_{a_i=0} = 0$, that is,

$$-B_i'(\bar{e}_i - m_i a_j) + (1+m_j) D_i'\big(\bar{e} - (1+m_i)a_j\big) = 0\,. \tag{C.62b}$$

Taking the derivative of Equation (C.62a) with respect to m_j, keeping $m_1 m_2 = 1$, yields

$$B_i''\left(\bar{e}_i - a_{i,i}^{\text{int}}\right)\frac{\mathrm{d}}{\mathrm{d}m_j}a_{i,i}^{\text{int}} + D_i'\big(\bar{e} - (1+m_j)a_{i,i}^{\text{int}}\big) - (1+m_j)D_i''\big(\bar{e} - (1+m_j)a_{i,i}^{\text{int}}\big)\Big(a_{i,i}^{\text{int}} + (1+m_j)\frac{\mathrm{d}}{\mathrm{d}m_j}a_{i,i}^{\text{int}}\Big) = 0\,,$$

or

$$\frac{\mathrm{d}}{\mathrm{d}m_j}a_{i,i}^{\text{int}} = -\frac{D_i'\big(\bar{e} - (1+m_j)a_{i,i}^{\text{int}}\big) - (1+m_j)D_i''\big(\bar{e} - (1+m_j)a_{i,i}^{\text{int}}\big)a_{i,i}^{\text{int}}}{B_i''\left(\bar{e}_i - a_{i,i}^{\text{int}}\right) - (1+m_j)^2 D_i''\big(\bar{e} - (1+m_j)a_{i,i}^{\text{int}}\big)}\,. \tag{C.63}$$

Similarly, taking the derivative of Equation (C.62b) with respect to m_i (subject to $m_1 m_2 = 1$) yields

$$B_i''\big(\bar{e}_i - m_i a_{i,j}^{\text{int}}\big)\frac{\mathrm{d}}{\mathrm{d}m_i}\Big(m_i a_{i,j}^{\text{int}}\Big) - (1+m_j)D_i''\big(\bar{e} - (1+m_i)a_{i,j}^{\text{int}}\big)\frac{\mathrm{d}}{\mathrm{d}m_i}\big((1+m_i)a_{i,j}^{\text{int}}\big) - \frac{D_i'\big(\bar{e} - (1+m_i)a_{i,j}^{\text{int}}\big)}{m_i^2} = 0\,,$$

which simplifies to

$$\frac{\mathrm{d}}{\mathrm{d}m_i}a_{i,j}^{\text{int}} = \frac{m_j^2 D_i'\big(\bar{e} - (1+m_i)a_{i,j}^{\text{int}}\big) - \Big(B_i''\big(\bar{e}_i - m_i a_{i,j}^{\text{int}}\big) - (1+m_j)D_i''\big(\bar{e} - (1+m_i)a_{i,j}^{\text{int}}\big)\Big)a_{i,j}^{\text{int}}}{m_i B_i''\big(\bar{e}_i - m_i a_{i,j}^{\text{int}}\big) - (1+m_i)(1+m_j)D_i''\big(\bar{e} - (1+m_i)a_{i,j}^{\text{int}}\big)} < 0\,. \tag{C.64}$$

We are interested the derivative of $\Delta\mathcal{R}(m_1)$ at matching factors m_1 for which $\Delta\mathcal{R}(m_1) = 0$. Using the facts that $a_{2,2}^{\text{int}} = a_{1,2}^{\text{int}}$ at such a zero crossing of $\Delta\mathcal{R}$, and that $(1+m_1)(1+m_2) = \frac{(1+m_1)^2}{m_1}$ (because $m_1 m_2 = 1$), Equation (C.64) gives

$$\begin{aligned}\frac{\mathrm{d}}{\mathrm{d}m_1}a_{1,2}^{\text{int}} &= \frac{\frac{1}{m_1^2}D_1'\big(\bar{e} - (1+m_1)a_{2,2}^{\text{int}}\big) - \Big(B_1''\big(\bar{e}_1 - m_1 a_{2,2}^{\text{int}}\big) - \frac{1+m_1}{m_1}D_1''\big(\bar{e} - (1+m_1)a_{2,2}^{\text{int}}\big)\Big)a_{2,2}^{\text{int}}}{m_1 B_1''\big(\bar{e}_1 - m_1 a_{2,2}^{\text{int}}\big) - \frac{(1+m_1)^2}{m_1}D_1''\big(\bar{e} - (1+m_1)a_{2,2}^{\text{int}}\big)} \\ &= \frac{D_1'\big(\bar{e} - (1+m_1)a_{2,2}^{\text{int}}\big) - \Big(m_1^2 B_1''\big(\bar{e}_1 - m_1 a_{2,2}^{\text{int}}\big) - m_1(1+m_1)D_1''\big(\bar{e} - (1+m_1)a_{2,2}^{\text{int}}\big)\Big)a_{2,2}^{\text{int}}}{m_1^3 B_1''\big(\bar{e}_1 - m_1 a_{2,2}^{\text{int}}\big) - m_1(1+m_1)^2 D_1''\big(\bar{e} - (1+m_1)a_{2,2}^{\text{int}}\big)}\,.\end{aligned} \tag{C.65}$$

Using Equations (C.61), (C.63) and (C.65),

$$\begin{aligned}\frac{\mathrm{d}}{\mathrm{d}m_1}\Delta\mathcal{R} &= \frac{\mathrm{d}}{\mathrm{d}m_1}a_{2,2}^{\mathrm{int}} - \frac{\mathrm{d}}{\mathrm{d}m_1}a_{1,2}^{\mathrm{int}} \\ &= -\frac{D_2'\big(\bar{e}-(1+m_1)a_{2,2}^{\mathrm{int}}\big) - (1+m_1)D_2''\big(\bar{e}-(1+m_1)a_{2,2}^{\mathrm{int}}\big)a_{2,2}^{\mathrm{int}}}{B_2''\left(\bar{e}_2 - a_{2,2}^{\mathrm{int}}\right) - (1+m_1)^2 D_2''\big(\bar{e}-(1+m_1)a_{2,2}^{\mathrm{int}}\big)} \\ &\quad - \frac{D_1'\big(\bar{e}-(1+m_1)a_{2,2}^{\mathrm{int}}\big) - \Big(m_1^2 B_1''\big(\bar{e}_1 - m_1 a_{2,2}^{\mathrm{int}}\big) - m_1(1+m_1)D_1''\big(\bar{e}-(1+m_1)a_{2,2}^{\mathrm{int}}\big)\Big)a_{2,2}^{\mathrm{int}}}{m_1^3 B_1''\big(\bar{e}_1 - m_1 a_{2,2}^{\mathrm{int}}\big) - m_1(1+m_1)^2 D_1''\big(\bar{e}-(1+m_1)a_{2,2}^{\mathrm{int}}\big)}\,.\end{aligned} \tag{C.66}$$

To simplify notation, for $i = 1, 2$, let $b_i = B_i''\big(\bar{e}_1 - m_1 a_{2,2}^{\mathrm{int}}\big) < 0$, $d_i = D_i'\big(\bar{e} - (1+m_1)a_{2,2}^{\mathrm{int}}\big) > 0$, and $d_i' = (1+m_1)D_i''\big(\bar{e}-(1+m_1)a_{2,2}^{\mathrm{int}}\big) > 0$. Then, Equation (C.66) becomes

$$\begin{aligned}\frac{\mathrm{d}}{\mathrm{d}m_1}a_{1,2}^{\mathrm{int}} &= -\frac{d_2 - d_2' a_{2,2}^{\mathrm{int}}}{b_2 - (1+m_1)d_2'} - \frac{d_1 - \big(m_1^2 b_1 - m_1 d_1'\big)a_{2,2}^{\mathrm{int}}}{m_1^3 b_1 - m_1(1+m_1)d_1'} \\ &= -\frac{\big(d_2 - d_2' a_{2,2}^{\mathrm{int}}\big)\Big(m_1^3 b_1 - m_1(1+m_1)d_1'\Big) + \Big(d_1 - \big(m_1^2 b_1 - m_1 d_1'\big)a_{2,2}^{\mathrm{int}}\Big)\Big(b_2 - (1+m_1)d_2'\Big)}{\Big(b_2 - (1+m_1)d_2'\Big)\Big(m_1^3 b_1 - m_1(1+m_1)d_1'\Big)} \\ &= \frac{m_1^2 a_{2,2}^{\mathrm{int}} b_1 b_2 - m_1^3 b_1 d_2 - b_2 d_1 + m_1(1+m_1)d_2 d_1' - m_1 a_{2,2}^{\mathrm{int}} b_2 d_1' + (1+m_1)d_1 d_2' - m_1^2 a_{2,2}^{\mathrm{int}} b_1 d_2'}{\Big(b_2 - (1+m_1)d_2'\Big)\Big(m_1^3 b_1 - m_1(1+m_1)d_1'\Big)}\,,\end{aligned}$$

which is positive since each of the summands in the numerator is positive, and the two factors in the denominator are negative. Thus, at a zero crossing of $\Delta\mathcal{R}(m_1)$,

$$\frac{\mathrm{d}}{\mathrm{d}m_1}\Delta\mathcal{R}(m_1) > 0\,. \tag{C.67}$$

Since $\Delta\mathcal{R}(m_1)$ is continuous (indeed, differentiable) when $m_1 > 0$, this implies that $\Delta\mathcal{R}$ can have at most one zero crossing (Lemma C.2), which corresponds to a unique pair of reciprocal matching factors for which the best-response functions overlap.

C.6.3 Existence of reciprocal matching factors for which interior equilibria exist (proof of Lemma B.13)

We have shown that for reciprocal matching factors, the distance between the best-response functions is continuous and must increase with m_1 at a zero crossing of $\Delta\mathcal{R}$ (Equation (C.67)), and that there is at most one such zero crossing (Appendix C.6.2). It follows that there exists $m_1 > 0$ such that $\Delta\mathcal{R}(m_1) = 0$ iff

$$\lim_{m_1 \to 0^+} \operatorname{sign}\Delta\mathcal{R}(m_1) = -1\,,$$

and

$$\lim_{m_1 \to \infty} \operatorname{sign}\Delta\mathcal{R}(m_1) = 1\,.$$

Observe that since $\mathcal{R}_2(\cdot)$ is continuous (Lemma B.2) and $\mathcal{R}_2(0,\cdot;\cdot) \equiv 0$ (Lemma B.1),

$$\lim_{\substack{m_1 \to 0^+ \\ m_2 = 1/m_1}} a_{2,2}^{\mathrm{int}} = 0\,. \tag{C.68}$$

Since $a_{1,2}^{\mathrm{int}}$ increases as $m_1 \to 0^+$ and $m_2 = 1/m_1$ (from Equation (C.64)), either $\lim_{\substack{m_1 \to 0^+ \\ m_2 = 1/m_1}} a_{1,2}^{\mathrm{int}} = \infty$, or there is some $\alpha \in \mathbb{R}$ such that $\lim_{\substack{m_1 \to 0^+ \\ m_2 = 1/m_1}} a_{1,2}^{\mathrm{int}} = \alpha$. Suppose, in order to derive a contradiction, that $\lim_{\substack{m_1 \to 0^+ \\ m_2 = 1/m_1}} a_{1,2}^{\mathrm{int}} = \alpha$. Recall (from Equation (C.62b)) that $a_{1,2}^{\mathrm{int}}$ is the unique solution of

$$-B_1'(\bar{e}_1 - m_1 a_2) + (1+m_2)D_1'\big(\bar{e} - (1+m_1)a_2\big) = 0\,,$$

which, with $m_1 m_2 = 1$, simplifies to

$$-m_1 B_1'(\bar{e}_1 - m_1 a_2) + (1+m_1) D_1'\big(\bar{e} - (1+m_1)a_2\big) = 0\,.$$

Taking $m_1 \to 0^+$ and using the continuity of B_1' and D_1', α must satisfy

$$D_1'\big(\bar{e} - \alpha\big) = 0\,,$$

which contradicts our assumption that $D_1' > 0$. Thus, $\lim_{\substack{m_1 \to 0^+ \\ m_2 = 1/m_1}} a_{1,2}^{\text{int}} = \infty$. This, combined with Equations (C.61) and (C.68) implies that

$$\lim_{m_1 \to 0^+} \Delta\mathcal{R}(m_1) = -\infty\,. \tag{C.69}$$

To see that $\Delta\mathcal{R}(m_1) > 0$ for large enough m_1, interchange countries 1 and 2. An argument analogous to the one justifying Equation (C.61) shows that the vertical distance between the non-zero segments of the two best-response functions in the a_2–a_1 plane, does not depend on a_2, and is equal to $a_{1,1}^{\text{int}} - a_{2,1}^{\text{int}}$. Arguing as we did to justify Equation (C.69),

$$\lim_{\substack{m_2 \to 0^+ \\ m_1 = 1/m_2}} a_{1,1}^{\text{int}} - a_{2,1}^{\text{int}} = -\infty\,.$$

Now, the sign of $\Delta\mathcal{R}(m_1) = a_{2,2}^{\text{int}} - a_{1,2}^{\text{int}}$ is opposite to the sign of $a_{1,1}^{\text{int}} - a_{2,1}^{\text{int}}$, and as m_2 *decreases* to 0, $m_1 = 1/m_2$ *increases* to ∞, so for large enough m_1, $\Delta\mathcal{R}(m_1)$ is positive, as claimed.

It now follows that $\Delta\mathcal{R}(m_1)$ must vanish for some $m_1 \in (0,\infty)$.

C.6.4 Characterization of the continuum of Nash equilibria (proof of Lemma B.14)

From Lemmas B.12 and B.13, there exists a unique pair of matching factors, $(m_1^{\text{c}}, m_2^{\text{c}}) \in \mathcal{M}_{\text{r}}$ such that country 2's best-response function is *overlaps* with country 1's in the interior of the quadrant of positive unconditional abatements, forming a continuum of Nash equilibria. Recall that $a_{1,1}^{\text{int}}$ and $a_{2,1}^{\text{int}}$ satisfy

$$\begin{aligned} B_1'\left(\bar{e}_1 - a_{1,1}^{\text{int}}\right) &= (1+m_2^{\text{c}}) D_1'\big(\bar{e} - (1+m_2^{\text{c}})a_{1,1}^{\text{int}}\big)\,, \\ B_2'\left(\bar{e}_2 - m_2^{\text{c}} a_{2,1}^{\text{int}}\right) &= (1+m_1^{\text{c}}) D_2'\big(\bar{e} - (1+m_2^{\text{c}})a_{2,1}^{\text{int}}\big)\,, \end{aligned}$$

(see Lemma B.1 and Remark B.3). Letting $m = m_2^{\text{c}} = 1/m_1^{\text{c}}$ and $a = a_{1,1}^{\text{int}} = a_{2,1}^{\text{int}}$, we have

$$B_1'\left(\bar{e}_1 - a\right) = (1+m) D_1'\big(\bar{e} - (1+m)a\big)\,, \tag{C.70a}$$

$$m B_2'\left(\bar{e}_2 - ma\right) = (1+m) D_2'\big(\bar{e} - (1+m)a\big)\,. \tag{C.70b}$$

Conversely, if there exist $m > 0$ and $a > 0$ satisfying Equation (C.70) then setting $m_2 = m$, Remark B.3 implies that $a_{1,1}^{\text{int}} = a$, and similarly, setting $m_1 = 1/m_2 = 1/m$, a also solves Equation (B.37), so Lemma B.1 implies that $a_{2,1}^{\text{int}} = a$. Recalling that $\Delta\mathcal{R}(m_1, 1/m_1; a_1)$ is constant for $0 \le a_1 \le a_{1,1}^{\text{int}}$, it follows that $\Delta\mathcal{R}(m_1, 1/m_1; a_1) = 0$ for all $0 \le a_1 \le a_{1,1}^{\text{int}}$, so $(m_1^{\text{c}}, m_2^{\text{c}}) = (1/m, m) \in \mathcal{M}_{\text{r}}$ yields a continuum of Nash equilibria in stage II of the MCG. Consequently, stage II of the MCG has a continuum of Nash equilibria iff $(m_1^{\text{c}}, m_2^{\text{c}}) = (1/m, m)$ are played in stage II, where m and a are the unique positive solutions of Equation (C.70).

To find the countries' abatements when there is a continuum of Nash equilibria, let $(m_1, m_2) = (m_1^{\text{c}}, m_2^{\text{c}}) = (1/m, m)$, where m and a are the unique positive solutions of Equation (C.70). Then, the best-responses overlap for positive unconditional abatements, and from the continuity of the best-response functions (Lemma B.2), there are two edge equilibria, $(0, a_{2,2}^{\text{int}})$ and $(a_{1,1}^{\text{int}}, 0)$ in addition to the interior equilibria. Thus, the set of Nash equilibria is

$$\left\{ \big(a_1, \mathcal{R}_2(m_1, m_2; a_1)\big) \;\middle|\; a_1 \in \left[0, a_{1,1}^{\text{int}}\right] \right\}\,,$$

so the equilibrium unconditional abatements are indeterminate. However, using Equations (C.50) and (C.58), country 2's abatement and the total abatement satisfy

$$\begin{aligned}\frac{d}{da_1}\left(A_2|_{a_2=\mathcal{R}_2(m_1,m_2;a_1)}\right) &= -m_2+m_2=0\,,\\ \frac{d}{da_1}\left(A|_{a_2=\mathcal{R}_2(m_1,m_2;a_1)}\right) &= (1+m_1)(-m_2)+(1+m_2)=0\,.\end{aligned}$$

Hence, the two countries' abatements, A_i and the total abatement A are constant over the set of all Nash equilibria; because the best-response functions $\mathcal{R}_i(m_1,m_2;a_j)$ ($(i,j)=(1,2)$ or $(2,1)$) are continuous, the countries' abatements A_i ($i=1,2$) are given by $A_1=a_{1,1}^{\text{int}}$ and $A_2=a_{2,2}^{\text{int}}$ at *any* of the existing Nash equilibria.

Since the best-response curves overlap in the interior of the quadrant of positive unconditional abatements, and are continuous, $a_{1,1}^{\text{int}}=a_{2,1}^{\text{int}}$, so Equation (C.59) implies that $-m_2a_{1,1}^{\text{int}}+a_{2,2}^{\text{int}}=0$, and hence

$$\begin{aligned}A_1&=a_{1,1}^{\text{int}}=a\,,\\ A_2&=a_{2,2}^{\text{int}}=m_2a_{1,1}^{\text{int}}=ma\,,\\ A&=(1+m)a\,.\end{aligned}$$

Alternatively,

$$A=(1+m)a=(1+m_2)\frac{A_2}{m_2}=(1+m_1)a_{2,2}^{\text{int}}\,.$$

C.7 Map of the matching plane

C.7.1 The stage-II delimiter curves are well-defined

Lemma B.1 and Remark B.3, imply that $a_{i,i}^{\text{int}}=a_{j,i}^{\text{int}}=a\in\mathbb{R}_{\geq 0}$ iff

$$-B_j'(\bar{e}_j-m_ja)+(1+m_i)D_j'\big(\bar{e}-(1+m_j)a\big)=0\,, \tag{C.71a}$$
$$-B_i'(\bar{e}_i-a)+(1+m_j)D_i'\big(\bar{e}-(1+m_j)a\big)=0\,. \tag{C.71b}$$

The unique solution of Equation (C.71b) is $a=a_{i,i}^{\text{int}}\geq 0$ (Remark B.3), which depends only on m_j (but not m_i). We thus abuse notation slightly by writing $a_{i,i}^{\text{int}}(m_j)$. Plugging this into Equation (C.71a), we must show that for a given m_j there is a unique $m_i\geq 0$ which solves

$$-B_j'\big(\bar{e}_j-m_ja_{i,i}^{\text{int}}(m_j)\big)+(1+m_i)D_j'\left(\bar{e}-(1+m_j)a_{i,i}^{\text{int}}(m_j)\right)=0\,. \tag{C.72}$$

The unique solution is

$$m_i=\phi_i(m_j)=-\frac{-B_j'\big(\bar{e}_j-m_ja_{i,i}^{\text{int}}(m_j)\big)+D_j'\left(\bar{e}-(1+m_j)a_{i,i}^{\text{int}}(m_j)\right)}{D_j'\left(\bar{e}-(1+m_j)a_{i,i}^{\text{int}}(m_j)\right)}\,. \tag{C.73}$$

To see that this solution is non-negative, observe that using Equation (C.72),

$$\phi_i(m_j)\geq\phi_i(m_j)-m_i=-\frac{-B_j'\big(\bar{e}_j-m_ja_{i,i}^{\text{int}}(m_j)\big)+(1+m_i)D_j'\left(\bar{e}-(1+m_j)a_{i,i}^{\text{int}}(m_j)\right)}{D_j'\left(\bar{e}-(1+m_j)a_{i,i}^{\text{int}}(m_j)\right)}=0\,.$$

Thus, for a given $m_j\geq 0$, there is a unique $m_i=\phi_i(m_j)\geq 0$ such that Equation (B.43) holds.

C.7.2 The stage-II delimiter curves are differentiable and increasing

The mapping $m_j\mapsto m_i=\phi_i(m_j)$ is continuous (because $a_{i,i}^{\text{int}}(m_j)$ is continuous; see Lemma B.2). Moreover, we will next show that it is differentiable, and calculate its derivative. The Jacobian matrix of the left hand side of Equation (C.71), with respect to m_i and a, is

$$\hat{J}=\begin{pmatrix}D_j'\big(\bar{e}-(1+m_j)a\big) & m_jB_j''(\bar{e}_j-m_ja)-(1+m_i)(1+m_j)D_j''\big(\bar{e}-(1+m_j)a\big)\\ 0 & B_i''(\bar{e}_i-a)-(1+m_j)^2D_i''\big(\bar{e}-(1+m_j)a\big)\end{pmatrix}\,,$$

which is invertible because its determinant is nonzero:

$$\det \hat{J} = D_j'\big(\overline{e} - (1+m_j)a\big)\Big[B_i''\left(\overline{e}_i - a\right) - (1+m_j)^2 D_i''\big(\overline{e} - (1+m_j)a\big)\Big] < 0\,, \tag{C.74}$$

(Equation (A.3)). Hence, from the implicit function theorem [6],

$$\frac{\mathrm{d}}{\mathrm{d}m_j}\begin{pmatrix}\phi_i(m_j)\\ a_{i,i}^{\mathrm{int}}(m_j)\end{pmatrix} = -\hat{J}^{-1}\begin{pmatrix} a\Big[B_j''(\overline{e}_j - m_j a) - (1+m_i)D_j''\big(\overline{e} - (1+m_j)a\big)\Big]\\ D_i'\big(\overline{e} - (1+m_j)a\big) - a(1+m_j)D_i''\big(\overline{e} - (1+m_j)a\big)\end{pmatrix}\,,$$

where we implicitly set $a = a_{i,i}^{\mathrm{int}}(m_j)$ and $m_i = \phi_i(m_j)$. Since

$$\hat{J}^{-1} = \frac{1}{\det \hat{J}}\begin{pmatrix} B_i''\left(\overline{e}_i - a\right) - (1+m_j)^2 D_i''\big(\overline{e} - (1+m_j)a\big) & -m_j B_j''(\overline{e}_j - m_j a) + (1+m_i)(1+m_j)D_j''\big(\overline{e} - (1+m_j)a\big)\\ 0 & D_j'\big(\overline{e} - (1+m_j)a\big)\end{pmatrix}\,,$$

we get

$$\begin{aligned}
\det \hat{J}\phi_i'(m_j) = {} & \Big[B_i''\left(\overline{e}_i - a\right) - (1+m_j)^2 D_i''\big(\overline{e} - (1+m_j)a\big)\Big]\\
& \times a\Big[-B_j''(\overline{e}_j - m_j a) + (1+m_i)D_j''\big(\overline{e} - (1+m_j)a\big)\Big]\\
& + \Big[-m_j B_j''(\overline{e}_j - m_j a) + (1+m_i)(1+m_j)D_j''\big(\overline{e} - (1+m_j)a\big)\Big]\\
& \times \Big[-D_i'\big(\overline{e} - (1+m_j)a\big) + a(1+m_j)D_i''\big(\overline{e} - (1+m_j)a\big)\Big]\\
= {} & -aB_i''\left(\overline{e}_i - a\right)B_j''(\overline{e}_j - m_j a)\\
& + (1+m_i)aB_i''\left(\overline{e}_i - a\right)D_j''\big(\overline{e} - (1+m_j)a\big)\\
& + (1+m_j)^2 aB_j''(\overline{e}_j - m_j a)D_i''\big(\overline{e} - (1+m_j)a\big)\\
& - (1+m_i)(1+m_j)^2 aD_i''\big(\overline{e} - (1+m_j)a\big)D_j''\big(\overline{e} - (1+m_j)a\big)\\
& + m_j B_j''(\overline{e}_j - m_j a)D_i'\big(\overline{e} - (1+m_j)a\big)\\
& - m_j(1+m_j)aB_j''(\overline{e}_j - m_j a)D_i''\big(\overline{e} - (1+m_j)a\big)\\
& - (1+m_i)(1+m_j)D_i'\big(\overline{e} - (1+m_j)a\big)D_j''\big(\overline{e} - (1+m_j)a\big)\\
& + (1+m_i)(1+m_j)^2 aD_i''\big(\overline{e} - (1+m_j)a\big)D_j''\big(\overline{e} - (1+m_j)a\big)\\
= {} & -aB_i''\left(\overline{e}_i - a\right)B_j''(\overline{e}_j - m_j a)\\
& + (1+m_i)aB_i''\left(\overline{e}_i - a\right)D_j''\big(\overline{e} - (1+m_j)a\big)\\
& + (1+m_j)aB_j''(\overline{e}_j - m_j a)D_i''\big(\overline{e} - (1+m_j)a\big)\\
& + m_j B_j''(\overline{e}_j - m_j a)D_i'\big(\overline{e} - (1+m_j)a\big)\\
& - (1+m_i)(1+m_j)D_i'\big(\overline{e} - (1+m_j)a\big)D_j''\big(\overline{e} - (1+m_j)a\big) < 0\,,
\end{aligned}$$

since all of the summands on the right hand side are non-positive, and the last two are strictly negative. It follows from Equation (C.74) that $\det \hat{J} < 0$, so

$$\phi_i'(m_j) > 0\,,$$

and hence ϕ_i is an increasing (and thus injective) function of m_j.

C.7.3 The stage-II delimiter curves are onto $\mathbb{R}_{\geq 0}$

Observe that since $a_{i,i}^{\mathrm{int}}(0) = 0$ (Remark B.3) and $B_j'(\overline{e}_j) = D_j'(\overline{e})$ (Lemma A.5), so from Equation (C.73) we have

$$\phi_i(0) = -\frac{-B_j'\left(\overline{e}_j\right) + D_j'\left(\overline{e}\right)}{D_j'\left(\overline{e}\right)} = 0\,. \tag{C.75}$$

We claim that

$$\lim_{m_j\to\infty} \phi_i(m_j) = \infty\,, \tag{C.76}$$

which implies that ϕ_i is onto $\mathbb{R}_{\geq 0}$ (since it is continuous).

To justify Equation (C.76), suppose, in order to derive a contradiction, that

$$\lim_{m_j\to\infty} \phi_i(m_j) \neq \infty\,.$$

Because ϕ_i is increasing (Appendix C.7.2), this implies that

$$\lim_{m_j\to\infty} \phi_i(m_j) = \mu_i \in (0,\infty)\,.$$

Let $\{m_{j,k}\}_{k=1}^{\infty} \subset \mathbb{R}_{\geq 0}$ be an increasing, unbounded sequence such that

$$\lim_{k\to\infty} a_{i,i}^{\text{int}}(m_{j,k}) = \alpha = \liminf_{m_j\to\infty} a_{i,i}^{\text{int}}(m_j) \in [0,\infty]\,,$$

There are two possibilities:

$\alpha > 0$: From Equation (C.71a), we have

$$B_j'\left(\bar{e}_j - m_{j,k}a_{i,i}^{\text{int}}(m_{j,k})\right) = \big(1+\phi_i\left(m_{j,k}\right)\big)D_j'\left(\bar{e} - (1+m_{j,k})a_{i,i}^{\text{int}}(m_{j,k})\right)\,,$$

which implies that

$$\lim_{e\to-\infty} B_j'(e) = (1+\mu_i)\lim_{e\to-\infty} D_j'(e)\,,$$

contradicting Equations (A.10) and (B.34).

$\alpha = 0$: Taking $m_j \to \infty$ in Equation (C.71b), we have

$$B_i'\left(\bar{e}_i\right) = \lim_{k\to\infty} B_i'\left(\bar{e}_i - a_{i,i}^{\text{int}}(m_{j,k})\right) = \lim_{k\to\infty}(1+m_{j,k})D_i'\big(\bar{e} - (1+m_{j,k})a_{i,i}^{\text{int}}(m_{j,k})\big)\,.$$

It follows that

$$\lim_{k\to\infty} D_i'\big(\bar{e} - (1+m_{j,k})a_{i,i}^{\text{int}}(m_{j,k})\big) = 0\,,$$

and hence

$$\lim_{k\to\infty} m_{j,k}a_{i,i}^{\text{int}}(m_{j,k}) = \lim_{m_{j,k}\to\infty}(1+m_{j,k})a_{i,i}^{\text{int}}(m_{j,k}) = \infty\,.$$

Equation (C.71a) then gives

$$\lim_{e\to-\infty} B_j'(e) = \lim_{k\to\infty} B_j'\big(\bar{e}_j - m_{j,k}a_{i,i}^{\text{int}}(m_{j,k})\big) = \lim_{k\to\infty}\big(1+\phi_i\left(m_{j,k}\right)\big)D_j'\big(\bar{e} - (1+m_{j,k})a_{i,i}^{\text{int}}(m_{j,k})\big) = 0\,,$$

in contradiction to the fact that $\lim_{e\to-\infty} B_j'(e) \in (0,\infty]$ (Equation (A.10)).

It follows that Equation (C.76) must hold.

C.7.4 Intersections of the stage-II delimiter curves

We wish to characterize the pairs of matching factors $(m_1, m_2) \in \mathbb{R}_{\geq 0}^2$ for which $m_1 = \phi_1(m_2)$ and $m_2 = \phi_2(m_1)$.

- If $m_1 = 0$, then from Equation (C.75), $m_2 = \phi_2(m_1) = 0$, and $m_1 = \phi_1(0) = 0$, so ϕ_1 and ϕ_2 intersect at the origin, $m_1 = m_2 = 0$, and do not intersect at any other (m_1, m_2) on the axes (i.e., such that $m_1m_2 = 0$).
- If $m_1m_1 \neq 1$, Proposition B.9 implies that $a_{1,1}^{\text{int}} = a_{2,1}^{\text{int}}$ and $a_{2,2}^{\text{int}} = a_{1,2}^{\text{int}}$ cannot both simultaneously hold, and hence ϕ_i $(i = 1,2)$ do not intersect at (m_1, m_2).
- If $m_1m_2 = 1$, Lemma B.14 implies that $a_{1,1}^{\text{int}} = a_{2,1}^{\text{int}}$ and $a_{2,2}^{\text{int}} = a_{1,2}^{\text{int}}$ iff $(m_1, m_2) = (m_1^{\text{c}}, m_2^{\text{c}})$.

Thus, the curves ϕ_i $(i = 1,2)$ intersect only at $(m_1, m_2) = (0,0)$ and $(m_1^{\text{c}}, m_2^{\text{c}})$.

C.7.5 Edge equilibria and the stage-II delimiter curves

Since $a_{i,i}^{\text{int}} - a_{j,i}^{\text{int}}$ is continuous (because the best-response functions are continuous; see Lemma B.2) and zero iff $\phi_i(m_1) = m_2$, its sign must be constant on each of the sets

$$\{(m_1, m_2) \mid \phi_i(m_1) > m_2\} \quad \text{and} \quad \{(m_1, m_2) \mid \phi_i(m_1) < m_2\} .$$

From Lemma B.1 and Remark B.3, if $m_i > 0$ and $m_j = 0$ then $a_{j,i}^{\text{int}} > 0$ and $a_{i,i}^{\text{int}} = 0$, so $a_{i,i}^{\text{int}} - a_{j,i}^{\text{int}} < 0$ for all (m_1, m_2) such that $m_i > \phi_i(m_j)$. Similarly, if $m_i = 0$ and $m_j > 0$ then $a_{j,i}^{\text{int}} = 0$ and $a_{i,i}^{\text{int}} > 0$, so $a_{i,i}^{\text{int}} - a_{j,i}^{\text{int}} > 0$ for all (m_1, m_2) such that $m_i < \phi_i(m_j)$.

Remark B.5 then implies that for any $(m_i, m_j) \neq (0,0)$, $(a_i^*, a_j^*) = (a_{i,i}^{\text{int}}, 0)$ is a stage-II Nash equilibrium iff $m_i \leq \phi_i(m_j)$.

C.7.6 The distance between the stage-II delimiter curves

Let $\Delta\phi$ be the distance between the curves ϕ_i $(i = 1, 2)$ along the m_2 axis,

$$\Delta\phi(m_1) = \phi_2(m_1) - \phi_1^{-1}(m_1) .$$

Since ϕ_i intersect only at $(m_1, m_2) = (0, 0)$ and $(m_1^{\text{c}}, m_2^{\text{c}})$ (Appendix C.7.4), $\Delta\phi(m_1)$ is 0 iff $m_1 = 0$ or m_1^{c}, and the sign of $\Delta\phi(m_1)$ must be constant on each of the intervals $(0, m_1^{\text{c}})$ and (m_1^{c}, ∞). We will show that $\Delta\phi(m_1) < 0$ if $(0, m_1^{\text{c}})$ and $\Delta\phi(m_1) > 0$ if $m_1 \in (m_1^{\text{c}}, \infty)$.

To see that $\Delta\phi(m_1) < 0$ on $(0, m_1^{\text{c}})$, suppose, in order to derive a contradiction, that $\Delta\phi(m_1) > 0$ for some $m_1 \in (0, m_1^{\text{c}})$. Let $m_2 \in \big(\phi_1^{-1}(m_1), \phi_2(m_1)\big)$. Since $m_2 < \phi_2(m_1)$, by Appendix C.7.5, $a_{2,2}^{\text{int}} - a_{1,2}^{\text{int}} > 0$ and $(a_1^*, a_2^*) = (0, a_{2,2}^{\text{int}})$ is a stage-II Nash equilibrium. Similarly, $m_2 > \phi_1^{-1}(m_1)$ so (since ϕ_1 is increasing; see Appendix C.7.2), $\phi_1(m_2) > m_1$, and hence $a_{1,1}^{\text{int}} - a_{2,1}^{\text{int}} > 0$ and $(a_1^*, a_2^*) = (a_{1,1}^{\text{int}}, 0)$ is a stage-II Nash equilibrium. However, this contradicts Proposition B.9, so $\Delta\phi(m_1) < 0$ for all $m_1 \in (0, m_1^{\text{c}})$.

The proof that $\Delta\phi(m_1) > 0$ for all $m_1 \in (m_1^{\text{c}}, \infty)$ is analogous to the above argument showing that $\Delta\phi(m_1) < 0$ for all $m_1 \in (0, m_1^{\text{c}})$.

It follows that $\phi_1^{-1}(m_1) > \phi_2(m_1)$ if $0 < m_1 < m_1^{\text{c}}$ and $\phi_2(m_1) > \phi_1^{-1}(m_1)$ if $m_1 > m_1^{\text{c}}$, or geometrically, ϕ_i is closer (resp. farther) than ϕ_j to (from) the a_j-axis for $0 < m_1 m_2 < 1$ ($m_1 m_2 > 1$).

C.8 Multiple possible stage I payoffs under stage-II equilibrium play

In this appendix, we show that for any benefit and damage functions B_i, D_i $(i = 1, 2)$, there are matching factors for which the payoff functions of the stage I game, assuming equilibrium play in stage II, are not uniquely defined. This follows immediately from the following proposition (proved below):

Proposition C.3. *There exist infinitely many matching factors (m_1, m_2) satisfying $m_1 m_2 > 1$ and for which two edge equilibria coexist with an interior equilibrium in stage II. Moreover, when this occurs, each country's payoff is* lowest *at the stage-II equilibrium in which it does not abate unconditionally, and* highest *at the equilibrium in which the other country does not abate unconditionally.*

The relationship between the countries' payoffs at the edge equilibria also holds for (m_1, m_2) that yield only two edge equilibria: country i's payoff is highest at the equilibrium $(a_{i,i}^{\text{int}}, 0)$.

Proof. First, recall from Appendix C.7.6 that $\Delta\phi(m_1) > 0$ for all $m_1 \in (m_1^{\text{c}}, \infty)$. Thus, for any $m_1 > m_1^{\text{c}}$, $\big(\phi_1^{-1}(m_1), \phi_2(m_1)\big) \neq \phi$. From Appendix C.7.5, for any pair of matching factors (m_1, m_2) such that $m_2 \in \big(\phi_1^{-1}(m_1), \phi_2(m_1)\big)$ $a_{1,1}^{\text{int}} > a_{2,1}^{\text{int}}$ and $a_{2,2}^{\text{int}} > a_{1,2}^{\text{int}}$, and Propositions B.9 and B.10 imply that there are three stage-II equilibria: two edge equilibria and an interior equilibrium. Note also that for such a pair of matching factors, since ϕ_1 is increasing and $m_1 > m_1^{\text{c}}$,

$$m_2 > \phi_1^{-1}(m_1) > \phi_1^{-1}(m_1^{\text{c}}) = m_2^{\text{c}} ,$$

so $m_1 m_2 > 1$.

Now consider (m_1, m_2) such that $m_1 m_2 > 1$ and for which there are two edge equilibria and an interior equilibrium (a_1^*, a_2^*) in stage II. To order country 1's payoffs at the three equilibria, note first that if $0 < a_2 < a_{1,2}^{\text{int}}$,

$$\begin{aligned}\frac{\mathrm{d}}{\mathrm{d}a_2}\Pi_1\left(m_1, m_2, \mathcal{R}_1(a_2), a_2\right) = & \left.\frac{\partial}{\partial a_2}\Pi_1\left(m_1, m_2, a_1, a_2\right)\right|_{a_1=\mathcal{R}_1(a_2)} \\ & + \left.\frac{\partial}{\partial a_1}\Pi_1\left(m_1, m_2, a_1, a_2\right)\right|_{a_1=\mathcal{R}_1(a_2)} \frac{\partial}{\partial a_2}\mathcal{R}_1\left(a_2\right) .\end{aligned}$$

Because $a_2 \in (0, a_{1,2}^{\text{int}})$, $a_1 = \mathcal{R}_1(a_2)$ solves Equation (B.36), that is,

$$\frac{\partial}{\partial a_1}\Pi_1\left(m_1, m_2, a_1, a_2\right) = -B_1'\left(\bar{e}_1 - A_1\right) + (1 + m_2)D_1'\left(\bar{e} - A\right) = 0\,, \tag{C.77}$$

and hence

$$\begin{aligned}\frac{\mathrm{d}}{\mathrm{d}a_2}\Pi_1\left(m_1, m_2, \mathcal{R}_1(a_2), a_2\right) = & \left.\frac{\partial}{\partial a_2}\Pi_1\left(m_1, m_2, a_1, a_2\right)\right|_{a_1=\mathcal{R}_1(a_2)} \\ = & -m_1 B_1'\left(\bar{e}_1 - A_1\right) + (1 + m_1)D_1'\left(\bar{e} - A\right) ,\end{aligned}$$

where

$$\begin{aligned}A_1 &= \mathcal{R}_1(a_2) + m_1 a_2\,, \\ A &= (1 + m_1)a_2 + (1 + m_2)\mathcal{R}_1(a_2)\,.\end{aligned}$$

But from Equation (C.77), $B_1'\left(\bar{e}_1 - A_1\right) = (1 + m_2)D_1'\left(\bar{e} - A\right)$, so since $m_1 m_2 > 1$, we have

$$\begin{aligned}\frac{\mathrm{d}}{\mathrm{d}a_2}\Pi_1\left(m_1, m_2, \mathcal{R}_1(a_2), a_2\right) &= -m_1(1 + m_2)D_1'\left(\bar{e} - A\right) + (1 + m_1)D_1'\left(\bar{e} - A\right) \\ &= (1 - m_1 m_2)D_1'\left(\bar{e} - A\right) < 0\,, \end{aligned} \tag{C.78}$$

implying that

$$\Pi_1(m_1, m_2, 0, a_{1,2}^{\text{int}}) < \Pi_1(m_1, m_2, a_1^*, a_2^*) < \Pi_1(m_1, m_2, a_{1,1}^{\text{int}}, 0)\,. \tag{C.79}$$

Note also that for all $a_2 > a_{1,2}^{\text{int}}$

$$\left.\frac{\partial}{\partial a_1}\Pi_1(m_1, m_2, a_1, a_2)\right|_{a_1=0} = -B_1'\left(\bar{e}_1 - m_1 a_2\right) + (1 + m_2)D_1'\big(\bar{e} - (1 + m_1)a_2\big) < 0\,,$$

(Equations (B.37) and (C.48)), and because $m_1 m_2 > 1$

$$\begin{aligned}\frac{\partial}{\partial a_2}\Pi_1(m_1, m_2, 0, a_2) &= -m_1 B_1'\left(\bar{e}_1 - m_1 a_2\right) + (1 + m_1)D_1'\big(\bar{e} - (1 + m_1)a_2\big) \\ &< -m_1(1 + m_2)D_1'\big(\bar{e} - (1 + m_1)a_2\big) + (1 + m_1)D_1'\big(\bar{e} - (1 + m_1)a_2\big) \\ &= (1 - m_1 m_2)D_1'\big(\bar{e} - (1 + m_1)a_2\big) < 0\,. \end{aligned} \tag{C.80}$$

In particular, since $a_{2,2}^{\text{int}} > a_{1,2}^{\text{int}}$,

$$\Pi_1(m_1, m_2, 0, a_{2,2}^{\text{int}}) < \Pi_1(m_1, m_2, 0, a_{1,2}^{\text{int}})\,. \tag{C.81}$$

Equation (C.81) is intuitive: when $a_2 = a_{1,2}^{\text{int}}$, country 1 does best by abating nothing; if country 2 then increases its unconditional abatement (a_2), then country 1 does worse because it cannot decrease its unconditional abatement (it is already 0), and its abatement increases $(A_1 = m_2 a_2)$.

Combining Equation (C.81) with Equation (C.79) gives

$$\Pi_1(m_1, m_2, 0, a_{2,2}^{\text{int}}) < \Pi_1(m_1, m_2, a_1^*, a_2^*) < \Pi_1(m_1, m_2, a_{1,1}^{\text{int}}, 0)\,.$$

This result is surprising: country 1 prefers the equilibrium in which country 2 contributes no unconditional abatement to the one in which country 1 contributes no unconditional abatement. This is because country 1's payoff Π_1 decreases along its best-response function $a_1 = \mathcal{R}_1(m_1, m_2; a_2)$ (Equation (C.79)). This in turn is a result of country 1's abatement (A_1) increasing, but the total abatement (A) decreasing along $\mathcal{R}_1$, which can be seen using Lemma B.2:

$$\begin{aligned}\frac{\partial}{\partial a_2}\left[A_1\big|_{a_1=\mathcal{R}_1(a_2)}\right] &= \frac{\partial}{\partial a_2}\mathcal{R}_1(a_2) + m_1 = -\frac{m_1 B_1''(\bar{e}_1 - A_i) - (1+m_1)(1+m_2) D_1''(\bar{e} - A)}{B_1''(\bar{e}_1 - A_1) - (1+m_2)^2 D_1''(\bar{e} - A)} + m_1 \\ &= \frac{-m_1 B_1''(\bar{e}_1 - A_i) + (1+m_1)(1+m_2) D_1''(\bar{e} - A) + m_1 B_1''(\bar{e}_1 - A_1) - m_1 (1+m_2)^2 D_1''(\bar{e} - A)}{B_1''(\bar{e}_1 - A_1) - (1+m_2)^2 D_1''(\bar{e} - A)} \\ &= \frac{(1 - m_1 m_2)(1+m_2) D_1''(\bar{e} - A)}{B_1''(\bar{e}_1 - A_1) - (1+m_2)^2 D_1''(\bar{e} - A)} > 0\,,\end{aligned}$$

$$\begin{aligned}\frac{\partial}{\partial a_2}\left[A\big|_{a_1=\mathcal{R}_1(a_2)}\right] &= (1+m_2)\frac{\partial}{\partial a_2}\mathcal{R}_1(a_2) + 1 + m_1 \\ &= -(1+m_2)\frac{m_1 B_1''(\bar{e}_1 - A_i) - (1+m_1)(1+m_2) D_1''(\bar{e} - A)}{B_1''(\bar{e}_1 - A_1) - (1+m_2)^2 D_1''(\bar{e} - A)} + 1 + m_1 \\ &= \frac{-(1+m_2)\left[m_1 B_1''(\bar{e}_1 - A_i) - (1+m_1)(1+m_2) D_1''(\bar{e} - A)\right]}{B_1''(\bar{e}_1 - A_1) - (1+m_2)^2 D_1''(\bar{e} - A)} \\ &\quad + \frac{(1+m_1)\left[B_1''(\bar{e}_1 - A_1) - (1+m_2)^2 D_1''(\bar{e} - A)\right]}{B_1''(\bar{e}_1 - A_1) - (1+m_2)^2 D_1''(\bar{e} - A)} \\ &= \frac{(1 - m_1 m_2) B_1''(\bar{e}_1 - A_i)}{B_1''(\bar{e}_1 - A_1) - (1+m_2)^2 D_1''(\bar{e} - A)} < 0\,.\end{aligned}$$

Now note that if

$$\begin{cases} a_{1,1}^{\text{int}} &= a_{2,1}^{\text{int}}\,, \\ a_{2,2}^{\text{int}} &> a_{1,2}^{\text{int}}\,, \end{cases}$$

Equations (C.78) and (C.80) still imply that

$$\Pi_1(m_1, m_2, 0, a_{2,2}^{\text{int}}) < \Pi_1(m_1, m_2, a_{1,1}^{\text{int}}, 0)\,;$$

this is also true if

$$\begin{cases} a_{1,1}^{\text{int}} &> a_{2,1}^{\text{int}}\,, \\ a_{2,2}^{\text{int}} &= a_{1,2}^{\text{int}}\,, \end{cases}$$

(but only Equation (C.78) is required to see this).

A symmetric argument shows that analogous inequalities hold for country 2's payoff, Π_2. □

C.9 Baseline is not an equilibrium of the matching climate game (proof of Proposition B.16)

If $m_2 = 0$, then for any $m_1 > 0$ the unique stage-II Nash equilibrium is $(0, a_{2,2}^{\text{int}})$ (Proposition B.8). We can thus use Lemma C.6 (or alternatively Lemma C.5) and the continuity of the best-response functions (Lemma B.2) to see that

$$\lim_{m_1 \to 0^+} \frac{\mathrm{d}}{\mathrm{d}m_1}\Pi_1\big(m_1, 0, 0, a_{2,2}^{\text{int}}(m_1, 0)\big) = \frac{-D_1'(\bar{e})\, D_2'(\bar{e})}{B_2''(\bar{e}_2) - D_2''(\bar{e})} > 0\,.$$

It follows that if both countries play $m_1 = m_2 = 0$, and equilibrium play is assumed in stage II, country 1 can increase its payoff by increasing its matching factor unilaterally, so $m_1 = m_2 = 0$ cannot be on the equilibrium path of an SPE. A similar argument shows that country 2 can also increase its payoff by increasing its matching factor.

C.10 A cooperative equilibrium exists and dominates baseline (proof of Proposition B.17)

C.10.1 Proof of existence of cooperative equilibrium

Here, we prove that for any stage-II equilibrium choice

$$\big(a_1\left(\mu_1, \mu_2\right), a_2\left(\mu_1, \mu_2\right)\big) \in \mathcal{A}\,,$$

the strategy profile

$$\big(m_i^{\mathrm{c}}, a_i\left(\mu_1, \mu_2\right)\big) \quad \text{for } i = 1, 2\,,$$

is an SPE. To do so, we show that country 1 has no incentive to deviate from its strategy; an analogous argument also applies to country 2.

Suppose that country 1 alters its strategy to $\big(m_1, \alpha_i\left(\mu_1, \mu_2\right)\big)$. First, note that (from the principle of backwards induction; see [8]) we can assume that for any pair of matching factors chosen in stage I, Nash equilibria are played in stage II, that is, $\big(\alpha_1\left(\mu_1, \mu_2\right), a_2\left(\mu_1, \mu_2\right)\big) \in \mathcal{A}$. Country 1 then has no incentive to deviate only in stage II, because when the matching factors $(m_1^{\mathrm{c}}, m_2^{\mathrm{c}})$ are played in stage I $\big(a_1\left(m_1^{\mathrm{c}}, m_2^{\mathrm{c}}\right), a_2\left(m_1^{\mathrm{c}}, m_2^{\mathrm{c}}\right)\big)$ is a Nash equilibrium in stage II. Thus, we can further restrict attention to situations in which $m_1 \neq m_1^{\mathrm{c}}$.

Since country 1 deviates unilaterally, the pairs of matching factors possible in stage I are $m_1 \in \mathbb{R}_{\geq 0} \setminus \{m_1^{\mathrm{c}}\}$ and $m_2 = m_2^{\mathrm{c}}$. Lemma C.4 (which follows immediately from Appendix C.7.5) characterizes the stage-II Nash equilibria that can occur when country 1 plays $m_1 \neq m_2^{\mathrm{c}}$ in stage 1.

Lemma C.4 (stage-II Nash equilibria when country 1 deviates from equilibrium matching)**.** *If $m_2 = m_2^{\mathrm{c}}$ then for any $m_1 \neq m_1^{\mathrm{c}}$, there is a unique Nash equilibrium in stage II: if $m_1 < m_1^{\mathrm{c}}$ then the Nash equilibrium is $(a_{1,1}^{\mathrm{int}}, 0)$, and if $m_1 > m_1^{\mathrm{c}}$ then it is $(0, a_{2,2}^{\mathrm{int}})$.*

Thus, since we can assume equilibrium play in stage II, country 1's payoff is

$$\Pi_1(m_1, m_2^{\mathrm{c}}) = \begin{cases} B_1\left(\bar{e}_1 - a_{1,1}^{\mathrm{int}}\right) - D_1\big(\bar{e} - (1 + m_2^{\mathrm{c}})a_{1,1}^{\mathrm{int}}\big) & \text{for } m_1 \in (0, m_1^{\mathrm{c}}]\,, \\ B_1\left(\bar{e}_1 - m_1 a_{2,2}^{\mathrm{int}}\right) - D_1\big(\bar{e} - (1 + m_1^{\mathrm{c}})a_{2,2}^{\mathrm{int}}\big) & \text{for } m_1 \in (m_1^{\mathrm{c}}, \infty)\,. \end{cases}$$

Observe that $\Pi_1(m_1, m_2^{\mathrm{c}})$ is continuous at $m_1 = m_1^{\mathrm{c}}$, because $a_{1,1}^{\mathrm{int}}$ and $a_{2,2}^{\mathrm{int}}$ are continuous in m_1 (Lemma B.2) and because if $(m_1, m_2) = (m_1^{\mathrm{c}}, m_2^{\mathrm{c}})$, then A_1 and A are constant over the set of stage-II Nash equilibria, which contains $(a_{1,1}^{\mathrm{int}}, 0)$ and $(0, a_{2,2}^{\mathrm{int}})$ (Lemma B.14), so in particular,

$$\Pi_1(m_1^{\mathrm{c}}, m_2^{\mathrm{c}}) = B_1\left(\bar{e}_1 - a_{1,1}^{\mathrm{int}}\right) - D_1\left(\bar{e} - (1 + m_2^{\mathrm{c}})a_{1,1}^{\mathrm{int}}\right) = B_1\left(\bar{e}_1 - m_1^{\mathrm{c}} a_{2,2}^{\mathrm{int}})\right) - D_1\left(\bar{e} - (1 + m_1^{\mathrm{c}})a_{2,2}^{\mathrm{int}}\right)\,.$$

Consider first the case in which country 1 plays $m_1 < m_1^{\mathrm{c}}$. From Lemma C.5 in Appendix C.10.3, $a_{1,1}^{\mathrm{int}}$ is constant as a function of m_1, so

$$\begin{aligned} \Pi_1(m_1, m_2^{\mathrm{c}}) &= B_1\left(\bar{e}_1 - a_{1,1}^{\mathrm{int}}(m_1, m_2^{\mathrm{c}})\right) - D_1\left(\bar{e} - (1 + m_2^{\mathrm{c}})a_{1,1}^{\mathrm{int}}(m_1, m_2^{\mathrm{c}})\right) \\ &= B_1\left(\bar{e}_1 - a_{1,1}^{\mathrm{int}}(m_1^{\mathrm{c}}, m_2^{\mathrm{c}})\right) - D_1\left(\bar{e} - (1 + m_2^{\mathrm{c}})a_{1,1}^{\mathrm{int}}(m_1^{\mathrm{c}}, m_2^{\mathrm{c}})\right) = \Pi_1(m_1^{\mathrm{c}}, m_2^{\mathrm{c}})\,. \end{aligned} \tag{C.82}$$

Consequently, country 1 has no incentive to decrease its matching factor.

The case in which country 1 plays $m_1 > m_1^{\mathrm{c}}$ is slightly more involved. However, Lemma C.6 in Appendix C.10.4 shows that $\frac{\partial}{\partial m_1}\Pi_1 < 0$ for all (m_1, m_2) such that $m_1 > m_1^{\mathrm{c}}$ and $m_2 = m_2^{\mathrm{c}}$, and hence $\Pi_1(m_, m_2^{\mathrm{c}})$ decreases for $m_1 \in [m_1^{\mathrm{c}}, \infty)$ and country 1 has no incentive to increase its matching factor.

C.10.2 Both countries' payoffs are higher at the cooperative equilibrium emissions profile than at baseline

From Equation (C.82)

$$\Pi_1(0, m_2^{\mathrm{c}}) = \Pi_1(m_1^{\mathrm{c}}, m_2^{\mathrm{c}})\,.$$

We will show that for all $m_2 \in (0, m_2^{\mathrm{c}})$,

$$\frac{\mathrm{d}}{\mathrm{d}m_2}\Pi_1(0, m_2) > 0\,,$$

which will imply that

$$\Pi_1(0,0) < \Pi_1(0, m_2^{\mathrm{c}}) = \Pi_1(m_1^{\mathrm{c}}, m_2^{\mathrm{c}})\,.$$

Because the baseline emissions profile is the unique stage-II equilibrium when $m_1 = m_2 = 0$ (Proposition B.7), this will complete our proof.

If $m_1 = 0$ and $m_2 \in (0, m_2^{\mathrm{c}})$, the $(a_{1,1}^{\mathrm{int}}, 0)$ is the unique stage-II Nash equilibrium (Proposition B.8), so country 1's payoff is

$$\Pi_1\big(0, m_2\big) = B_1\left(\bar{e}_1 - a_{1,1}^{\mathrm{int}}(0, m_2)\right) - D_1\big(\bar{e} - (1+m_2)a_{1,1}^{\mathrm{int}}(0, m_2)\big)\,.$$

Using Remark B.3, we have

$$B_1'\left(\bar{e}_1 - a_{1,1}^{\mathrm{int}}(0, m_2)\right) = (1+m_2)D_1'\left(\bar{e} - (1+m_2)a_{1,1}^{\mathrm{int}}(0, m_2)\right)\,,$$

so (writing $A = (1+m_2)a_{1,1}^{\mathrm{int}}(0, m_2)$ for convenience)

$$\begin{aligned}
\frac{\mathrm{d}}{\mathrm{d}m_2}\Pi_1\big(0, m_2\big) &= -B_1'\left(\bar{e}_1 - a_{1,1}^{\mathrm{int}}\right)\frac{\partial}{\partial m_2}a_{1,1}^{\mathrm{int}} + D_1'\big(\bar{e} - A\big)\frac{\partial}{\partial m_2}A \\
&= -(1+m_2)D_1'\left(\bar{e} - A\right)\frac{\partial}{\partial m_2}a_{1,1}^{\mathrm{int}} + D_1'\big(\bar{e} - A\big)\frac{\partial}{\partial m_2}A \\
&= D_1'\left(\bar{e} - A\right)\left[-(1+m_2)\frac{\partial}{\partial m_2}a_{1,1}^{\mathrm{int}} + \frac{\partial}{\partial m_2}\left((1+m_2)a_{1,1}^{\mathrm{int}}\right)\right] = D_1'\left(\bar{e} - A\right)a_{1,1}^{\mathrm{int}} > 0\,,
\end{aligned}$$

as claimed.

C.10.3 Derivatives of the intercepts of the best-response functions with the unconditional abatement axes

In this section, we prove the following lemma:

Lemma C.5 (Derivatives of the intercepts of the best-response functions with the unconditional abatement axes)**.** *If $m_1, m_2 > 0$, then*

$$\begin{aligned}
&\frac{\partial}{\partial m_1}a_{1,1}^{\mathrm{int}} = 0\,, \\
&\frac{\partial}{\partial m_1}a_{2,2}^{\mathrm{int}} = -\frac{D_2'\big(\bar{e} - (1+m_1)a_{2,2}^{\mathrm{int}}\big) - (1+m_1)a_{2,2}^{\mathrm{int}}D_2''\big(\bar{e} - (1+m_1)a_{2,2}^{\mathrm{int}}\big)}{B_2''\left(\bar{e}_2 - a_{2,2}^{\mathrm{int}}\right) - (1+m_1)^2D_2''\big(\bar{e} - (1+m_1)a_{2,2}^{\mathrm{int}}\big)}\,, \\
&\frac{\partial}{\partial m_1}a_{1,2}^{\mathrm{int}} = -a_{1,2}^{\mathrm{int}}\frac{B_1''\left(\bar{e}_1 - m_1a_{1,2}^{\mathrm{int}}\right) - (1+m_2)D_1''\big(\bar{e} - (1+m_1)a_{1,2}^{\mathrm{int}}\big)}{m_1B_1''\left(\bar{e}_1 - m_1a_{1,2}^{\mathrm{int}}\right) - (1+m_1)(1+m_2)D_1''\big(\bar{e} - (1+m_1)a_{1,2}^{\mathrm{int}}\big)} < 0\,, \\
&\frac{\partial}{\partial m_1}a_{2,1}^{\mathrm{int}} = -\frac{D_2'\big(\bar{e} - (1+m_2)a_{2,1}^{\mathrm{int}}\big)}{m_2B_2''\left(\bar{e}_2 - m_2a_{2,1}^{\mathrm{int}}\right) - (1+m_1)(1+m_2)D_2''\left(\bar{e} - (1+m_2)a_{2,1}^{\mathrm{int}}\right)} > 0\,;
\end{aligned}$$

analogous formulae hold for the derivatives of $a_{1,1}^{\mathrm{int}}$ $a_{1,2}^{\mathrm{int}}$, $a_{2,1}^{\mathrm{int}}$ and $a_{2,2}^{\mathrm{int}}$ with respect to m_2, mutatis mutandis.

Proof. Since $a_{i,i}^{\mathrm{int}} = \mathcal{R}_i(m_1, m_2; 0) > 0$ (Remark B.3), by setting $a_j = 0$ in Equations (B.38b) and (B.38c) we immediately obtain $\frac{\partial}{\partial m_1}a_{1,1}^{\mathrm{int}} = 0$, and

$$\frac{\partial}{\partial m_1}a_{2,2}^{\mathrm{int}} = -\frac{D_2'\big(\bar{e} - (1+m_1)a_{2,2}^{\mathrm{int}}\big) - (1+m_1)a_{2,2}^{\mathrm{int}}D_2''\big(\bar{e} - (1+m_1)a_{2,2}^{\mathrm{int}}\big)}{B_2''\left(\bar{e}_2 - a_{2,2}^{\mathrm{int}}\right) - (1+m_1)^2D_2''\big(\bar{e} - (1+m_1)a_{2,2}^{\mathrm{int}}\big)}\,.$$

From Lemma B.1, $a_{1,2}^{\text{int}}$, is the unique solution of $\frac{\partial}{\partial a_1}\Pi_1\Big|_{a_1=0} = 0$, that is,

$$-B_1'(\bar{e}_1 - m_1 a_2) + (1+m_2)D_1'\big(\bar{e} - (1+m_1)a_2\big) = 0\,.$$

Implicitly differentiating with respect to m_1 gives

$$B_1''(\bar{e}_1 - m_1 a_2)\left(a_{1,2}^{\text{int}} + m_1\frac{\partial}{\partial m_1}a_{1,2}^{\text{int}}\right) - (1+m_2)D_1''\big(\bar{e} - (1+m_1)a_{1,2}^{\text{int}}\big)\left(a_{1,2}^{\text{int}} + (1+m_1)\frac{\partial}{\partial m_1}a_{1,2}^{\text{int}}\right) = 0\,,$$

and hence

$$\frac{\partial}{\partial m_1}a_{1,2}^{\text{int}} = -a_{1,2}^{\text{int}}\frac{B_1''\left(\bar{e}_1 - m_1 a_{1,2}^{\text{int}}\right) - (1+m_2)D_1''\big(\bar{e} - (1+m_1)a_{1,2}^{\text{int}}\big)}{m_1 B_1''\left(\bar{e}_1 - m_1 a_{1,2}^{\text{int}}\right) - (1+m_1)(1+m_2)D_1''\big(\bar{e} - (1+m_1)a_{1,2}^{\text{int}}\big)} < 0\,.$$

Similarly, $a_{2,1}^{\text{int}}$ is the unique solution of

$$-B_2'(\bar{e}_2 - m_2 a_1) + (1+m_1)D_2'\big(\bar{e} - (1+m_2)a_1\big) = 0\,,$$

and implicitly differentiating with respect to m_1 gives

$$m_2 B_2''(\bar{e}_2 - m_2 a_{2,1}^{\text{int}})\frac{\partial}{\partial m_1}a_{2,1}^{\text{int}} + D_2'\big(\bar{e} - (1+m_2)a_{2,1}^{\text{int}}\big) - (1+m_1)(1+m_2)D_2''\big(\bar{e} - (1+m_2)a_{2,1}^{\text{int}}\big)\frac{\partial}{\partial m_1}a_{2,1}^{\text{int}} = 0\,,$$

so

$$\frac{\partial}{\partial m_1}a_{2,1}^{\text{int}} = -\frac{D_2'\big(\bar{e} - (1+m_2)a_{2,1}^{\text{int}}\big)}{m_2 B_2''\left(\bar{e}_2 - m_2 a_{2,1}^{\text{int}}\right) - (1+m_1)(1+m_2)D_2''\left(\bar{e} - (1+m_2)a_{2,1}^{\text{int}}\right)} > 0\,.$$

Interchanging countries 1 and 2 yields similar formulae for the derivatives of $a_{1,1}^{\text{int}}$ $a_{1,2}^{\text{int}}$, $a_{2,1}^{\text{int}}$ and $a_{2,2}^{\text{int}}$. □

C.10.4 Derivative of country 1's payoff with respect to its own matching factor, when only country 2 abates unconditionally

In this appendix, we prove the following lemma:

Lemma C.6. *If $m_1 > 0$ then*

$$\begin{aligned}
\frac{\mathrm{d}}{\mathrm{d}m_1}\Pi_1\big(m_1, m_2, 0, a_{2,2}^{\text{int}}(m_1,m_2)\big) = {} & \frac{1}{B_2''\left(\bar{e}_2 - a_{2,2}^{\text{int}}\right) - (1+m_1)^2 D_2''\left(\bar{e} - A\right)} \times \\
& \Big\{\big[(1+m_2)D_1'\left(\bar{e} - A\right) - B_1'\left(\bar{e}_1 - A_1\right)\big]\big[a_{2,2}^{\text{int}}B_2''\left(\bar{e}_2 - a_{2,2}^{\text{int}}\right) - m_1 D_2'\left(\bar{e} - A\right)\big] \\
& AB_1'\left(\bar{e}_1 - A_1\right)D_2''\left(\bar{e} - A\right) - m_2 a_{2,2}^{\text{int}} D_1'\left(\bar{e} - A\right)B_2''\left(\bar{e}_2 - a_{2,2}^{\text{int}}\right) \\
& - (1 - m_1 m_2)D_1'\left(\bar{e} - A\right)D_2'\left(\bar{e} - A\right)\Big\}\,.
\end{aligned}$$

In particular, if in addition $m_1 m_2 \geq 1$ and $a_{2,2}^{\text{int}} > a_{1,2}^{\text{int}}$, then

$$\frac{\mathrm{d}}{\mathrm{d}m_1}\Pi_1\big(m_1, m_2, 0, a_{2,2}^{\text{int}}(m_1,m_2)\big) < 0\,. \tag{C.83}$$

Proof. If the countries play $(a_1, a_2) = (0, a_{2,2}^{\text{int}})$ in stage II, then

$$\begin{aligned}
A_1 &= m_1 a_{2,2}^{\text{int}}\,, \\
A_2 &= a_{2,2}^{\text{int}}\,, \\
A &= (1+m_1)a_{2,2}^{\text{int}}\,.
\end{aligned}$$

Using Lemma C.5

$$\frac{\partial}{\partial m_1} a_{2,2}^{\text{int}} = -\frac{D_2'\big(\bar{e} - A\big) - AD_2''\big(\bar{e} - A\big)}{B_2''\left(\bar{e}_2 - a_{2,2}^{\text{int}}\right) - (1 + m_1)^2 D_2''\big(\bar{e} - A\big)},$$

so using $A = (1 + m_1) a_{2,2}^{\text{int}}$, it follows that

$$\begin{aligned}
\frac{\partial}{\partial m_1} A_1 &= a_{2,2}^{\text{int}} + m_1 \frac{\partial}{\partial m_1} a_{2,2}^{\text{int}} \\
&= a_{2,2}^{\text{int}} \frac{B_2''\left(\bar{e}_2 - a_{2,2}^{\text{int}}\right) - (1 + m_1)^2 D_2''\big(\bar{e} - A\big)}{B_2''\left(\bar{e}_2 - a_{2,2}^{\text{int}}\right) - (1 + m_1)^2 D_2''\big(\bar{e} - A\big)} \\
&\quad - m_1 \frac{D_2'\big(\bar{e} - A\big) - AD_2''\big(\bar{e} - A\big)}{B_2''\left(\bar{e}_2 - a_{2,2}^{\text{int}}\right) - (1 + m_1)^2 D_2''\big(\bar{e} - A\big)} \\
&= \frac{a_{2,2}^{\text{int}} B_2''\left(\bar{e}_2 - a_{2,2}^{\text{int}}\right) - AD_2''\big(\bar{e} - A\big) - m_1 D_2'\big(\bar{e} - A\big)}{B_2''\left(\bar{e}_2 - a_{2,2}^{\text{int}}\right) - (1 + m_1)^2 D_2''\big(\bar{e} - A\big)} > 0\,,
\end{aligned}$$

$$\begin{aligned}
\frac{\partial}{\partial m_1} A &= a_{2,2}^{\text{int}} + (1 + m_1) \frac{\partial}{\partial m_1} a_{2,2}^{\text{int}} \\
&= a_{2,2}^{\text{int}} \frac{B_2''\left(\bar{e}_2 - a_{2,2}^{\text{int}}\right) - (1 + m_1)^2 D_2''\big(\bar{e} - A\big)}{B_2''\left(\bar{e}_2 - a_{2,2}^{\text{int}}\right) - (1 + m_1)^2 D_2''\big(\bar{e} - A\big)} \\
&\quad - (1 + m_1) \frac{D_2'\big(\bar{e} - A\big) - AD_2''\big(\bar{e} - A\big)}{B_2''\left(\bar{e}_2 - a_{2,2}^{\text{int}}\right) - (1 + m_1)^2 D_2''\big(\bar{e} - A\big)} \\
&= \frac{a_{2,2}^{\text{int}} B_2''\left(\bar{e}_2 - a_{2,2}^{\text{int}}\right) - (1 + m_1) D_2'\big(\bar{e} - A\big)}{B_2''\left(\bar{e}_2 - a_{2,2}^{\text{int}}\right) - (1 + m_1)^2 D_2''\big(\bar{e} - A\big)} > 0\,.
\end{aligned}$$

It follows from Equation (A.25) that

$$\begin{aligned}
\frac{\mathrm{d}}{\mathrm{d}m_1} \Pi_1 &= -B_1'\left(\bar{e}_1 - A_1\right) \frac{\partial}{\partial m_1} A_1 + D_1'\left(\bar{e} - A\right) \frac{\partial}{\partial m_1} A \\
&= -B_1'\left(\bar{e}_1 - A_1\right) \frac{a_{2,2}^{\text{int}} B_2''\left(\bar{e}_2 - a_{2,2}^{\text{int}}\right) - AD_2''\big(\bar{e} - A\big) - m_1 D_2'\big(\bar{e} - A\big)}{B_2''\left(\bar{e}_2 - a_{2,2}^{\text{int}}\right) - (1 + m_1)^2 D_2''\big(\bar{e} - A\big)} \\
&\quad + D_1'\left(\bar{e} - A\right) \frac{a_{2,2}^{\text{int}} B_2''\left(\bar{e}_2 - a_{2,2}^{\text{int}}\right) - (1 + m_1) D_2'\big(\bar{e} - A\big)}{B_2''\left(\bar{e}_2 - a_{2,2}^{\text{int}}\right) - (1 + m_1)^2 D_2''\big(\bar{e} - A\big)} \\
&= \frac{1}{B_2''\left(\bar{e}_2 - a_{2,2}^{\text{int}}\right) - (1 + m_1)^2 D_2''\big(\bar{e} - A\big)} \times \\
&\quad \Big\{ \big[(1 + m_2) D_1'\left(\bar{e} - A\right) - B_1'\left(\bar{e}_1 - A_1\right)\big] \big[a_{2,2}^{\text{int}} B_2''\left(\bar{e}_2 - a_{2,2}^{\text{int}}\right) - m_1 D_2'\left(\bar{e} - A\right)\big] \\
&\quad AB_1'\left(\bar{e}_1 - A_1\right) D_2''\left(\bar{e} - A\right) - m_2 a_{2,2}^{\text{int}} D_1'\left(\bar{e} - A\right) B_2''\left(\bar{e}_2 - a_{2,2}^{\text{int}}\right) \\
&\quad - (1 - m_1 m_2) D_1'\left(\bar{e} - A\right) D_2'\left(\bar{e} - A\right) \Big\}\,.
\end{aligned} \tag{C.85}$$

To see that if (m_1, m_2) such that $m_1 m_2 \geq 1$ and $a_{2,2}^{\text{int}}(m_1, m_2) > a_{1,2}^{\text{int}}(m_1, m_2)$ the Equation (C.83) holds, note that since the denominator in Equation (C.85) is negative, we must only show that the numerator of Equation (C.85) (in curly brackets) is positive.

First, observe that since $m_1 m_2 \geq 1$

$$-(1 - m_1 m_2) D_1'\left(\bar{e} - A\right) D_2'\left(\bar{e} - A\right) \geq 0\,.$$

Next, since $B_i' > 0$, $B_i'' < 0$, $D_i' > 0$ and $D_i'' > 0$ for $i = 1, 2$,

$$AB_1'\left(\bar{e}_1 - A_1\right) D_2''\left(\bar{e} - A\right) - m_2 a_{2,2}^{\text{int}} D_1'\left(\bar{e} - A\right) B_2''\left(\bar{e}_2 - a_{2,2}^{\text{int}}\right) > 0\,.$$

It remains to show that

$$\left[(1+m_2)D_1'\left(\bar{e}-A\right)-B_1'\left(\bar{e}_1-A_1\right)\right]\left[a_{2,2}^{\text{int}}B_2''\left(\bar{e}_2-a_{2,2}^{\text{int}}\right)-m_1D_2'\left(\bar{e}-A\right)\right]\geq 0\,,$$

which we do by showing that the terms in square brackets are non-positive.

To see this, observe that because $B_i''<0$ and $D_i'>0$

$$a_{2,2}^{\text{int}}B_2''\left(\bar{e}_2-a_{2,2}^{\text{int}}\right)-m_1D_2'\left(\bar{e}-A\right)<0\,.$$

Now, note that $m_1,m_2>0$ (because $m_1m_2\geq 1$) and $a_{2,2}^{\text{int}}(m_1,m_2)>a_{1,2}^{\text{int}}(m_1,m_2)$. It follows from Remark B.5 that $(a_1,a_2)=(0,a_{2,2}^{\text{int}})$ is a Nash equilibrium, and hence country 1 cannot increase its payoff by increasing a_1, so

$$\frac{\partial}{\partial a_1}\Pi_1(m_1,m_2,a_1,a_2)\bigg|_{\substack{a_1=0\\a_2=a_{2,2}^{\text{int}}}}=-B_1'\left(\bar{e}_1-A_1\right)+(1+m_2)D_1'\left(\bar{e}-A\right)\leq 0\,,$$

so Equation (C.83) holds as claimed. □

C.11 Uniqueness of equilibrium matching factors (proof of Proposition B.18)

In this appendix, we show that for any $(\mu_1,\mu_2)\in\mathbb{R}^2_{\geq 0}\setminus\{(m_1^{\text{c}},m_2^{\text{c}})\}$ and $\big(a_1\left(m_1,m_2\right),a_2\left(m_1,m_2\right)\big)\in\mathcal{A}$, the strategy profile

$$\big(\mu_i,a_i\left(m_1,m_2\right)\big)\quad\text{for } i=1,2\,,\tag{C.86}$$

is *not* a SPE[9]. To do so, we divide the space of possible matching factors the five regions[10] schematically drawn in Figure C.2,

$$\mathcal{M}_1=\left\{(m_1,m_2)\in\mathbb{R}^2_{\geq 0}\;\middle|\;m_1m_2>1\,,m_1\leq\phi_1(m_2)\text{ and }m_2\leq\phi_2(m_1)\right\}\,,\tag{C.87a}$$
$$\mathcal{M}_2=\left\{(m_1,m_2)\in\mathbb{R}^2_{\geq 0}\;\middle|\;m_1>\phi_1(m_2)\text{ and }m_2>\phi_2(m_1)\right\}\,,\tag{C.87b}$$
$$\mathcal{M}_3=\left\{(m_1,m_2)\in\mathbb{R}^2_{\geq 0}\;\middle|\;0\leq m_2<m_2^{\text{c}}\text{ and }m_1\leq\phi_1(m_2)\right\}\,,\tag{C.87c}$$
$$\mathcal{M}_4=\left\{(m_1,m_2)\in\mathbb{R}^2_{\geq 0}\;\middle|\;m_1m_2<1\text{ and }m_2\geq m_2^{\text{c}}\right\}\,,\tag{C.87d}$$
$$\mathcal{M}_5=\left\{(m_1,m_2)\in\mathbb{R}^2_{\geq 0}\;\middle|\;m_1m_2\geq 1\text{ and }m_2>\phi_2(m_1)\right\}\,,\tag{C.87e}$$

where ϕ_i $(i=1,2)$ are the stage-II delimiter curves. In Appendices C.11.1 to C.11.5 we show that profitable unilateral deviations from matching factors in each of these regions exist; these profitable deviations are summarized in Figure C.3.

Also, note that on any equilibrium path $(m_1^{\text{c}},m_2^{\text{c}})$ is played in stage I. Recall from Lemma B.14 that if the matching factors $(m_1^{\text{c}},m_2^{\text{c}})$ are played in stage I, then both countries' abatements are independent of which Nash equilibrium is chosen in stage II, so it follows that the countries' abatements and payoffs on an equilibrium path are independent of which SPE is chosen.

In the following proofs, we will simplify notation by letting

$$\Psi_i=\frac{\partial}{\partial a_i}\Pi_i=-B_i'\big(\bar{e}_i-A_i\big)+(1+m_j)D_i'\big(\bar{e}-A\big)\,,\tag{C.88}$$

for $i=1,2$, so that

$$\frac{\partial}{\partial a_i}\Psi_i=B_i''\big(\bar{e}_i-A_i\big)-(1+m_j)^2D_i''\big(\bar{e}-A\big)<0\,,\tag{C.89a}$$
$$\frac{\partial}{\partial a_j}\Psi_i=m_iB_i''\big(\bar{e}_i-A_i\big)-(1+m_i)(1+m_j)D_i''\big(\bar{e}-A\big)<0\,,\tag{C.89b}$$
$$\frac{\partial}{\partial m_i}\Psi_i=a_j\Big(B_i''\big(\bar{e}_i-A_i\big)-(1+m_j)D_i''\big(\bar{e}-A\big)\Big)\leq 0\,,\tag{C.89c}$$
$$\frac{\partial}{\partial m_j}\Psi_i=D_i'\big(\bar{e}-A\big)-a_i(1+m_j)D_i''\big(\bar{e}-A\big)\,.\tag{C.89d}$$

[9]Note that for any $(\mu_1,\mu_2)\in\mathbb{R}^2_{\geq 0}$, if $\big(a_1\left(m_1,m_2\right),a_2\left(m_1,m_2\right)\big)\notin\mathcal{A}$, then the strategy profile given by Equation (C.86) is not an SPE.

[10]The arguments ruling out the matching factors in regions $\mathcal{M}_3$, $\mathcal{M}_4$ and $\mathcal{M}_5$ in Figure C.2 from being played on an SPE path apply (*mutatis mutandis*) to their symmetric counterparts.

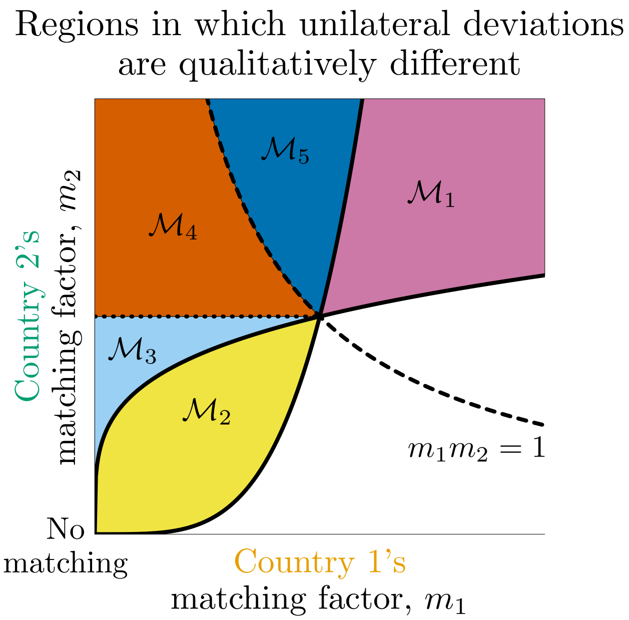

Figure C.2: Different regions of the space of matching factors for which unilateral deviations yield qualitatively different outcomes, as listed in Equation (C.87). The horizontal dotted line is at height $m_2 = m_2^{\mathrm{c}}$. The dashed curve (separating $\mathcal{M}_4$ from $\mathcal{M}_5$) is the locus of pairs of reciprocal matching factors, $\mathcal{M}_{\mathrm{r}}$ (i.e., those satisfying $m_1 m_2 = 1$). The solid black curves separating $\mathcal{M}_2$ from $\mathcal{M}_3$, and $\mathcal{M}_1$ from $\mathcal{M}_5$, are the stage-II delimiter curves ϕ_1 and ϕ_2, respectively.

Lastly, in Appendix C.11.6 we discuss a flaw in the argument given by Boadway et al. [1] to justify their claim that the SPE they find is unique.

C.11.1 Region 1

Here, we show that for *any* stage-II equilibrium choice,

$$\big(a_1\left(\mu_1, \mu_2\right), a_2\left(\mu_1, \mu_2\right)\big) \in \mathcal{A} ,$$

no pair of matching factors $(m_1, m_2) \in \mathcal{M}_1$ lies on the equilibrium path of an SPE[11].

Suppose that $(m_1, m_2) \in \mathcal{M}_1$, so that either two or three stage-II equilibria exist: $\left(0, a_{2,2}^{\mathrm{int}}\right)$, $\left(a_{1,1}^{\mathrm{int}}, 0\right)$ and possibly the interior equilibrium $\left(a_1^*(m_1, m_2), a_2^*(m_1, m_2)\right)$. In particular, $a_i\left(m_1, m_2\right) \neq 0$ for at least one country i ($i = 1$ or 2). From Proposition C.3,

$$\Pi_1\left(m_1, m_2, a_{1,1}^{\mathrm{int}}, 0\right) > \Pi_1\left(m_1, m_2, a_1^*, a_2^*\right) > \Pi_1\left(m_1, m_2, 0, a_{2,2}^{\mathrm{int}}\right) , \tag{C.90a}$$

$$\Pi_2\left(m_1, m_2, 0, a_{2,2}^{\mathrm{int}}\right) > \Pi_2\left(m_1, m_2, a_1^*, a_2^*\right) > \Pi_2\left(m_1, m_2, a_{1,1}^{\mathrm{int}}, 0\right) , \tag{C.90b}$$

whereas if $(m_1, m_2) \neq (m_1^{\mathrm{c}}, m_2^{\mathrm{c}})$ is on the boundary $\partial\,\mathcal{M}_1$,

$$\Pi_1\left(m_1, m_2, a_{1,1}^{\mathrm{int}}, 0\right) > \Pi_1\left(m_1, m_2, 0, a_{2,2}^{\mathrm{int}}\right) , \tag{C.91a}$$

$$\Pi_2\left(m_1, m_2, 0, a_{2,2}^{\mathrm{int}}\right) > \Pi_2\left(m_1, m_2, a_{1,1}^{\mathrm{int}}, 0\right) . \tag{C.91b}$$

If $a_2\left(m_1, m_2\right) \neq 0$, then country 1 can lower its matching factor unilaterally to m_1' (e.g., $m_1' = 0$) such that only the equilibrium $(a_{1,1}^{\mathrm{int}}, 0)$ exists for (m_1', m_2), and hence

$$\big(a_1\left(m_1', m_2\right), a_2\left(m_1', m_2\right)\big) = \left(a_{1,1}^{\mathrm{int}}\left(m_1', m_2\right), 0\right) .$$

[11] This claim is also made by Boadway et al. [1]. However, they only consider the situation in which the interior equilibrium is played in the entirety of $\mathcal{M}_1$, in which case each country's payoff increases with its matching factor (see Footnote 12 and Appendix C.11.6).

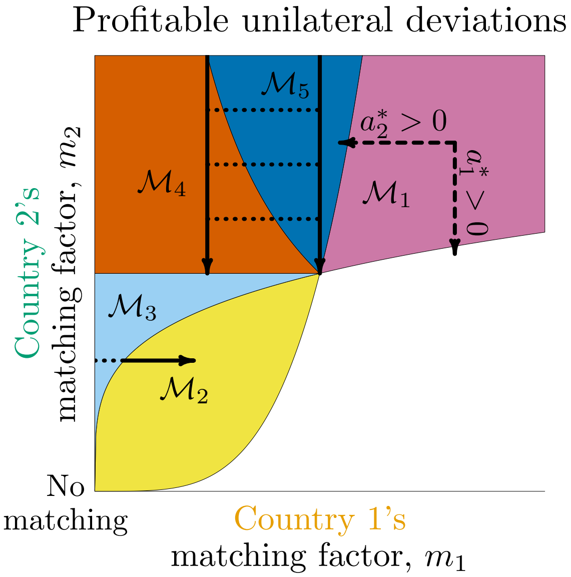

Figure C.3: Profitable unilateral deviations in different regions of the space of matching factors (defined in Equation (C.87) and analyzed in Appendices C.11.1 to C.11.5). Region $\mathcal{M}_1$: Which deviations are profitable depends on the equilibrium played at the chosen matching factors (which may be interior or on either edge). If country i abates unconditionally at the equilibrium, country j may increase its payoff by decreasing its matching factor so that country i abates only conditionally (although country payoffs need not be monotonic along the dashed lines). Regions $\mathcal{M}_2$ through $\mathcal{M}_5$: Country 1's payoff is constant along the dotted lines and increases with its matching factor m_1 on the solid horizontal line. Country 2's payoff decreases with its matching factor m_2 along the vertical solid lines. The arrows indicate unilateral profitable deviations.

Using Lemma C.5, for all $\mu_1 > 0$

$$\frac{\partial}{\partial \mu_1}\Pi_1\big(\mu_1, m_2, a_{1,1}^{\text{int}}(\mu_1, m_2), 0\big) = \frac{\partial}{\partial \mu_1}\Big[B_1\left(\bar{e}_1 - a_{1,1}^{\text{int}}(\mu_1, m_2)\right) - D_1\big(\bar{e} - (1+m_2)a_{1,1}^{\text{int}}(\mu_1, m_2)\big)\Big] = 0\,,$$

so

$$\begin{aligned}\Pi_1\big(m_1', m_2, a_1\left(m_1', m_2\right), a_2\left(m_1', m_2\right)\big) &= \Pi_1\big(m_1', m_2, a_{1,1}^{\text{int}}\left(m_1', m_2\right), 0\big) \\ &= \Pi_1\left(m_1, m_2, a_{1,1}^{\text{int}}\left(m_1, m_2\right), 0\right) > \Pi_1\big(m_1, m_2, a_1\left(m_1, m_2\right), a_2\left(m_1, m_2\right)\big)\,.\end{aligned}$$

Thus, country 1 can increase its payoff by deviating unilaterally from (m_1, m_2).

If $a_2\left(m_1, m_2\right) = 0$ then $a_1\left(m_1, m_2\right) \neq 0$, and an analogous argument shows that country 2 can increase its payoff by unilaterally lowering its matching factor.

C.11.2 Region 2

Here, we show that any $(m_1, m_2) \in \mathcal{M}_2$ is not played on the equilibrium path of an SPE, because either country can increase its payoff by unilaterally increasing its matching factor.

Let $(m_1, m_2) \in \mathcal{M}_2$. Then the unique stage-II Nash equilibrium (a_1^*, a_2^*) is interior and satisfies the simultaneous equations

$$\Psi_i = \frac{\partial}{\partial a_i}\Pi_i = 0 \quad \text{for } i = 1, 2\,. \tag{C.92}$$

The left-hand side of Equation (C.92) has continuous partial derivatives (Lemma B.2), and its Jacobian matrix with respect to the unconditional abatements is

$$J = \begin{pmatrix} \frac{\partial}{\partial a_1}\Psi_1 & \frac{\partial}{\partial a_2}\Psi_1 \\ \frac{\partial}{\partial a_1}\Psi_2 & \frac{\partial}{\partial a_2}\Psi_2 \end{pmatrix}.$$

The Jacobian determinant is

$$\begin{aligned}\det J &= \frac{\partial}{\partial a_1}\Psi_1 \frac{\partial}{\partial a_2}\Psi_2 - \frac{\partial}{\partial a_2}\Psi_1 \frac{\partial}{\partial a_1}\Psi_2 \\ &= \left[B_1''\big(\bar{e}_1 - A_1\big) - (1+m_2)^2 D_1''\big(\bar{e} - A\big)\right]\left[B_2''\big(\bar{e}_2 - A_2\big) - (1+m_1)^2 D_2''\big(\bar{e} - A\big)\right] \\ &\quad - \left[m_2 B_2''\big(\bar{e}_2 - A_2\big) - (1+m_2)(1+m_1) D_2''\big(\bar{e} - A\big)\right]\left[m_1 B_1''\big(\bar{e}_1 - A_1\big) - (1+m_1)(1+m_2) D_1''\big(\bar{e} - A\big)\right] \\ &= (1 - m_1 m_2) B_1''\big(\bar{e}_1 - A_1\big) B_2''\big(\bar{e}_2 - A_2\big) \\ &\quad - B_1''\big(\bar{e}_1 - A_1\big)(1+m_1)^2 D_2''\left(\bar{e} - A\right) \\ &\quad + m_1 B_1''\big(\bar{e}_1 - A_1\big)(1+m_2)(1+m_1) D_2''\left(\bar{e} - A\right) \\ &\quad - (1+m_2)^2 D_1''\left(\bar{e} - A\right) B_2''\big(\bar{e}_2 - A_2\big) \\ &\quad + (1+m_1)(1+m_2) D_1''\left(\bar{e} - A\right) m_2 B_2''\big(\bar{e}_2 - A_2\big) \\ &= (1 - m_1 m_2)\Big[B_1''\big(\bar{e}_1 - A_1\big) B_2''\big(\bar{e}_2 - A_2\big) \\ &\qquad - (1+m_1) B_1''\big(\bar{e}_1 - A_1\big) D_2''\left(\bar{e} - A\right) \\ &\qquad - (1+m_2) B_2''\big(\bar{e}_2 - A_2\big) D_1''\left(\bar{e} - A\right)\Big]\,,\end{aligned} \tag{C.93}$$

and because $m_1 m_2 \neq 1$, $\det J \neq 0$ at the interior Nash equilibrium (a_1^*, a_2^*).

It follows from the implicit function theorem [6] that for any $(m_1, m_2) \in \mathcal{M}_2$ there exists a neighbourhood $N_{(m_1, m_2)}$ of (m_1, m_2) and continuously differentiable functions $a_i\left(\mu_1, \mu_2\right)$ $(i = 1, 2)$, defined on $N_{(m_1, m_2)}$ that satisfy Equation (C.92) there; moreover $J\big(\mu_1, \mu_2, a_1\left(\mu_1, \mu_2\right), a_2\left(\mu_1, \mu_2\right)\big)$ is invertible in $N_{(m_1, m_2)}$ and (with minor abuse of notation),

$$\frac{\partial}{\partial \mu_i}\begin{pmatrix} a_1 \\ a_2 \end{pmatrix} = -J^{-1}\,\frac{\partial}{\partial \mu_i}\begin{pmatrix} \Psi_1 \\ \Psi_2 \end{pmatrix}\Bigg|_{\substack{a_1 = a_1(\mu_1, \mu_2) \\ a_2 = a_2(\mu_1, \mu_2)}}.$$

Because for all $(\mu_1,\mu_2) \in N_{(m_1,m_2)}$, $\big(a_1(\mu_1,\mu_2), a_2(\mu_1,\mu_2)\big)$ satisfy Equation (C.92), these abatements are are interior stage-II Nash equilibria for the corresponding matching factors.

For brevity, henceforth and until the end of Appendix C.11.2, a_1, a_2, A_1, A_2 and A are implicitly evaluated at $(a_1,a_2) = \big(a_1(m_1,m_2), a_2(m_1,m_2)\big)$, and the damage and benefit functions D_i and B_i (and their derivatives) are evaluated at the total emissions $\overline{e} - A$ and country i's emissions $\overline{e}_i - A_i$, respectively.

$$J^{-1} = \frac{1}{\det J} \begin{pmatrix} \frac{\partial}{\partial a_2}\Psi_2 & -\frac{\partial}{\partial a_2}\Psi_1 \\ -\frac{\partial}{\partial a_1}\Psi_2 & \frac{\partial}{\partial a_1}\Psi_1 \end{pmatrix},$$

so

$$\frac{\partial}{\partial m_i}\begin{pmatrix} a_1 \\ a_2 \end{pmatrix} = \frac{-1}{\det J} \begin{pmatrix} \frac{\partial}{\partial a_2}\Psi_2 & -\frac{\partial}{\partial a_2}\Psi_1 \\ -\frac{\partial}{\partial a_1}\Psi_2 & \frac{\partial}{\partial a_1}\Psi_1 \end{pmatrix} \frac{\partial}{\partial m_i}\begin{pmatrix} \Psi_1 \\ \Psi_2 \end{pmatrix} = \frac{1}{\det J}\begin{pmatrix} \frac{\partial}{\partial a_2}\Psi_1 \frac{\partial}{\partial m_i}\Psi_2 - \frac{\partial}{\partial a_2}\Psi_2 \frac{\partial}{\partial m_i}\Psi_1 \\ \frac{\partial}{\partial a_1}\Psi_2 \frac{\partial}{\partial m_i}\Psi_1 - \frac{\partial}{\partial a_1}\Psi_1 \frac{\partial}{\partial m_i}\Psi_2 \end{pmatrix}.$$

Substituting Equation (C.89) yields

$$\begin{aligned}
\det J \frac{\partial}{\partial m_1} a_1 &= -\frac{\partial}{\partial m_1}\Psi_1 \frac{\partial}{\partial a_2}\Psi_2 + \frac{\partial}{\partial a_2}\Psi_1 \frac{\partial}{\partial m_1}\Psi_2 \\
&= -a_2\Big(B_1'' - (1+m_2)\,D_1''\Big)\Big(B_2'' - (1+m_1)^2 D_2''\Big) \\
&\quad + \Big(m_1 B_1'' - (1+m_1)(1+m_2)\,D_1''\Big)\Big(D_2' - a_2(1+m_1)\,D_2''\Big) \\
&= -a_2 B_1'' B_2'' + a_2(1+m_1)^2 B_1'' D_2'' + a_2(1+m_2)\,D_1'' B_2'' - a_2(1+m_1)^2(1+m_2)\,D_1'' D_2'' \\
&\quad + m_1 B_1'' D_2' - a_2 m_1(1+m_1)\,B_1'' D_2'' - (1+m_1)(1+m_2)\,D_1'' D_2' + a_2(1+m_1)^2(1+m_2)\,D_1'' D_2'' \\
&= -a_2 B_1'' B_2'' + a_2(1+m_1)\,B_1'' D_2'' + a_2(1+m_2)\,B_2'' D_1'' + m_1 D_2' B_1'' - (1+m_1)(1+m_2)\,D_2' D_1'' < 0\,, \\
\det J \frac{\partial}{\partial m_1} a_2 &= -\frac{\partial}{\partial m_1}\Psi_2 \frac{\partial}{\partial a_1}\Psi_1 + \frac{\partial}{\partial a_1}\Psi_2 \frac{\partial}{\partial m_1}\Psi_1 \\
&= -\Big(D_2' - a_2(1+m_1)\,D_2''\Big)\Big(B_1'' - (1+m_2)^2 D_1''\Big) + \Big(m_2 B_2'' - (1+m_2)(1+m_1)\,D_2''\Big)a_2\Big(B_1'' - (1+m_2)\,D_1''\Big) \\
&= -D_2' B_1'' + (1+m_2)^2 D_2' D_1'' + a_2(1+m_1)\,B_1'' D_2'' - a_2(1+m_1)(1+m_2)^2 D_1'' D_2'' \\
&\quad + a_2 m_2 B_1'' B_2'' - a_2 m_2(1+m_2)\,B_2'' D_1'' - a_2(1+m_1)(1+m_2) B_1'' D_2'' + a_2(1+m_1)(1+m_2)^2 D_1'' D_2'' \\
&= -D_2' B_1'' + (1+m_2)^2 D_2' D_1'' + a_2 m_2 B_1'' B_2'' - a_2 m_2(1+m_2)\,B_2'' D_1'' - a_2 m_2(1+m_1)\,B_1'' D_2'' > 0\,.
\end{aligned}$$

It follows that

$$\begin{aligned}
\det J \frac{\partial}{\partial m_1} A_1 &= \det J\left(\frac{\partial}{\partial m_1} a_1 + a_2 + m_1 \frac{\partial}{\partial m_1} a_2\right) \\
&= \det J a_2 \\
&\quad - a_2 B_1'' B_2'' + a_2(1+m_1)\,B_1'' D_2'' + a_2(1+m_2)\,B_2'' D_1'' + m_1 D_2' B_1'' - (1+m_1)(1+m_2)\,D_2' D_1'' \\
&\quad + m_1\big[-D_2' B_1'' + (1+m_2)^2 D_2' D_1'' + a_2 m_2 B_1'' B_2'' - a_2 m_2(1+m_2)\,B_2'' D_1'' - a_2 m_2(1+m_1)\,B_1'' D_2''\big] \\
&= \det J a_2 - (1-m_1 m_2)\Big[a_2 B_1'' B_2'' - a_2(1+m_1)\,B_1'' D_2'' - a_2(1+m_2)\,B_2'' D_1'' + (1+m_2)\,D_2' D_1''\Big]\,,
\end{aligned}$$

and using Equation (C.93),

$$\begin{aligned}
\det J \frac{\partial}{\partial m_1} A_1 &= a_2(1-m_1 m_2)\Big[B_1'' B_2'' - (1+m_1) B_1'' D_2'' - (1+m_2) B_2'' D_1''\Big] \\
&\quad - (1-m_1 m_2)\Big[a_2 B_1'' B_2'' - a_2(1+m_1)\,B_1'' D_2'' - a_2(1+m_2)\,B_2'' D_1'' + (1+m_2)\,D_2' D_1''\Big] \\
&= -(1-m_1 m_2)(1+m_2)\,D_2' D_1''\,. \qquad \text{(C.94a)}
\end{aligned}$$

Similarly,

$$\begin{aligned}
\det J \frac{\partial}{\partial m_1} A_2 &= \det J \left(\frac{\partial}{\partial m_1} a_2 + m_2 \frac{\partial}{\partial m_1} a_1 \right) \\
&= -D_2' B_1'' + (1+m_2)^2 D_2' D_1'' + a_2 m_2 B_1'' B_2'' - a_2 m_2 (1+m_2) B_2'' D_1'' - a_2 m_2 (1+m_1) B_1'' D_2'' \\
&\quad + m_2 \big[- a_2 B_1'' B_2'' + a_2 (1+m_1) B_1'' D_2'' + a_2 (1+m_2) B_2'' D_1'' + m_1 D_2' B_1'' - (1+m_1)(1+m_2) D_2' D_1'' \big] \\
&= -(1-m_1 m_2) D_2' B_1'' + (1-m_1 m_2)(1+m_2) D_2' D_1'' = -(1-m_1 m_2) D_2' \big(B_1'' - (1+m_2) D_1'' \big) \\
&= -\frac{1-m_1 m_2}{a_2} D_2' \frac{\partial}{\partial m_1} \Psi_1 \,, \qquad \text{(C.94b)}
\end{aligned}$$

and

$$\begin{aligned}
\det J \frac{\partial}{\partial m_1} A &= \det J \frac{\partial}{\partial m_1} \big(A_1 + A_2 \big) \\
&= -(1-m_1 m_2)(1+m_2) D_2' D_1'' - (1-m_1 m_2) D_2' \big(B_1'' - (1+m_2) D_1'' \big) \\
&= -(1-m_1 m_2) D_2' B_1'' \,.
\end{aligned}$$

Note that since the unconditional abatements $\big(a_1(m_1,m_2), a_2(m_1,m_2)\big)$ are interior Nash equilibria,

$$B_1' = (1+m_2) D_1' \,,$$

(because $\Psi_1 = \frac{\partial}{\partial a_1} \Pi_1 = 0$, see Equation (B.36)), so

$$\begin{aligned}
\frac{\partial}{\partial m_1} \Pi_1(m_1,m_2) &= \frac{\partial}{\partial m_1} \big(B_1(\bar{e}_1 - A_1) - D_1(\bar{e} - A) \big) = -B_1' \frac{\partial}{\partial m_1} A_1 + D_1' \frac{\partial}{\partial m_1} A \\
&= -(1+m_2) D_1' \frac{\partial}{\partial m_1} A_1 + D_1' \frac{\partial}{\partial m_1} A = D_1' \left[\frac{\partial}{\partial m_1} A - (1+m_2) \frac{\partial}{\partial m_1} A_1 \right] \\
&= D_1' \left[\frac{\partial}{\partial m_1} A_2 - m_2 \frac{\partial}{\partial m_1} A_1 \right] .
\end{aligned}$$

Using Equations (C.94a) and (C.94b), we have

$$\begin{aligned}
\det J \left[\frac{\partial}{\partial m_1} A_2 - m_2 \frac{\partial}{\partial m_1} A_1 \right] &= -(1-m_1 m_2) D_2' \big(B_1'' - (1+m_2) D_1'' \big) + m_2 (1-m_1 m_2)(1+m_2) D_2' D_1'' \\
&= -(1-m_1 m_2) D_2' B_1'' + (1-m_2 m_1)(1+m_2)^2 D_2' D_1'' \\
&= -(1-m_1 m_2) D_2' \Big[B_1'' - (1+m_2)^2 D_1'' \Big] = -(1-m_1 m_2) D_2' \frac{\partial}{\partial a_1} \Psi_1 \,,
\end{aligned}$$

so,

$$\det J \frac{\partial}{\partial m_1} \Pi_1(m_1,m_2) = -(1-m_1 m_2) D_1' D_2' \frac{\partial}{\partial a_1} \Psi_1 \,.$$

Equation (C.93), then gives

$$\frac{\partial}{\partial m_1} \Pi_1(m_1,m_2) = \frac{-D_1' D_2' \Big[B_1'' - (1+m_2)^2 D_1'' \Big]}{B_1'' B_2'' - (1+m_1) B_1'' D_2'' - (1+m_2) B_2'' D_1''} > 0 \,. \qquad \text{(C.95)}$$

Analogously,

$$\frac{\partial}{\partial m_2} \Pi_2(m_1,m_2) > 0 \,.$$

To summarize, if the matching factors $(m_1,m_2) \in \mathbb{R}^2_{>0}$ yield a unique interior stage-II Nash equilibrium $\big(a_1(m_1,m_2), a_2(m_1,m_2)\big)$, then a unique interior Nash equilibrium exists for all (μ_1,μ_2) sufficiently close to

(m_1, m_2); if both countries play the unique interior Nash equilibrium near (m_1, m_2), then either country can increase its payoff by increasing its matching factor[12]. Because any $(m_1, m_2) \in \mathcal{M}_2$ yields a unique interior stage-II equilibrium, it follows immediately that no such matching factors are on an equilibrium path of an SPE.

C.11.3 Region 3

Here, we show that pairs of matching factors $(m_1, m_2) \in \mathcal{M}_3$ are not played on an SPE path.

If $(m_1, m_2) \in \mathcal{M}_3 \setminus \{(0,0)\}$, then

$$m_1 \leq \phi_1(m_2)\,.$$

For $\mu_1 \in [m_1, \phi_1(m_2)]$, the stage-II equilibrium is unique and independent of μ_1,

$$\big(a_1(\mu_1, m_2), a_1(\mu_1, m_2)\big) = (a_{1,1}^{\text{int}}, 0)\,,$$

(recall that from Lemma C.5, $\frac{\partial}{\partial \mu_1} a_{1,1}^{\text{int}} = 0$)[13].

Noting that $\phi_2^{-1}(m_2) > \phi_1(m_2)$ (from Appendix C.7.6, with the two country labels reversed), let $\Delta_1 = \phi_2^{-1}(m_2) - \phi_1(m_2) > 0$. It follows that $\big(\phi_1(m_2) + \delta\mu_1, m_2\big) \in \mathcal{M}_2$ for all $\delta\mu_1 \in (0, \Delta_1)$. From Equation (C.95), country 1's payoff increases with μ_1 on the open interval $\big(\phi_1(m_2), \phi_1(m_2) + \Delta_1\big)$, and hence it increases on $[\phi_1(m_2), \phi_1(m_2) + \Delta_1]$ (because $\big(a_1(\mu_1, m_2), a_1(\mu_1, m_2)\big)$, and hence $\Pi_1(\mu_1, m_2)$, are continuous in μ_1). Consequently,

$$\Pi_1(m_1, m_2) = \Pi_1(\phi_1(m_2), m_2) < \Pi_1(\phi_1(m_2) + \Delta_1, m_2) = \Pi_1\left(\phi_2^{-1}(m_2), m_2\right)\,,$$

so a profitable unilateral deviation exists for country 1.

Lastly, we have already seen that $(m_1, m_2) = 0$ is not on an SPE path (Proposition B.16).

C.11.4 Region 4

We will show that for all $(m_1, m_2) \in \mathcal{M}_4$, country 2 can increase its payoff by decreasing its matching factor, and thus (m_1, m_2) is not on the equilibrium path of an SPE.

For any $(m_1, m_2) \in \mathcal{M}_4$, if $\delta\mu_2 \in \mathbb{R}_{>0}$ is small enough then $(\mu_1, \mu_2) = (m_1, m_2 + \delta\mu_2)$ satisfies $\mu_1\mu_2 < 1$, $\mu_1 < m_1^{\text{c}}$ and $\mu_2 > m_2^{\text{c}}$, so $(\mu_1, \mu_2) \in \mathcal{M}_4$. Thus, $(a_{1,1}^{\text{int}}(\mu_1, \mu_2), 0)$ is the unique stage-II Nash equilibrium and country 2's payoff assuming equilibrium play in stage II is

$$\Pi_2(\mu_1, \mu_2) = B_2\left(\bar{e}_2 - \mu_2 a_{1,1}^{\text{int}}(\mu_1, \mu_2)\right) - D_2\left(\bar{e} - (1 + \mu_2) a_{1,1}^{\text{int}}(\mu_1, \mu_2)\right)\,.$$

Recall from Lemma C.4 (with the two country labels reversed) that if (m_1^{c}, μ_2) are played in stage I then since $\mu_2 > m_2^{\text{c}}$ the unique stage-II Nash equilibrium is $\big(a_{1,1}^{\text{int}}(m_1^{\text{c}}, \mu_2), 0\big)$, and so country 2's payoff assuming equilibrium play in stage II is

$$\Pi_2(m_1^{\text{c}}, \mu_2) = B_2\left(\bar{e}_2 - \mu_2 a_{1,1}^{\text{int}}(m_1^{\text{c}}, \mu_2)\right) - D_2\left(\bar{e} - (1 + \mu_2) a_{1,1}^{\text{int}}(m_1^{\text{c}}, \mu_2)\right)\,. \tag{C.96}$$

Since $a_{1,1}^{\text{int}}$ does not depend on m_1 (Lemma C.5), it follows that $\Pi_2(m_1, \mu_2) = \Pi_2(m_1^{\text{c}}, \mu_2)$. Moreover, Appendix C.11.4 holds in a neighbourhood of any $(m_1, m_2) \in \mathcal{M}_4$, and since $a_{1,1}^{\text{int}}$ is continuously differentiable at (m_1, m_2) (Lemma C.5),

$$\frac{\partial}{\partial m_2}\Pi_2(m_1, m_2) = \lim_{\mu_2 \to m_2^+} \frac{\partial}{\partial \mu_2}\Pi_2(m_1, \mu_2)\,.$$

[12] If countries choose $(m_1, m_2) \in \operatorname{int}\mathcal{M}_1$ in stage I, and if both countries play the unique *interior* stage-II equilibrium in a neighborhood of (m_1, m_2) then a similar derivation shows that Equation (C.95) still holds, and hence either country can increase its payoff by increasing its matching factor.

[13] If $m_1 = \phi_1(m_2)$, the interval $[m_1, \phi_1(m_2)]$ is degenerate, but this does not affect our argument.

Using Lemma C.6 (with the country labels reversed),

$$
\begin{aligned}
\frac{\partial}{\partial m_2}\Pi_2(m_1,m_2) &= \lim_{\mu_2\to m_2^+}\frac{\partial}{\partial \mu_2}\Pi_2\left(m_1,\mu_2\right) = \lim_{\mu_2\to m_2^+}\frac{\partial}{\partial \mu_2}\Pi_2\left(m_1^{\mathrm{c}},\mu_2\right)\\
&= \lim_{\mu_2\to m_2^+}\frac{1}{B_1''\left(\overline{e}_1-a_{1,1}^{\mathrm{int}}\right)-(1+\mu_2)^2D_1''\left(\overline{e}-A\right)}\times\\
&\quad \Big\{\left[(1+m_1^{\mathrm{c}})D_2'\left(\overline{e}-A\right)-B_2'\left(\overline{e}_2-A_2\right)\right]\left[a_{1,1}^{\mathrm{int}}B_1''\left(\overline{e}_1-a_{1,1}^{\mathrm{int}}\right)-m_1^{\mathrm{c}}D_1'\left(\overline{e}-A\right)\right]\\
&\quad\ AB_2'\left(\overline{e}_2-A_2\right)D_1''\left(\overline{e}-A\right)-m_1^{\mathrm{c}}a_{1,1}^{\mathrm{int}}D_2'\left(\overline{e}-A\right)B_1''\left(\overline{e}_1-a_{1,1}^{\mathrm{int}}\right)\\
&\quad\ -(1-\mu_2 m_1^{\mathrm{c}})D_1'\left(\overline{e}-A\right)D_2'\left(\overline{e}-A\right)\Big\}\,.
\end{aligned}
$$

Noting that $m_2 \geq m_2^{\mathrm{c}}$ implies that $m_1^{\mathrm{c}}m_2 \geq 1$, an argument similar to the proof of Lemma C.6 (specifically, the justification of Equation (C.83)) implies that the limit on the right hand side of the above equation is negative. It follows that country 2 can increase its payoff by unilaterally decreasing its matching factor m_2, and so (m_1, m_2) cannot be on the equilibrium path of an SPE[14].

C.11.5 Region 5

For $(m_1, m_2) \in \mathcal{M}_5$, $a_{1,1}^{\mathrm{int}} > a_{2,1}^{\mathrm{int}}$, and the only stage-II equilibrium is $(a_{1,1}^{\mathrm{int}}, 0)$ (Proposition B.9). Since $m_1m_2 \geq 1$, it follows from Lemma C.6 (reversing the roles of countries 1 and 2) that country 2 can increase its payoff by decreasing its matching factor, so such matching factors are not on the equilibrium path of an SPE.

C.11.6 On the argument given by Boadway et al. [1] for the uniqueness of the equilibrium

Boadway et al. [1] claim (in their proposition 1), that the matching-commitment agreement generates a unique SPE (up to a choice of stage-II equilibrium for the matching factors that yield two stage-II best-response functions that overlap for positive unconditional abatements). In this section, we focus on the argument for the uniqueness of the SPE, described in appendix A of Boadway et al. [1], and assume that the remainder of Boadway et al.'s argument holds. We show that this argument does not address the multiplicity of stage-II equilibria that occurs for some matching factors that are not played at subgame-perfect equilibria (similar to the multiplicity of stage-II Nash equilibria in $\mathcal{M}_1$), which is problematic for two reasons, described below.

For convenience, denote the pair of matching factors for which the two countries' stage-II best-response functions overlap[15] by $(m_1^{\mathrm{c}}, m_2^{\mathrm{c}})$. The first issue with Boadway et al.'s [1] argument, is that their Proposition 1 correctly identifies the SPE matching factors and total abatements, but neglects the fact that SPE are also defined by the unconditional abatements played for $(m_1, m_2) \neq (m_1^{\mathrm{c}}, m_2^{\mathrm{c}})$ (i.e., off of the equilibrium path). The multiplicity of stage-II Nash equilibria in $\mathcal{M}_1$ generates infinitely many possible SPEs in addition to those identified by Boadway et al., because each stage-II equilibrium choice yields a different SPE (similar to Proposition B.17).

Second, and more importantly, Boadway et al.'s justification for the claim that any $(m_1, m_2) \neq (m_1^{\mathrm{c}}, m_2^{\mathrm{c}})$ is not played on an SPE path remains problematic, even given a particular stage-II equilibrium choice,

$$
\left(a_1\left(\mu_1,\mu_2\right), a_2\left(\mu_1,\mu_2\right)\right) \in \mathcal{A}\,.
$$

To see this, it is useful to define the regions of the quadrant of non-negative matching factors, in which the effects of unilateral stage-I deviations differ, assuming Nash equilibria are played in stage II (similar to $\mathcal{M}_i$, $i = 1, \ldots, 5$, defined in Equation (C.87), in which we assumed that the baseline emissions profile $\overline{\boldsymbol{e}}$ is a Nash equilibrium of the BCG). For convenience, we use analogous notation here: $\mathcal{M}_1$ and $\mathcal{M}_2$ are the closures of the regions in which either both edge equilibria and an interior equilibrium exist, or only an

[14] Unfortunately, we cannot directly apply Lemma C.6 to show that $\frac{\partial}{\partial m_2}\Pi_2(m_1, m_2) < 0$ in this region (because $m_1m_2 < 1$).

[15] We stress that Boadway et al.'s argument justifying the existence of such matching factors is problematic, but can be completed into a rigorous proof (see Remark B.15).

interior equilibrium exists, respectively, both excluding the point (m_1^c, m_2^c). For matching factors (m_1, m_2) such that country 2 does not abate unconditionally at a stage-II Nash equilibrium, $\mathcal{M}_3$ is the region for which $(\mu_1, m_2) \in \mathcal{M}_2$ for some $\mu_1 > m_1$, $\mathcal{M}_4$ is the region for which $m_1 m_2 < 1$ and $(\mu_1, m_2) \in \mathcal{M}_1$ for some $\mu_1 > m_1$, and $\mathcal{M}_5$ is the region for which $m_1 m_2 \geq 1$ and $(\mu_1, m_2) \in \mathcal{M}_1$ for some $\mu_1 > m_1$. Note also that Boadway et al. argue that $\mathcal{M}_1$ (resp. $\mathcal{M}_2$) is above (below) the curve $m_1 m_2 = 1$, so Figure C.2 can be used as a graphical aid for the discussion that follows.

Boadway et al.'s argument is divided into the following cases[16] (which we reorder for convenience):

1. $m_1 m_2 > 1$:

   a. Boadway et al. claim that if $\left(a_1(m_1, m_2), a_2(m_1, m_2)\right)$ is interior (i.e., if $(m_1, m_2) \in \mathcal{M}_1$), then each country can increase its payoff by increasing its matching factor. They justify this by calculating the partial derivatives of the countries' payoffs with respect to their own matching factors, but in doing so implicitly assume that the interior equilibrium is played at all $(m_1 + \delta m_1, m_2)$ such that $|\delta m_1|$ is sufficiently small (that is, for small unilateral deviations by country 1). This need not be the case: for example, the equilibrium $(a_{2,2}^{\text{int}}, 0)$ may be played when $|\delta m_1| > 0$ but is sufficiently small, in which case the country payoffs are neither continuous, nor differentiable at (m_1, m_2), with respect to country 1's matching factor; moreover, in this case, a small change in country 1's matching factor would only lower its payoff (using an argument analogous to the proof of Equation (C.90)).

      In fact, any stage-II equilibrium choice yields payoffs that are discontinuous somewhere in $\mathcal{M}_1$. For example, suppose that the interior equilibrium is played in all of $\operatorname{int} \mathcal{M}_1$. Then, as $\mathcal{M}_5$ is approached from the interior of $\mathcal{M}_1$, the interior stage-II equilibrium approaches the edge-2 equilibrium, but when $\mathcal{M}_1$ is approached from $\mathcal{M}_5$, only the edge-1 equilibrium exists, so similar to Equation (C.91) (which also holds when the baseline emissions profile $\overline{\boldsymbol{e}}$ is not a Nash equilibrium of the BCG), both countries' payoffs are discontinuous at the border between $\mathcal{M}_1$ and $\mathcal{M}_5$. We will return to this discontinuity in case 2b below.

   b. If $\left(a_1(m_1, m_2), a_2(m_1, m_2)\right)$ is an edge 1 equilibrium (i.e., if $(m_1, m_2) \in \mathcal{M}_5$, or possibly $(m_1, m_2) \in \mathcal{M}_1$), Boadway et al. argue—also based on calculating the partial derivative of $\Pi_2(m_1, m_2)$ with respect to m_2—that country 2 can increase its payoff by decreasing its matching factor unilaterally. This argument holds in $\mathcal{M}_5$ (similar to our argument in Appendix C.11.5), but may fail in $\mathcal{M}_1$, depending on the specific stage-II equilibrium choice: for example, if $(m_1, m_2) \in \operatorname{int} \mathcal{M}_1$ and the interior equilibrium is played in a deleted neighbourhood of (m_1, m_2), then Π_2 is neither continuous nor differentiable at (m_1, m_2) with respect to m_2, and a small change in m_2 can only decrease country 2's payoff (similar to Equation (C.91)).

2. $m_1 m_2 < 1$: Note that when the stage-II equilibrium is on edge 1 (i.e., in $\mathcal{M}_3$ and $\mathcal{M}_4$) Boadway et al. argue that country 1's payoff remains unchanged if it increases its matching factor until country 2's "stage II first order condition is just binding"; denote country 1's matching factor when this happens by m_1'. They then consider two situations:

   a. If $m_1' m_2 < 1$, which occurs if $(m_1, m_2) \in \mathcal{M}_3$, then further increasing country 1's matching factor increases its payoff (similar to our argument in Appendix C.11.3).

   b. If $m_1' m_2 > 1$, which occurs if $(m_1, m_2) \in \mathcal{M}_4$, then Boadway et al. refer to their argument for case 1. This leaves some ambiguity as to their reasoning, in that they could be referring to case 1a or 1b; we discuss these two possibilities below.

      If case 1a is invoked, the argument is that country 1 can increase its payoff by increasing its matching factor further to some $\mu_1 > m_1'$ such that the interior equilibrium is played at $(\mu_1 + \delta\mu_1, m_2)$ for all sufficiently small $|\delta\mu_1|$. However, country 1's payoff is constant for all $(\mu_1, m_2) \in \mathcal{M}_4$ (because country 2 does not abate unconditionally), and is higher than its payoff

[16] Boadway et al. [1] consider stage-II Nash equilibria that are either interior, or on the a_2-axis, i.e., of the form $(0, a_{2,2}^{\text{int}})$. We rewrite their argument considering edge-1 equilibria—i.e., of the form $(a_{1,1}^{\text{int}}, 0)$—instead of the former, so that Figure C.2 can be used to develop intuition.

at either the a_2-edge equilibrium that exists when $(\mu_1, m_2) \in \mathcal{M}_1$, or the interior equilibrium that exists when $(\mu_1, m_2) \in \operatorname{int} \mathcal{M}_1$ (similar to Equation (C.90)). In addition, country 1's payoff at any $(\mu_1, m_2) \in \mathcal{M}_1$ such that the a_1-equilibrium is chosen in stage II is also equal to its original payoff, $\Pi_1(m_1, m_2)$. Thus, country 1 *cannot* increase its payoff beyond its value at the initial matching factors (m_1, m_2) in the way Boadway et al. describe, and hence they do not rule out such (m_1, m_2) being played at an SPE. The reason that the above version of Boadway et al.'s argument fails is that the stage-II equilibrium chosen (and thus country 1's payoff) must be discontinuous, somewhere along the section of the horizontal line passing at (m_1, m_2) that lies in $\mathcal{M}_1$. For example, choosing the interior equilibrium in all of $\operatorname{int} \mathcal{M}_1$ creates a discontinuity in the payoff $\Pi_1(\mu_1, \mu_2)$ at the boundary between $\mathcal{M}_5$ and $\mathcal{M}_1$ (see case 1a); even though country 1's payoff then increases with its matching factor μ_1 in the interior of $\mathcal{M}_1$, it remains lower than its original value, $\Pi_1(m_1, m_2)$.

If case 1b is invoked, the argument is that country 2 can then increase its payoff by decreasing its matching factor m_2. The problem here is that their argument relies on country 1 increasing its matching factor from m_1 to m_1', *and* country 2 decreasing its matching factor from m_2. In other words, rather than showing that one of the countries can increase its payoff by *unilaterally* changing its matching factor, this argument shows that there exist (μ_1, μ_2) at which country 2's payoff is higher than at (m_1, m_2) (assuming equilibrium play in stage II). Consequently, this argument does not exclude SPEs existing in $\mathcal{M}_4$.

Note also that cases 2a and 2b do not cover the possibility that $m_1' m_2 = 1$ (when $\overline{\boldsymbol{e}}$ is the Nash equilibrium of the BCG, this occurs on the boundary between $\mathcal{M}_3$ and $\mathcal{M}_4$). Hence, even ignoring the problems described above, Boadway et al.'s proof of the uniqueness of the SPE matching factors remains incomplete.

Thus, Boadway et al.'s argument does not justify the claim that the same pair of matching factors is played at all existing SPEs.

D Linear-algebraic lemmas

This appendix collects some simple results from linear algebra that are useful in our analysis of the basic climate game (Appendix A).

Lemma D.1. *For any integer $k \geq 2$, let*

$$A_k = \begin{pmatrix} 1, & \rho_1, & \dots, & \rho_1 \\ \rho_2, & 1, & \dots, & \rho_2 \\ \vdots & & \ddots & \vdots \\ \rho_k, & \dots, & \rho_k, & 1 \end{pmatrix} = \begin{pmatrix} \rho_1, & \dots, & \rho_1 \\ \vdots & & \vdots \\ \rho_k, & \dots, & \rho_k \end{pmatrix} - \operatorname{diag}(\rho_1, \dots, \rho_k) + I \,.$$

Then, $\det A_k > 0$ *for all* $(\rho_1, \dots, \rho_k) \in (0,1)^k$.

Proof. We prove our claim by induction. First, observe that since $\rho_1, \rho_2 \in (0,1)$

$$\det A_2 = \begin{vmatrix} 1, & \rho_1 \\ \rho_2, & 1 \end{vmatrix} = 1 - \rho_1 \rho_2 > 0 \,.$$

Now suppose our claim is true for all integers k such that $2 \leq k < K$. We will show that it also holds for $k = K$, which implies that our claim is true for all $k \geq 2$. We do this in three steps:

1. $\det A_K \neq 0$ for all $(\rho_1, \dots, \rho_K) \in (0,1)^K$;
2. $\det A_K$ has constant sign on $(0,1)^K$;
3. $\det A_K$ is positive for $(\rho_1, \dots, \rho_K) \in (0,1)^K$ sufficiently close to $\mathbf{0}$, and thus is positive in all $(0,1)^K$.

For ease of notation, denote $\boldsymbol{\rho}_k = (\rho_1, \ldots, \rho_k)$ (k an integer such that $2 \le k \le K$).

The first step is the most complicated. To prove it, we must show that A_K is invertible. By subtracting the vector $\rho_i \times (1, \rho_1, \ldots, \rho_1)$ (a multiple of the first row of A_K) from the ith row of A_K ($i = 2, \ldots, K$) and expanding along the first column we see that

$$\det A_K = \begin{vmatrix} 1, & \rho_1, & \ldots, & \rho_1 \\ 0, & 1-\rho_2\rho_1, & \ldots, & \rho_2(1-\rho_1) \\ \vdots & & \ddots & \vdots \\ 0, & \rho_K(1-\rho_1), & \ldots, & 1-\rho_K\rho_1 \end{vmatrix} = \begin{vmatrix} 1-\rho_2\rho_1, & \ldots, & \rho_2(1-\rho_1) \\ \vdots & \ddots & \vdots \\ \rho_K(1-\rho_1), & \ldots, & 1-\rho_K\rho_1 \end{vmatrix}. \tag{D.97}$$

Next, factoring $1 - \rho_i\rho_1$ out of the ith row on the right hand side of Equation (D.97), we obtain

$$\det A_K = \left[\prod_{i=2}^{K} (1-\rho_i\rho_1)\right] \times \begin{vmatrix} 1, & \frac{\rho_2(1-\rho_1)}{1-\rho_2\rho_1}, & \ldots, & \frac{\rho_2(1-\rho_1)}{1-\rho_2\rho_1} \\ \vdots & & \ddots & \vdots \\ \frac{\rho_K(1-\rho_1)}{1-\rho_K\rho_1}, & \ldots, & \frac{\rho_K(1-\rho_1)}{1-\rho_K\rho_1}, & 1 \end{vmatrix}. \tag{D.98}$$

Now,

$$\frac{\rho_i(1-\rho_1)}{1-\rho_i\rho_1} = \frac{1-\rho_i\rho_1}{1-\rho_i\rho_1} + \frac{\rho_i - 1}{1-\rho_i\rho_1} = 1 - \frac{1-\rho_i}{1-\rho_i\rho_1} \in (0,1),$$

because $\rho_1, \rho_i \in (0,1)$ implies that $\frac{1-\rho_i}{1-\rho_i\rho_1} \in (0,1)$. Consequently, the determinant on the right hand side of Equation (D.98) is of a matrix of the form

$$A_{K-1} = \begin{pmatrix} 1, & \rho'_1, & \ldots, & \rho'_1 \\ \rho'_2, & 1, & \ldots, & \rho'_2 \\ \vdots & & \ddots & \vdots \\ \rho'_{K-1}, & \ldots, & \rho'_{K-1}, & 1 \end{pmatrix},$$

with $(\rho'_1, \ldots, \rho'_K) \in (0,1)^{K-1}$. By our induction assumption, $\det A_{K-1} \neq 0$. Furthermore, $\prod_{i=2}^{K} (1-\rho_i\rho_1) \neq 0$ because $\boldsymbol{\rho}_K \in (0,1)^K$, so it follows that $\det A_K \neq 0$, justifying our first claim.

Since $\det A_K$ is a polynomial in $\rho_1, \ldots, \rho_K$, it is continuous. Thus, if there are two vectors $\boldsymbol{\rho}_K$ and $\boldsymbol{\rho}'_K$ in $(0,1)^K$ at which $\det A_K$ has opposite signs, then (because $(0,1)^K$ is convex) there is another vector $\boldsymbol{\rho}''_K \in (0,1)^K$ at which $\det A_K = 0$, contradicting step 1. Hence, $\det A_K$ has constant sign on $(0,1)^K$, justifying our second claim. Since $\det A_k$ is continuous and $\det A_k = 1$ when $\boldsymbol{\rho}_K = \mathbf{0}$, $\det A_K$ is positive in a neighbourhood of the origin, $\boldsymbol{\rho}_K = \mathbf{0}$. Since such a neighbourhood must intersect with $(0,1)^K$, it follows that $\det A_K > 0$ for all $\boldsymbol{\rho}_K \in (0,1)^K$ (confirming claim 3 and completing our proof). ☐

Corollary D.2. *For any $\alpha_1, \ldots, \alpha_n > 0$ and $\beta > 0$, the matrix*

$$A = \operatorname{diag}(\alpha_1, \ldots, \alpha_n) + \begin{pmatrix} \beta, & \ldots, & \beta \\ \vdots & & \vdots \\ \beta, & \ldots, & \beta \end{pmatrix},$$

is positive definite.

Proof. A's k^{th} leading principal minor ($1 \le k \le n$) is

$$\begin{vmatrix} \alpha_1 + \beta, & \ldots, & \beta \\ \vdots & & \vdots \\ \beta, & \ldots, & \alpha_k + \beta \end{vmatrix},$$

which can be rewritten as

$$\left[\prod_{i=1}^{k}(\alpha_i+\beta)\right]\times\det\begin{pmatrix}1, & \rho_1, & \dots, & \rho_1\\ \rho_2, & 1, & \dots, & \rho_2\\ \vdots & & \ddots & \vdots\\ \rho_k, & \dots, & \rho_k, & 1\end{pmatrix},$$

where

$$\rho_i=\frac{\beta}{\alpha_i+\beta}\in(0,1)\,.$$

By Lemma D.1, all of A's principal minors are positive, and because A is symmetric, it follows from Sylvester's criterion [9, p. 558] that it is positive definite. □

Lemma D.3. *Let $n\geq 2$, if $\alpha_1,\dots,\alpha_n>0$, $\beta_1,\dots,\beta_n\geq 0$, and*

$$A=\operatorname{diag}(\alpha_1,\dots,\alpha_n)-\begin{pmatrix}\beta_1, & \dots, & \beta_n\\ \vdots & & \vdots\\ \beta_1, & \dots, & \beta_n\end{pmatrix}.$$

If

$$\sum_{i=1}^{n}\frac{\beta_i}{\alpha_i}=1\,, \tag{D.99}$$

then A has rank $n-1$ and $\ker A=\operatorname{span}\left\{(\alpha_n/\alpha_1,\dots,\alpha_n/\alpha_{n-1},1)^{\mathrm{T}}\right\}$; otherwise, A is invertible (and thus $\operatorname{rank}A=n$ and $\ker A=\{\mathbf{0}\}$).

Proof. Subtracting the last row of A from all other rows, we get

$$\begin{pmatrix}\alpha_1, & 0, & \dots, & 0 & -\alpha_n\\ 0, & \alpha_2, & 0,\dots, & 0 & -\alpha_n\\ \vdots & & \ddots & & \vdots\\ 0, & \dots, & 0, & \alpha_{n-1}, & -\alpha_n\\ -\beta_1, & \dots, & & -\beta_n & \alpha_n-\beta_n\end{pmatrix},$$

and dividing row i by α_i for $i=1,\dots,n-1$ gives

$$\begin{pmatrix}1, & 0, & \dots, & 0 & -\alpha_n/\alpha_1\\ 0, & 1, & 0,\dots, & 0 & -\alpha_n/\alpha_2\\ \vdots & & \ddots & & \vdots\\ 0, & \dots, & 0, & 1, & -\alpha_n/\alpha_{n-1}\\ -\beta_1, & \dots, & & -\beta_{n-1} & \alpha_n-\beta_n\end{pmatrix}.$$

Eliminating the $n-1$ first elements of the last row (using multiples of the $n-1$ first rows) yields

$$\begin{pmatrix}1, & 0, & \dots, & 0 & -\alpha_n/\alpha_1\\ 0, & 1, & 0,\dots, & 0 & -\alpha_n/\alpha_2\\ \vdots & & \ddots & & \vdots\\ 0, & \dots, & 0, & 1, & -\alpha_n/\alpha_{n-1}\\ 0, & \dots, & & 0 & \alpha_n\left(1-\sum_{i=1}^{n-1}\frac{\beta i}{\alpha_i}\right)-\beta_n\end{pmatrix}.$$

If Equation (D.99) holds, then the last row of the matrix above is $\mathbf{0}$, so A has rank $n-1$ and

$$\ker A=\operatorname{span}\left\{(1/\alpha_1,\dots,,1/\alpha_n)^{\mathrm{T}}\right\}\;;$$

otherwise, A is manifestly invertible. □

References

[1] Boadway, R., Song, Z. & Tremblay, J.-F. The efficiency of voluntary pollution abatement when countries can commit. *Eur. J. Political Econ.* **27**, 352–368 (2011). URL https://doi.org/10.1016/j.ejpoleco.2010.10.003.

[2] Kolstad, C. D. & Mathiesen, L. Necessary and sufficient conditions for uniqueness of a Cournot equilibrium. *Rev. Econ. Stud.* **54**, 681–690 (1987). URL https://doi.org/10.2307/2297489.

[3] Folmer, H. & von Mouche, P. The acid rain game: a formal and mathematically rigorous analysis. In Kriström, B., Dasgupta, P. & Löfgren, K.-G. (eds.) *Economic theory for the environment: essays in honour of Karl-Göran Mäler.*, Contemporary Trends in European Social Sciences, 138–161 (Edward Elgar Publishing, 2002). URL https://books.google.ca/books?id=BuQkAQAAMAAJ.

[4] Simon, C. P. & Blume, L. *Mathematics for Economists* (Norton New York, 1994). URL https://books.google.at/books?id=cxSaQgAACAAJ.

[5] Samuelson, P. A. The pure theory of public expenditure. *Rev. Econ. Statist.* **36**, 387–389 (1954). URL http://www.jstor.org/stable/1925895.

[6] Thomson, B. S., Bruckner, J. B. & Bruckner, A. M. *Elementary Real Analysis* (www.classicalrealanalysis.com., 2008), 2 edn. URL https://books.google.at/books?id=vA9d57GxCKgC.

[7] Varian, H. R. *Microeconomic Analysis*. Norton International edition (Norton, 1992). URL https://books.google.at/books?id=m20iQAAACAAJ.

[8] Fudenberg, D. & Tirole, J. *Game Theory* (MIT Press, 1991).

[9] Meyer, C. D. *Matrix analysis and applied linear algebra*, vol. 71 (SIAM, 2000).
